# Reactivity‐Tunable Fluorescent Platform for Selective and Biocompatible Modification of Cysteine or Lysine

**DOI:** 10.1002/advs.202402838

**Published:** 2024-06-19

**Authors:** Xiaojie Ren, Haokun Li, Hui Peng, Yang Yang, Hang Su, Chen Huang, Xuan Wang, Jie Zhang, Zhiyang Liu, Wenyu Wei, Ke Cheng, Tianyang Zhu, Zhenpin Lu, Zhengqiu Li, Qian Zhao, Ben Zhong Tang, Shao Q. Yao, Xiangzhi Song, Hongyan Sun

**Affiliations:** ^1^ Department of Chemistry and Centre of Super‐Diamond and Advanced Films (COSDAF) City University of Hong Kong 83 Tat Chee Avenue, Kowloon Hong Kong 999077 China; ^2^ College of Chemistry & Chemical Engineering Central South University Changsha Hunan 410083 China; ^3^ International Cooperative Laboratory of Traditional Chinese Medicine Modernization and Innovative Drug Development (MOE) MOE Key Laboratory of Tumor Molecular Biology School of Pharmacy Jinan University Guangzhou Guangdong 510632 China; ^4^ Department of Applied Biology and Chemical Technology The Hong Kong Polytechnic University Hung Hom, Kowloon Hong Kong 999077 China; ^5^ Department of Chemistry National University of Singapore Singapore 117543 Singapore; ^6^ School of Pharmaceutical Sciences (Shenzhen) Shenzhen Campus of Sun Yat‐sen University Shenzhen 518107 China; ^7^ Department of Chemistry The Hong Kong University of Science and Technology Clear Water Bay, Kowloon Hong Kong 999077 China

**Keywords:** chemoselective modification, cysteine, HDACs, lysine, PDT

## Abstract

Chemoselective modification of specific residues within a given protein poses a significant challenge, as the microenvironment of amino acid residues in proteins is variable. Developing a universal molecular platform with tunable chemical warheads can provide powerful tools for precisely labeling specific amino acids in proteins. Cysteine and lysine are hot targets for chemoselective modification, but current cysteine/lysine‐selective warheads face challenges due to cross‐reactivity and unstable reaction products. In this study, a versatile fluorescent platform is developed for highly selective modification of cysteine/lysine under biocompatible conditions. Chloro‐ or phenoxy‐substituted NBSe derivatives effectively labeled cysteine residues in the cellular proteome with high specificity. This finding also led to the development of phenoxy‐NBSe phototheragnostic for the diagnosis and activatable photodynamic therapy of GSH‐overexpressed cancer cells. Conversely, alkoxy‐NBSe derivatives are engineered to selectively react with lysine residues in the cellular environment, exhibiting excellent anti‐interfering ability against thiols. Leveraging a proximity‐driven approach, alkoxy‐NBSe probes are successfully designed to demonstrate their utility in bioimaging of lysine deacetylase activity. This study also achieves integrating a small photosensitizer into lysine residues of proteins in a regioselective manner, achieving photoablation of cancer cells activated by overexpressed proteins.

## Graphical abstract

The reactivity of NBSe derivatives is tunable, enabling selective modification of cysteines and lysines. NBSe‐Cl/OPh can profile cysteines at the proteome level, and excute photoablation of cancer cells under glutathione activation. Conversely, alkoxy‐fused NBSe derivatives show minimal reactivity toward thiols, enabling precise visualization of HDAC activity in living cells and labeling proteins in a regioselective manner.

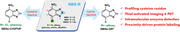

## 1 Introduction

Chemoselective modification of proteinogenic amino acids under physiological conditions has attracted increasing attention in recent years.^[^ ^1^ ^]^ The selective modification of specific sites within proteins offers tremendous possibilities for introducing a diverse range of functional tags into proteins.^[^ ^2^ ^]^ The versatility of site‐specific protein modification has propelled its widespread applications in drug discovery, bioimaging, biomaterials and biomedicine.^[^ ^3^ ^]^ Consequently, the quest to develop novel and efficient strategies for chemoselective protein modifications has become a vibrant and rapidly advancing field of research.^[^ ^4^ ^]^

Cysteine has been a prime target for chemoselective labeling under physiological conditions, facilitating the design of covalent inhibitors, biosensors, and probes used in chemical proteomics.^[^ ^5^ ^]^ A variety of chemical warheads have been devised for cysteine.^[^ ^6^ ^]^ Despite their usefulness, some of these cysteine warheads suffer from poor selectivity due to cross‐reactivity with other nucleophilic amino acids,^[^ ^7^ ^]^ and/or the hydrolytic instability of reaction products.^[^ ^8^ ^]^ Moreover, the low abundance of cysteine restricts the detectable signals from its modification, thereby impairing the resolution of measurements. Lysines are one of the most adundant amino acids in a protein, and have emerged as promising targets in recent years.^[^ ^9^ ^]^ Lysine serves as crucial functional sites in numerous proteins.^[^ ^10^ ^]^ However, achieving chemoselective modification of lysine residues in a protein poses a significant challenge. This challenge arises from their intrinsically low nucleophilicity which results from their protonation at physiological conditions.^[^ ^11^ ^]^ Consequently, many lysine‐reactive warheads also exhibit cross‐reactivity with other nucleophilic residues such as cysteine. Interests in the design of new chemical warheads capable of selectively reacting with cysteine or lysine residues under physiological conditions have grown rapidly. Because the microenvironments of cysteine and lysine in different proteins are variable, achieving high modification selectivity necessitates the warhead with specific reactivity. Constructing a universal molecular platform on which warheads with tunable reactivity can be devised is crucial for the selective labeling of various cysteines and lysines.

**Figure 1:**
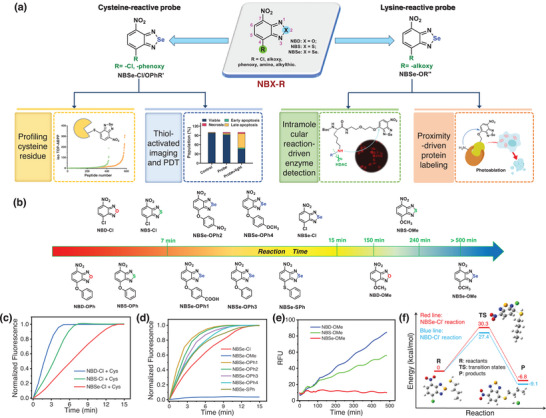
a) Fine‐tuning of NBX derivatives for chemoselective modification of cysteine and lysine under biocompatible conditions. b) Reaction times of NBX derivatives (10 µm) with Cys (100 µm) in PBS (pH 7.4, containing 20% DMSO). c) Fluorescence kinetics of NBD‐Cl, NBS‐Cl, and NBSe‐Cl with Cys, respectively. d) Kinetic study of NBSe‐derivatives with Cys. e) Kinetic study of NBD‐OMe, NBS‐OMe, and NBSe‐OMe with Cys, respectively. f) Computed energy profiles and transition states in the reaction of NBSe‐Cl (red curve) and NBD‐Cl (blue curve) with *n*‐butylthiol, respectively.

Nitrobenzoxadiazole (NBD) fluorescent dyes exhibit exceptional properties, including small molecular size, high quantum yield, and environment‐sensitive optical properties.^[^ ^12^ ^]^ They have been widely utilized in the design of fluorescent probes.^[^ ^13^ ^]^ A series of NBD‐based probes have been tailored to selectively detect H_2_S, biothiols in living organisms.^[^ ^14^ ^]^ Hence, we aimed to explore the potential of NBD derivatives for selective modification of cysteines at the proteome level. However, our investigations revealed that F, Cl, or OPh‐substituted NBD derivatives were ineffective in selective cysteine modification due to excessive reactivity, resulting in cross‐reacting with amino groups. Additionally, their thiolysis products emitted weak fluorescence signals, severely limiting their utility. Previously, we and others utilized alkoxy‐NBD probes to selectively label lysines following enzymatic removal of various post‐translational modifications (PTMs), including Kac, Klip, and Kcr.^[^ ^15^, ^16^, ^17^ ^]^ These alkoxy‐NBD probes, however, lack adequate selectivity in live‐cell applications due to the nucleophilic attack by intracellular thiols (such as GSH) and other reactive lysine residues in proteins (e.g., lysine within BSA). As a result, further biological applications of these reported NBD derivatives in chemoselective modification of cysteine and lysine need to be fine‐tuned. Priorly, the introduction of S and Se into NBD fluorophores yielded 7‐nitrobenzo[*c*]thiadiazol (NBS) and 7‐nitrobenzo[*c*]selenadiazole (NBSe) derivatives, respectively, which exhibit interesting optical properties.^[^ ^18^ ^]^ Research on the chemical reactivity of these NBD derivatives (including NBD, NBS and NBSe derivatives; herefter named NBX, **Figure** **1**a is relatively scarce.

Herein, we judiciously designed and synthesized a diverse library of NBX derivatives with O, S and Se heteroatoms at 2‐position and various substituted groups at 4‐position (NHR, SR, Cl, OPhR, and OR), respectively (Figure 1; Scheme S1, Supporting Information). After systematical investigation of their photophysical properties and the S_N_Ar reactivities with nucleophiles, we established a unique class of NBSe derivatives for chemoselective modification of cysteine or lysine in proteins, with promising applications in chemical proteomics. And their effectiveness was demonstrated by the biological applications in profiling reactive cysteine residues at the proteome level, precise modification of lysine via proximity strategies for detecting HDAC enzymatic activity, and specific tumor cell eradication by assembling an activatable photosensitizer into lysine residues of a protein in a regioselective manner.

## 2 Results and Discussion

### 2.1 Investigation with Small‐Molecule/Protein Thiols and Cysteine Proteome Profiling

Compared to NBD derivatives, their NBS and NBSe analogues show distinct photophysical properties. For example, NBSe‐S‐nBu displayed much stronger fluorescence with longer emission wavelength compared to NBD‐S‐nBu (Scheme S2 and Figures S1–S7, Supporting Information), owing to 4*d*‐function of Se atoms as a result of π‐hyperconjugation (Figure S12, Supporting Information).^[^ ^19^ ^]^ NBSe‐S‐nBu and NBSe‐N‐nBu also exhibited a large emission spectral separation, which presents an opportunity for NBSe‐based probes to differentiate amines and thiols using distinct fluorescence channels (Table S1, Supporting Information). We hypothesized that the electron distribution around the molecular framework of NBX could be used to fine‐tune the reactivities of NBS and NBSe. Consequently, with the synthesized NBX derivatives, we first examined the impact of heteroatoms at the 2‐position. Cl‐substituted NBSe‐Cl, NBS‐Cl, and NBD‐Cl were used as examples to react with the crucial biothiol, cysteine (Cys). The reaction of NBX‐Cl and Cys involves two steps: initially, the thiol group in Cys reacts with NBX‐Cl, forming NBX‐S‐Cys; subsequently, NBX‐S‐Cys undergoes an intramolecular Smile rearrangement reaction, yielding NBX‐N‐Cys (Scheme S3, Supporting Information). The reaction between NBD‐Cl and Cys showed rapid kinetics, with a prompt occurrence of an emission peaked at 550 nm, reaching equilibrium within 5 min (Figure 1c; Figure S16c, Supporting Information). In contrast, NBSe‐Cl reacted with Cys at a slower rate, resulting in a red‐emitting product achieving full conversion ≈14 min (Figure 1c; Figure S16a, Supporting Information). Meanwhile, the reaction of NBS‐Cl with Cys attained full conversion within 7 min (Figure 1c; Figure S16b, Supporting Information). To further assess the reactivity of NBX‐Cl, *n*‐butylthiol was used as a reactant to measure the second‐order rate constants (*k*). The rate constants were determined to be 13.75, 8.25, and 2.58 M^−1^ s^−1^ for NBD‐Cl, NBS‐Cl, and NBSe‐Cl, respectively (Figure S18 and Table S2, Supporting Information). Also, Density Functional Theory (DFT) calculations revealed that NBSe‐Cl could react with *n*‐butylthiol through a transition state with a higher activation energy barrier compared to NBD‐Cl and NBS‐Cl, providing further evidence of the diminished reactivity of NBSe‐Cl (Figure 1f; Figure S15, Supporting Information). These results implied that the reactivities of similar NBX derivatives with only heteroatom differences are in the order of NBD>NBS>NBSe.

Next, we observed the influence of the leaving group on the S_N_2 reaction of NBX derivatives with Cys. Different substitutes, chloro (‐Cl), alkoxy (‐OMe), and phenoxy (‐OPh) groups, with varying leaving abilities were introduced at the 4‐position of NBX derivatives (Figure 1b). It was seen that, for the series of NBX derivatives with the same heteroatom at 2‐position, their reactivities with Cys followed the order of NBX‐OPh ≈ NBX‐Cl >> NBX‐OR, roughly in line with the leaving ability of the substituents (Figure 1d; Figure S17, Supporting Information). But there was a discrepancy between the reactivities of NBX‐OPh and NBX‐Cl, especially for NBSe‐OPh and NBSe‐Cl, which was ascribed to the difference in their solubility. As expected, the NBX derivatives with the same substitute at 4‐position showed reactivities following the order of NBD>NBS>NBSe. It was noted that the substitution at the para‐position of the phenoxy substituents impacted the reactivity of an NBX derivative: an electron‐withdrawing group accelerated it whereas an electron‐donating group decreased it (Figure 1d; Figure S20, Supporting Information). All alkoxy‐substituted NBX derivatives displayed low reactivity: they required 80 min to obtain detectable signals after incubating NBD‐OMe/NBS‐OMe with Cys, and their fluorescence intensity steadily increased over an 8‐h test; NBSe‐OMe was nearly inert to Cys and no observable fluorescence signals were obtained after 8‐h incubation (Figure 1e). Overall, these results demonstrated the reactivity of NBX derivatives could be fine‐tuned by changing both the heteroatoms and the leaving groups.

**Figure 2:**
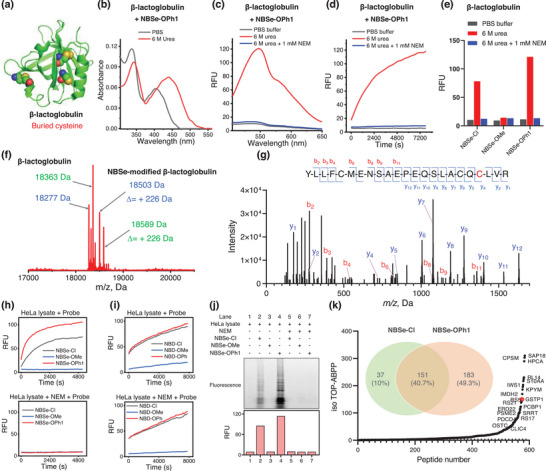
a) Folded structure of bovine *β*‐lactoglobulin with one buried cysteine (Cys121). b) Absorbance, c) fluorescence and d) kinetic study of NBSe‐OPh1 (10 µm) in response to *β*‐lactoglobulin in PBS. e) Fluorescence intensities of NBSe‐Cl, NBSe‐OMe, and NBSe‐OPh1 in response to *β*‐lactoglobulin in PBS for 2 h. Probes concentration: 10 µm. f) Intact protein MS and g) site‐mapping analysis of *β*‐lactoglobulin upon incubation with NBSe‐OPh1 in 6 m urea at room temperature for 1 h. h,i) Kinetic study of various NBSe and NBD derivatives in response to HeLa lysate in the absence or presence of NEM in PBS. j) In‐gel fluorescence labeling (upper) and fluorescence intensity (bottom) of HeLa cell lysates treated with NBSe‐Cl, NBSe‐OMe, or NBSe‐OPh1, respectively. Probes concentration: 10 µm; HeLa lysate: 1 mg mL^−1^; NEM concentration: 10 mm. k) IsoTOP‐ABPP analysis of HeLa lysates (1 mg mL^−1^) treated with NBSe‐OPh1 (50 µm) (inset: the proteins with a ratio of > 4, detected by NBSe‐Cl and NBSe‐OPh1). All experiments were carried out in 10 mm PBS (pH 7.4) containing 10% DMSO at room temperature.

Encouraged by the unique reactivity of NBSe derivatives, we next explored their potential to selectively react with protein thiols under biocompatible conditions. We focused on NBSe‐Cl, NBSe‐OMe, and NBSe‐OPh1 with two model proteins: bovine *β*‐lactoglobulin and GSTP1, harboring one (but buried) and two (solvent‐exposed) cysteine residues, respectively (**Figure** **2**a; Figure S25a, Supporting Information). As illustrated in Figures 2b‐e and S21 and S22 (Supporting Information), negligible fluorescence was seen from the mixture of NBSe‐Cl, NBSe‐OMe, or NBSe‐OPh1 with folded *β*‐lactoglobulin. However, upon denaturation (6 m urea), its mixture with NBSe‐Cl or NBSe‐OPh1 exhibited significant fluorescence increase with a maximum at 548 nm, respectively (Figure 2c; Figure S21, Supporting Information). The changes in the absorption spectra and kinetics curves further corroborated the effective reaction of NBSe‐Cl and NBSe‐OPh1 with its cysteine under denatured conditions (Figure 2b,d; Figure S21, Supporting Information). Addition of N‐ethylmaleimide (NEM; a thiol blocker) effectively prevented the fluorescence increase of NBSe‐Cl/NBSe‐OPh1 in the presence of unfolded protein, suggesting that these probes were highly specific toward cysteine residues (Figure 2e). Moreover, the reaction product of urea‐treated *β*‐lactoglobulin with NBSe‐OPh1 was analyzed using intact protein MS, which demonstrated that one cysteine reacted with probe (+226 Da, Figure 2f; Figure S23, Supporting Information). The product was further confirmed by site‐mapping to be the covalent adduct of Cys121 with the NBSe‐probe, indicating the regioselectivity of this reaction (Figure 2g; Figure S24, Supporting Information). A turn‐on fluorescence was seen in the mixture of native GSTP1 with NBSe‐Cl and NBSe‐OPh1 (Figures S25 and S26, Supporting Information). When GSTP1 was pre‐treated with NEM, however, no fluorescence or absorption color in the mixture was observed. In sharp contrast, NBSe‐OMe displayed no observable absorption or fluorescence response toward denatured *β*‐lactoglobulin (Figure 2e; Figure S22, Supporting Information) or GSTP1 (Figures S27 and S28, Supporting Information) under any condition due to its low reactivity. These results collectively showed that NBSe‐Cl and NBSe‐OPh1 selectivity reacted with cysteine residues in proteins with high specificity.

We next investigated the reactivity of NBSe derivatives toward cellular proteomes. HeLa cells were lysed and small molecules, including biothiols, were carefully removed by ultracentrifugation. The prepared HeLa lysates were then separately incubated with NBSe‐Cl, NBSe‐OMe, and NBSe‐OPh1 in PBS buffer. Notably, solutions containing NBSe‐Cl and NBSe‐OPh1 displayed significant increases in fluorescence, peaking at 546 nm (the characteristic emission peak of NBSe‐SR adducts), indicating reacting with cysteines in cellular proteins, respectively (Figure 2f; Figure S29, Supporting Information). However, no noticeable fluorescence enhancement was detected when the HeLa lysate was pre‐treated with NEM (Figure 2f, lower panel). In‐gel fluorescence scanning profiles of HeLa lysates revealed that NBSe‐Cl and NBSe‐OPh1 reacted with a wide range of cellular proteins, while NBSe‐OMe showed no reactivity with the cell lysate (Figure 2h; Figure S30, Supporting Information). Furthermore, we performed isotopic tandem orthogonal proteolysis protein profiling (isoTOP‐ABPP) study to assess cysteine reactivity of our probes in cellular proteomes (Figure 2i; Figure S34, Tables S3 and S4, Supporting Information); results indicated that ≈580 and 420 cysteine residues were identified to react with NBSe‐OPh1 and NBSe‐Cl, respectively, in human proteome of HeLa cells. In stark contrast, NBD‐Cl and NBD‐OPh in NEM‐treated cell lysate still showed significant fluorescence signals (NBD‐NR adducts), indicating their poor selectivity for targeting cysteines (Figure 2g; Figures S31–S33, Supporting Information). These observations collectively suggest that phenoxyl‐ and chloro‐NBSe probes were capable of labeling cysteines in proteins with a high selectivity, making them potential agents for chemoproteomic studies and drug development. In addition, the thiol‐addition product of the NBSe‐based probe has demonstrated high stability (Figures S35 and S36, Supporting Information), enhancing its practicality in the modification of cysteine in proteins.

### 2.2 Activatable Photoablation of Cancer Cells Through Endogenous GSH

**Figure 3:**
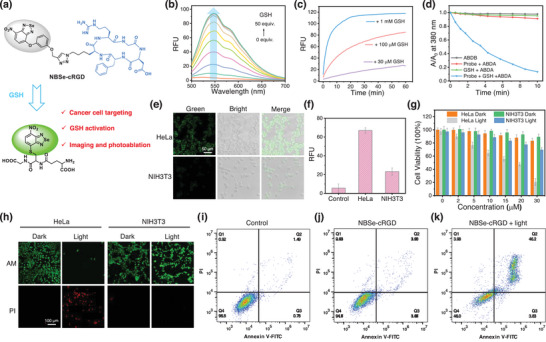
a) Chemical structure and response mechanism of NBSe‐cRGD toward GSH. b) fluorescence responses of NBSe‐cRGD toward GSH in PBS (pH 7.4) at room temperature. c) Time‐dependent fluorescence responses of NBSe‐cRGD toward different concentrations of GSH. d) Relative absorbance at 380 nm of ABDA (50 µm) incubated with different compounds (GSH: 500 µm; NBSe‐cRGD: 10 µm) in PBS under LED irradiation (440–470 nm, 30 mW cm^−2^). e) Fluorescence images of HeLa cells (top) and NIH3T3 cells (bottom) incubated with NBSe‐cRGD (10 µm) for 60 min. *λ*_ex_ = 488 nm; *λ*_em_ = 500–550 nm. f) Relative fluorescence intensity of cells in (e). g) Cell viability study and h) Calcein‐AM and propidium iodide (PI) staining of HeLa and NIH3T3 cells treated with NBSe‐cRGD (10 µm) in the dark and with LED irradiation (400–800 nm, 30 mW cm^−2^, 10 min). i,j,k) Cell apoptosis study by flow cytometry analysis of NBSe‐cRGD (10 µm) in HeLa cells after LED irradiation (400–800 nm, 30 mW cm^−2^, 15 min).

Amino‐ and thiol‐based NBSe derivatives are known to possess significant singlet oxygen‐generation ability.^[^ ^20^ ^]^ We thus tested our NBSe probes for potential photodynamic therapy (PDT) in GSH‐overexpressed cancer cells. GSH plays a crucial role in various metabolic processes and is over‐expressed in cancer cells.^[^ ^21^ ^]^ The pronounced affinity and selectivity of the cRGD peptide (cyclo‐arginine‐glycine‐aspartate) toward *α*_v_ *β*_3_ integrins was previously used to develop functional materials for tumor imaging, drug delivery and cancer therapy.^[^ ^22^ ^]^ We therefore judiciously devised an activity‐based sensing probe, NBSe‐cRGD, armed with cRGD as an anchor (**Figure** **3**a). We envisioned that this probe can not only detect elevated GSH levels in cancer cells, but also eradicate them through PDT.

As illustrated in Figure 3b, NBSe‐cRGD promptly exhibited a robust and time‐dependent fluorescence activation upon reaction with GSH, with emission maxima at 550 nm (indicating generation of thiol‐NBSe). Furthermore, kinetic studies unveiled that the fluorescence activation was contingent upon GSH concentration. Specifically, the stabilization time decreased as the GSH concentration increased: 20 min for 1 mm GSH, 60 min for 100 µm GSH, and over 1 h for 30 µm GSH (Figure 3c). To investigate the ability of NBSe‐cRGD to selectively image GSH in living cells, we carried out confocal cell imaging experiments. HeLa cells incubated with NBSe‐cRGD exhibited bright green fluorescence (Figure 3e). In contrast, the control group, where cells were incubated with NEM and then treated with NBSe‐cRGD, displayed negligible fluorescence (Figure S38, Supporting Information). In addition, normal NIH3T3 cells exhibited only faint fluorescence upon treatment with NBSe‐cRGD (Figure 3e,f). Furthermore, the GSH‐activatable PDT of NBSe‐cRGD was investigated using ABDA (9,10‐anthracenediylbis‐(methylene)‐dimalonic acid) as a ^1^O_2_ indicator (Figure 3d); the absorbance of ABDA decreased rapidly with increasing irradiation time in the presence of NBSe‐cRGD and GSH, indicating singlet oxygen (^1^O_2_) generation. In the absence of NBSe‐cRGD or GSH, a negligible decrease in the absorbance of ABDA was observed. We also found obvious superoxide radical (O_2_^−•^) and hydroxyl radical (OH•) generation (Figures S39 and S40, Supporting Information), revealing that GSH‐activatable NBSe‐cRGD can also undergo the type 1 mechanism. These results collectively demonstrated that NBSe‐cRGD can be used as an effective GSH‐activated PDT agent.

We further evaluated the potential of NBSe‐cRGD as a GSH‐activated PDT agent in live cells. HeLa cells were first incubated with different concentrations of NBSe‐cRGD and then irradiated with an LED light for 10 min (Figure 3g/h); cell viability assay showed that cancer cells were efficiently killed under LED irradiation, whereas the control group without light exposure showed high cell viability. Moreover, substantial differences in cell viability were observed when one compared NBSe‐cRGD‐pretreated (10 µm) normal NIH3T3 and HeLa cells under irradiation – 75% viability for NIH3T3 cells and less than 30% viability for HeLa cells. Flow cytometry analysis using fluorescently labeled annexin V and propidium iodide (PI) indicated that NBSe‐cRGD primarily triggered cell death through the apoptotic pathway (Figure 3i‐k; Figure S41, Supporting Information).^[^ ^23^ ^]^ These results provide compelling evidence that NBSe‐cRGD served as GSH‐activated phototheragnostic for the precise diagnosis and PDT of cancer cells.

### 2.3 Reactivity Study of Alkoxy‐NBSe Toward Lysine and for HDAC Activity Detection

Our investigations indicated that NBSe‐OMe exhibited lower reactivity toward thiols than NBD‐OMe. This observation prompted us to speculate that NBSe‐OMe might display high chemoselectivity toward lysines, offering a new solution to the selectivity limitation observed with NBD‐OMe. As shown in Figure S42b (Supporting Information), NBSe‐OMe displayed no reactivity toward biologically relevant species such as GSH and BSA. Furthermore, in HeLa cell lysates, NBSe‐OMe displayed no protein reactivity, as confirmed by in‐gel labeling analysis (Figure S42c, Supporting Information). In contrast, NBD‐OMe reacted with GSH and BSA and labelled various proteins in HeLa cell lysates (Figure S34a/c, Supporting Information). Furthermore, the reaction kinetics revealed that the rate of reaction between NBSe‐OMe and *n*‐butylamine was markedly slower than that between NBD‐OMe and *n*‐butylamine (Figure S43, Supporting Information). Moreover, DFT calculations of the transition states involved in these reactions indicated the change from NBD‐OMe to NBSe‐OMe as reactants resulted in an apparent decrease in Gibbs free energy from 22.8 to 20.4 kcal mol^−1^ (Figure S44a, Supporting Information), resulting in a striking 58‐fold reduction in reaction rate.^[^ ^24^ ^]^ In addition, this slower reaction rate can be alleviated by leveraging intramolecular or proximity reactions (Figure S44b, Supporting Information).

**Figure 4:**
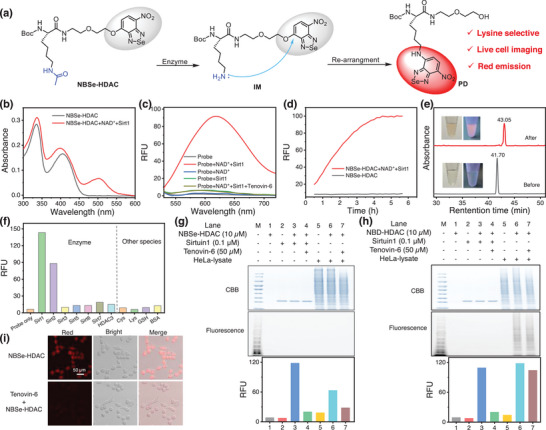
a) Response mechanism of NBSe‐HDAC toward HDACs. b,c) Absorption (b) and fluorescence (c) response of NBSe‐HDAC (10 µm) toward Sirt1 (0.1 µm) within 3 h in HEPES (pH 8.0) under different conditions (NAD^+^: 100 µm; Tenovin‐6: 50 µm). d) Fluorescence kinetic study of NBSe‐HDAC (10 µm) toward Sirt1 (0.1 µm) in HEPES (containing 100 µm NAD^+^). e) Representative HPLC analysis of the enzymatic reaction of NBSe‐HDAC (30 µm) with Sirt1 (0.5 µm) overnight in HEPES (containing 0.5 mm NAD^+^) (detection wavelength: 330 nm). Insets: photographs of colorimetric and fluorescence changes of NBSe‐HDAC solution before and after reacting with Sirt1. f) Fluorescence responses of NBSe‐HDAC (10 µm) toward various HDACs (enzyme concentration: 0.1 µm) and other biological analytes (Cys: 100 µm; Lys: 100 µm; GSH: 1 mm; BSA: 1 µm), respectively in HEPES (*λ*_ex_ = 500 nm, *λ*_em_ = 600 nm). g,h) In‐gel analysis and fluorescence intensity of NBSe‐HDAC (g) and NBD‐HDAC (h) incubated with Sirt1/HeLa lysate in HEPES for 3 h. i) Live‐cell imaging of NBSe‐HDAC in HeLa cells. (Top): cells incubated with NBSe‐HDAC (10 µm) for 3 h. (Bottom): cells were pre‐treated with inhibitor Tenovin‐6 (100 µm) for 40 min, and then incubated with NBSe‐HDAC (10 µm) for 3 h (*λ*_ex_ = 488 nm, *λ*_em_ = 590–650 nm).

Sirtuins (Sirt1‐7) are NAD^+^‐dependent histone deacetylase enzymes involved in the regulation of various lysine PTMs and have emerged as attractive therapeutic targets.^[^ ^25^ ^]^ Inspired by previous investigations,^[^ ^15^, ^16^, ^17^ ^]^ we designed NBSe‐HDAC, an alkoxy‐NBSe‐based probe, with the goal of detecting the enzymatic activity of various sirtuins by using intramolecular/ proximity reactions (**Figure** **4**a). Our probe comprises a Kac recognition group and a fluorogenic moiety alkoxy‐NBSe, connected by a flexible aminoethoxy linker to facilitate the efficient intramolecular reaction. We hypothesized that the enzymatic deacetylation reaction of NBSe‐HDAC releases the amino group to form the intermediate IM, which subsequently undergoes an intramolecular substitution reaction to produce a highly emissive product PD.

After synthesizing NBSe‐HDAC, we examined its absorption and fluorescence response in the presence of Sirt1. NBSe‐HDAC itself exhibited two absorption peaks at 330 and 410 nm, but was non‐fluorescent. Upon incubation with Sirt1 and NAD^+^ in HEPES buffer, a new absorption peak appeared at 510 nm (Figure 4b), accompanied by a strong emission peak appeared at 620 nm (Figure 4c). These absorption and fluorescence spectra of the mixture of NBSe‐HDAC with Sirt1 and NAD^+^ were characteristic of NBSe‐NHR, indicating the occurrence of the intermolecular reaction between lysine and alkoxy‐NBSe. In contrast, control groups with Sirt 1 or NAD^+^ alone, or in the presence of inhibitor (Tenovin‐6) displayed negligible fluorescence. These results offered solid evidence for the probe's efficacy in detecting sirtuin activity. We next performed enzymatic reaction kinetic studies (Figure 4d); the solution of NBSe‐HDAC showed very little fluorescence over 6 h (black line), implying its excellent stability in buffer conditions. Upon addition of Sirt1 and NAD^+^, its fluorescence increased rapidly, reaching a plateau after 4 h (red line). According to modified Michaelis–Menten equations,^[^ ^26^ ^]^ the turnover number (*k*_cat_) and Michaelis constant (*K*_m_) of Sirt 1 toward probe NBSe‐HDAC were calculated to be 0.48 s^−1^ and 161 µm using the fluorescence method (Figure S51, Supporting Information). LC‐MS analysis revealed the appearance of a new peak with a retention time of 43 min at both 330 and 500 nm channels following Sirt1 treatment, with MS confirmation (561.6) to be that of NBSe‐NHR (Figure 4e; Figures S47–S49, Supporting Information).

We next tested the selectivity of NBSe‐HDAC toward various enzymes, namely Sirt2, Sirt3, Sirt5, Sirt6, Sirt7, and HDAC3 (Figure 4f); Sirt1 and Sirt2 induced remarkable fluorescence enhancement when compared to other enzymes, thus corroborating with previous findings.^[^ ^15^ ^]^ Moreover, we examined the fluorescence responses of NBSe‐HDAC in the presence of various interfering species, including BSA, GSH, Cys, Lys; none of these species resulted in a fluorescence change, indicating the excellent selectivity of the probe (Figures S52 and S53, Supporting Information). In stark contrast, a previously reported probe (NBD‐HDAC^[^ ^15^ ^]^) was strongly interfered by other species (e.g., biothiols and BSA) (Figure S54, Supporting Information). Furthermore, we investigated the capability of NBSe‐HDAC to assess the enzymatic activity of HDAC in cellular lysates by incubating the probe with HeLa lysates followed by in‐gel fluorescence scanning (Figure 4g); incubation of the probe with HeLa lysates led to significant increase in fluorescence, which was suppressed with Tenovin‐6‐treated with HeLa lysates, indicating that the fluorescence turn‐on was mainly attributed to enzymatic activity of HDAC in lysates. In addition, no in‐gel fluorescent bands were observed when HeLa lysates were incubated with the probe, indicating that the probe was not interfered by a large number of endogenous proteins. These results collectively proved that our NBSe probe can detect HDAC activity in lysates with high selectivity. As a comparison, we also investigated the sensing performance of NBD‐HDAC with cellular lysates (Figure 4h); incubation of NBD‐HDAC with HeLa lysates did induce a significant fluorescence increase. However, the fluorescence of cell lysates remained almost unchanged after Tenovin‐6 treatment. Furthermore, in‐gel fluorescence labeling revealed that a large number of proteins were labeled by NBD‐HDAC. These results indicate the poor selectivity of NBD‐HDAC, which can react with endogenous proteins in cellular lysates.

The establishment of superior selectivity of NBSe‐HDAC when compared to previous HDAC probes^[^ ^15^ ^]^ prompted us to conduct live‐cell imaging in HeLa cells expressing sirtuins with confocal fluorescence microscopy (Figure 4i); results demonstrated the presence of HDAC‐dependent bright red fluorescence. These experiments thus yielded convincing evidence that NBSe‐HDAC is an effective tool for visualizing HDAC activity in live cells. It is worth noting that very few small‐molecule probes are available for live‐cell detection of HDAC enzymatic activities.

### 2.4 Proximity‐Driven Protein Modification and Activatable PDT Therapy for Cancer Treatment

**Figure 5:**
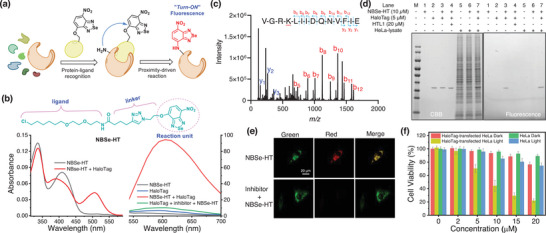
a) Proximity‐driven labelling mechanism of target protein by alkoxy‐NBSe probe. b) Chemical structure of NBSe‐HT. Absorption and fluorescence response of NBSe‐HT (10 µm) toward HaloTag (5 µm) under various conditions. c) MS/MS analysis of the NBSe‐modified peptide. d) In‐gel analysis of NBSe‐HT responding to HaloTag in HEPES (pH 8.0) containing 2% DMSO at 37 °C for 3 h. e) Fluorescence images of HeLa cells transfected with HaloTag‐GFP‐Mito under a confocal microscope (top row: cells incubated with NBSe‐HT (10 µm) for 3 h at 37 °C; bottom row: cells pre‐treated with competitor HTL1 (100 µm) for 1 h, and then incubated with NBSe‐HT (10 µm) for 3 h; *λ*_ex_ = 488 nm, green channel: 500–530 nm; red channel: 590–650 nm). f) Phototoxicity of NBSe‐HT toward HeLa cells transfected with or without HaloTag‐GFP‐Mito under dark and LED irradiation (490–500 nm, 30 mW cm^−2^, 10 min) (incubation time: 3 h).

Proximity‐driven modification has been proven to be a powerful strategy for site‐specific protein labelling, enabling visualization of target protein interactions, dynamics, and subcellular localization.^[^ ^27^ ^]^ As shown in **Figure** **5**a, the interaction of targed protein and the ligand tethered to a suitable warhead drives the warhead to selectively react with a nucleophilic amino acid proximal to the protein binding‐site. The effectiveness of the chemistry between the warhead and the amino acid is influenced by proximity effect made possible by ligand‐protein recognition. To assess the applicability of our NBSe platform for chemoselective protein modification, we designed and synthesized NBSe‐HT, which consists of a HaloTag ligand and an alkoxy‐NBSe unit, for precise labeling of HaloTag fusion proteins and activatable phototherapy (Figure 5b).

The probe exhibited absorption maximum at 505 nm and emission maximum at 600 nm upon HaloTag addition, consistent with the characteristic absorption and emission peaks of NBSe‐NHR (Figure 5b). NEM‐pretreated HaloTag protein (thiol blocked) exhibited a similar increase in fluorescence signal upon addition of NBSe‐HT when compared to the untreated protein (Figure S55, Supporting Information). This observation indicates that our probe selectively reacted with lysine rather than cysteine. Docking analysis revealed that the reaction unit of NBSe‐HT was readily accessible to K^160^ in HaloTag7 (Figure S56, Supporting Information). Furthermore, enzymatic digestion and LC‐MS/MS analysis of the reaction product indicated that the modification mainly occurred in the peptide VGRK^160^LIIDQNVFIE, confirming the regioselectivity of this reaction (Figure 5c). Subsequent selectivity investigations substantiated the remarkable specificity of NBSe‐HT toward HaloTag, as it exhibited no reactivity with other biologically relevant species (Figure S57, Supporting Information). In‐gel labeling experiments proved that NBSe‐HT selectively labeled HaloTag, even in the presence of large amounts of other proteins (lane 7 in Figure 5d). Notably, treatment of an excessive competitor (HTL1) effectively abrogated the labeling of HaloTag by NBSe‐HT (lane 4). These results highlight the capability of NBSe‐HT to achieve precise labeling of HaloTag, primarily driven by protein‐ligand interactions.

Encouraged by the excellent performance of NBSe‐HT in solution, we hypothesized that NBSe‐HT could selectively bind to HaloTag‐fusion proteins in live cells, and induce fluorescence activation. To test this hypothesis, we incubated HaloTag‐GFP‐Mito transfected HeLa cells with NBSe‐HT and monitored the fluorescence change using a confocal microscope. A gradual increase in fluorescence signals could be observed in the red channel (Figure S58, Supporting Information), which became significant after 3‐h incubation (Figure 5e). Importantly, pre‐incubation of cells with the competing reagent HTL1 effectively prevented fluorescence activation (Figure 5e). These experiments proved the effectiveness of the “turn‐on” fluorescent labeling strategy using HaloTag proteins in live cells.

We next explored the potential of NBSe‐HT as a photosensitizer activated by HaloTag proteins. As illustrated in Figure S59 (Supporting Information), either NBSe‐HT alone or Halotag alone caused minimal absorption change of ABDA under light irradiation, but treating NBSe‐HT with HaloTag led to a significant decrease in ABDA absorbance, indicating interaction between HaloTag and NBSe‐HT induced the generation of NBSe‐NHR (a highly efficient ^1^O_2_ generator). Subsequently, HeLa cells were transfected with HaloTag‐GFP‐Mito to evaluate the cytotoxicity of NBSe‐HT under dar/light conditions. In the absence of light, NBSe‐HT exhibited low cytotoxicity toward transfected HeLa cells. However, upon exposure to LED light, it exhibited potent phototoxic effects, resulting in 70% cell death (20 µm; Figure 5f). These findings demonstrate the successful activation of NBSe‐HT phototoxicity in live cells by HaloTag proteins. Importantly, this approach can be expanded to incorporate diverse cancer biomarker ligands into our NBSe platform, thereby broadening the repertoire of tools for fluorescence‐guided diagnostics and phototherapy.

## 3 Conclusion

In summary, we have demonstrated that NBSe derivatives possess unique reactivity and can be rationally developed as highly efficient fluorescent warheads for selective labeling of cysteines or lysines under biocompatible conditions. Chloro or phenoxy‐substituted NBSe derivatives exhibit high specificity toward cysteines in proteome labeling, providing new opportunities for chemical proteomic profiling and covalent‐drug design. On the other hand, alkoxy‐NBSe, a latent electrophile, can be designed to selectively react with lysines in a protein in regioselective manner using proximity‐driven strategy. In addition, NBSe derivatives exhibit unique photophysical properties, and can serve as imaging fluorophores as well as small‐sized photosensitizers. Although the current probes have not yet been effective for in vivo PDT experiments, we can further expand the conjugation system to shift absorption to longer wavelengths or extend the strategy to design activatable PDT triggered by cancer‐overexpressed proteins. We envision that our study will provide new insight into designing selective warheads for proteinogenic amino acid modification and add useful tools for the development of protein‐based therapeutics.

## Conflict of Interest

The authors declare no conflict of interest.

## Supporting information

Supporting Information

## Data Availability

The data that support the findings of this study are available in the supplementary material of this article.
