# Supplementary material for: Reactivity‐Tunable Fluorescent Platform for Selective and Biocompatible Modification of Cysteine or Lysine
Source: Adv Sci (Weinh). 2024 Jun 19;11(31):2402838. doi: 10.1002/advs.202402838 (PMC11336953; doi:10.1002/advs.202402838)
Supplement: Supplementary file 1 — Supporting Information [file ADVS-11-2402838-s001.pdf]

ADVANCED SCIENCE
Open Access

Supporting Information

for *Adv. Sci.*, DOI 10.1002/advs.202402838

Reactivity-Tunable Fluorescent Platform for Selective and Biocompatible Modification of Cysteine or Lysine

Xiaojie Ren, Haokun Li, Hui Peng, Yang Yang, Hang Su, Chen Huang, Xuan Wang, Jie Zhang, Zhiyang Liu, Wenyu Wei, Ke Cheng, Tianyang Zhu, Zhenpin Lu, Zhengqiu Li, Qian Zhao, Ben Zhong Tang, Shao Q. Yao, Xiangzhi Song* and Hongyan Sun**

Supporting Information

Reactivity-Tunable Fluorescent Platform for Selective and Biocompatible Modification of Cysteine or Lysine

Xiaojie Ren,[a, b] Haokun Li,[a] Hui Peng,[c] Yang Yang,[d] Hang Su,[b] Chen Huang,[a] Xuan Wang,[f, g] Jie Zhang,[a] Zhiyang Liu,[a] Wenyu Wei,[a] Ke Cheng,[a] Tianyang Zhu,[d] Zhenpin Lu,[a] Zhengqiu Li,[c] Qian Zhao,[d] Benzhong Tang,[e] Shao Q. Yao,[f]* Xiangzhi Song,[b]* and Hongyan Sun[a]*

[a] Department of Chemistry and Centre of Super-Diamond and Advanced Films (COSDAF), City University of Hong Kong, 83 Tat Chee Avenue, Kowloon, Hong Kong, China, 999077.

[b] College of Chemistry & Chemical Engineering, Central South University, Changsha, Hunan Province, China, 410083.

[c] International Cooperative Laboratory of Traditional Chinese Medicine Modernization and Innovative Drug Development (MOE), MOE Key Laboratory of Tumor Molecular Biology, School of Pharmacy, Jinan University, Guangzhou, Guangdong Province, China, 510632.

[d] Department of Applied Biology and Chemical Technology, The Hong Kong Polytechnic University, Hung Hom, Kowloon, Hong Kong, China, 999077.

[e] Department of Chemistry, Hong Kong University of Science and Technology, Clear Water Bay, Kowloon, Hong Kong, China, 999077.

[f] Department of Chemistry, National University of Singapore, Singapore, 117543 (Singapore).

[g] School of Pharmaceutical Sciences (Shenzhen), Shenzhen Campus of Sun Yat-sen University, Shenzhen, China, 518107.

* Corresponding authors: chmyaosq@nus.edu.sg (Shao Q. Yao); xzsong@csu.edu.cn (Xiangzhi Song); hongysun@cityu.edu.hk (Hongyan Sun).

Contents

1. **Chemical structures of investigated compounds S3**
2. **General methods S4**
3. **Photophysical properties of NBX-derivatives S7**
4. **Theoretical calculations S10**
5. **Supplementary data for small-molecule thiols profiling S15**
6. **Supplementary data for protein thiols profiling S20**
7. **Supplementary data for GSH-activated PDT S33**
8. **Supplementary data for reactivity study of alkoxy-NBSe toward Lysine S36**
9. **Supplementary data for HDAC detection S40**
10. **Supplementary data for HaloTag detection S45**
11. **Chemical synthesis S48**
12. **NMR and HRMS spectra S57**
13. **Reference S86**

1. Chemical structures of investigated compounds

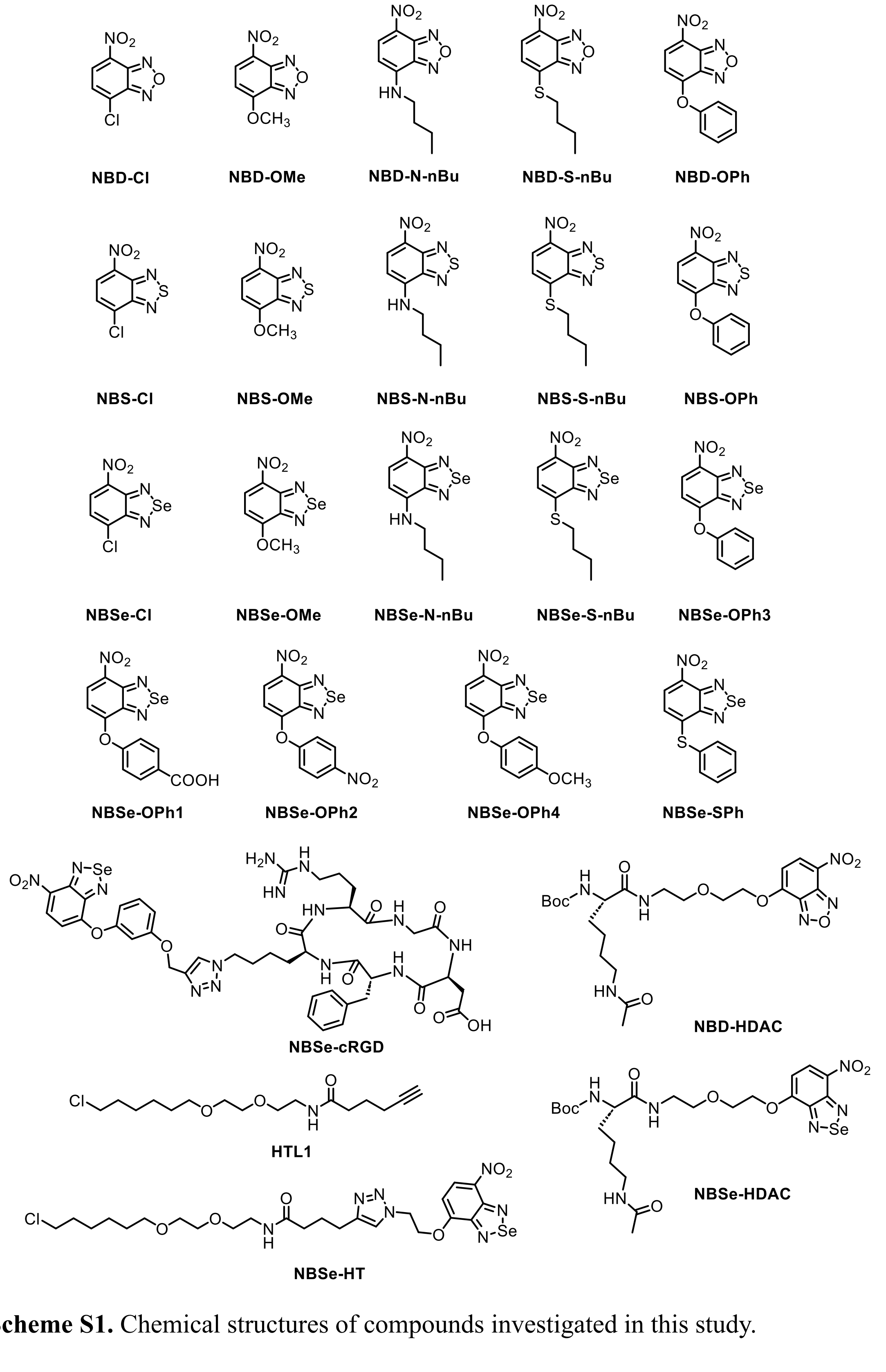

Scheme S1. Chemical structures of compounds investigated in this study.

2. General methods

2.1 Materials and instruments

Unless otherwise stated, all reagents from commercial suppliers were used without further purification. Twice-distilled water was used throughout all experiments. Cell culture related items, including fetal bovine serum (FBS), Dulbecco's modified Eagle's medium (DMEM), trypsin-EDTA, PBS, and penicillin/streptomycin, were purchased from Invitrogen. DCFH-DA, calcein AM and propidium iodide (PI) were purchased from Abcam. 96-well of ibidi® culture plates were purchased from ibidi GmbH.

Chemical reaction progress was monitored by TLC on silica plates, and spots were visualized by UV, iodine, or other suitable stains. Column chromatography was performed using silica gel (mesh 200-300), which was obtained from Qingdao Ocean Chemicals, China. NMR spectra were recorded on a BRUKER 400 MHz spectrometer, using TMS as an internal standard. Mass spectrometric experiments were performed on a micro TOF-Q II mass spectrometer (Bruker Daltonik, Germany) and a SCIEX LC-MS system (API-3200). HPLC analysis was performed on the 1525 Waters HPLC system equipped with a 2489 UV/Visible detector. Water containing 0.1% trifluoroacetic acid (TFA) and acetonitrile containing 0.1% TFA were used as eluents, and the flow rate was set at 1 mL/min. Absorption and emission spectra were recorded on Molecular Devices SpectraMax ID5 Microplate Reader. Fluorescence imaging experiments were carried out with a Leica TCS SP5 confocal scanning microscope.

2.2 Detection of fluorescent quantum yields

The fluorescent quantum yields were measured using a standard reference and calculated from the following equation: $\Phi_s = \Phi_r \cdot \frac{F_s}{F_r} \cdot \frac{A_r}{A_s} \cdot \frac{n_s^2}{n_r^2}$. (Eq-1)

Φ denotes the fluorescent quantum yield; F means the integral intensity of fluorescence; A refers to the absorbance at the excitation wavelength; and *n* is the refraction index of solvent. *s* and *r* represent the testing sample and the reference, respectively.

2.3 Computation Details

All calculations were carried out using ORCA (version 5.0.3).[1] Geometry optimization was performed at the density functional theory (DFT) using the hybrid functional B3LYP with the Becke-Johnson damping scheme atom-pairwise dispersion correction (D3BJ) with def2-TZVP(-f) basis set.[2] Water as the solvent of the solution was modelled by the Conductor-like Polarizable Continuum Model (CPCM) in optimization. Harmonic vibrational analyses were carried out to confirm whether the optimized structure is a local minimum structure or a first order transition state and to provide zero-point vibrational energy corrections and thermal corrections to various thermodynamic properties. (Relaxed) Potential energy surface scans

were carried out to confirm the existence of transition states. Single-point energies were carried out at PWPB95-D3BJ/ma-def2-TZVPP level of theory with the SMD solvation model to model the water solvent. RIJCOSX was turned on for all calculations and def2/J, def2-TZVPP/C or 'autoaux' aux basis sets were used in corresponding calculations. ORCA default grid settings were used and 'Tightopt' and 'TightSCF' settings were set in corresponding calculations. The Multiwfn package[3] and the VMD package were used to depict the electrostatic potential.

2.4 Determination of $^1O_2$ generation

9,10-Anthracenediylbis-(methylene)-dimalonic acid (ABDA) was used as $^1O_2$ indicator. The absorbance at 380 nm of ABDA in water was adjusted to about 1.0. To 1 mL of ABDA solution was added 10 μM of the respective probe. The resultant mixture was exposed to a light irradiation for a certain time and its absorption spectra were recorded after each irradiation. LED light sources with a power of 30 mW/cm^2 and different wavelengths were used: 490-500 nm for **NBSe-HDAC** and **NBSe-HT**; 400-800 nm for **NBSe-cRGD**.

2.5 Determination of $O_2^{-\bullet}$ generation

Dihydrorhodamine 123 (DHR123) was used to evaluate the $O_2^{-\bullet}$ generation efficiency.[4] DHR123 can be converted to Rhodamine 123 in the presence of $O_2^{-\bullet}$ and emit strong green fluorescence at 525 nm. Both probe **NBSe-cRGD** and DHR123 were prepared as 10 μM in water. The resultant mixture was exposed to a LED light (400-800 nm, 30 mW/cm^2) irradiation for a certain time and the emission spectra were recorded after each irradiation (Ex: 460 nm).

2.6 Determination of OH• generation

3'-(4-hydroxyphenyl) fluorescein (HPF) was used as the hydroxyl radical (OH•) indicator.[4] Briefly, both **NBSe-cRGD** and HPF were prepared as 10 μM in aqueous solution. Then, the resultant mixture was exposed to a LED light (400-800 nm, 30 mW/cm^2) irradiation for a certain time and its emission spectra were recorded after each irradiation (Ex: 460 nm).

2.7 Flow Cytometry of Apoptosis

The Annexin V-FITC/PI apoptosis kit (obtained from Beyotime Biotech. Inc) was used in cell apoptosis to evaluate the phototoxicity of probe **NBSe-cRGD**. HeLa cells were seeded in 6-well plates at a density of 6 × 10^5 cells per well and incubated at 37 °C overnight, followed by refreshing the culture media with 2 mL complete media. Next, **NBSe-cRGD**-treated cells were divided into two groups: the first group were directly cultured for 24 h; and the second group were irradiated with a LED light for a certain time and further cultured for 24 h. Afterwards, the culture media were removed, and cells were collected and stained using an Annexin V-FITC/PI apoptosis kit according to the protocol. The cell apoptosis was then determined by flow cytometry (NAVIOS Beckman Coulter, USA).

2.8 Cell lysate preparation

For in vitro proteome labeling, cell lysates were first prepared from HeLa cells. Generally, HeLa cells were cultured to 90% confluence. The medium was removed, and cells were washed twice with cold PBS. RIPA lysis buffer (Pierce) with protease inhibitors (Halt) was added, and cells were scraped into lysis buffer. The solution was placed at 0 °C for 30 min and then centrifuged to obtain the supernatant as cell lysate. Protein concentration was determined by the BCA protein assay kit (Pierce) and adjusted to 1 mg/mL with PBS or HEPES buffer.

2.9 In-gel analysis

Probes were incubated with HeLa lysate in PBS or HEPES buffer at a concentration of 1 mg/mL. Subsequently, prechilled acetone was added to the above solution to precipitate the protein. The protein pellet was collected by centrifugation at 4 °C for 2 min, dissolved in 1× SDS loading buffer, and then subjected to SDS-PAGE analysis. The gel was visualized using in-gel fluorescence scanning and Coomassie brilliant blue (CBB) staining.

2.10 Cell transfection with HaloTag7

For the transfection experiments, HeLa cells were seeded into a confocal dish and cultured to 20%−30% cell confluence. The medium was replaced with DMEM containing 10% FBS. Cells were then transfected using Lipofectamine 3000 (Invitrogen, USA) with plasmid HaloTag-GFP-Mito (a gift from Michael Lampson, Addgene plasmid #67762). Specifically, 1 μg of plasmid and 2 μL of P3000 were gently added into 75 μL of Opti-MEM medium. 3 μL of Lipo3000 was gently added into 75 μL of Opti-MEM medium. The two mixtures were gently mixed in a 1:1 ratio and the resulting solution was incubated for 15 min. The obtained transfection mixture was added into the cell culture dish, and the cells were further incubated for 6−8 h at 37 °C to facilitate transfection. After changing the medium, the cells were cultured for another 30 h to express the HaloTag fusion protein.

3. Photophysical properties of NBX-derivatives

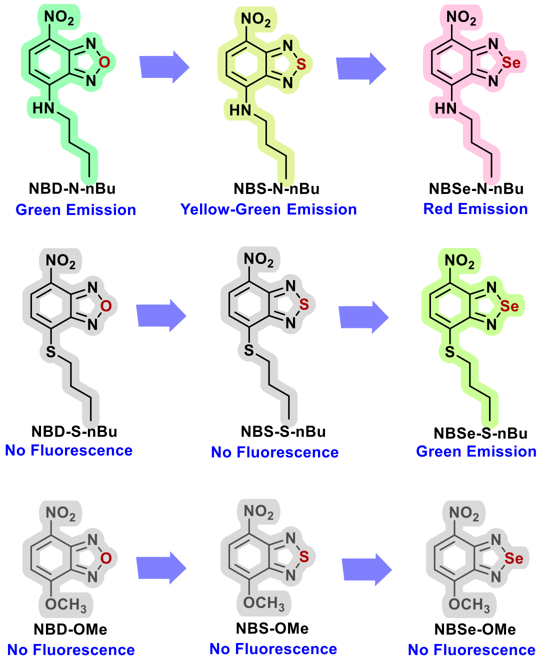

Scheme S2. Chemical structures and fluorescence properties of NBX derivatives.

Table S1. Photophysical properties of NBD-derivatives in the mixture of DMSO.

Dyes	λ_{abs}/nm	λ_{em}/nm	Δ_{ss}/nm	Φ	R. F. I.
**NBD-N-nBu**	474	543	69	0.576	100
**NBD-S-nBu**	424	524	100	0.009	1.5
**NBD-OMe**	379	/	/	< 0.001	0
**NBS-N-nBu**	461	543	82	0.294	51
**NBS-S-nBu**	423	509	86	0.008	1.4
**NBS-OMe**	376	/	/	< 0.001	0
**NBSe-N-nBu**	497	598	101	0.178	31
**NBSe-S-nBu**	440	539	99	0.104	18
**NBSe-OMe**	398	/	/	< 0.001	0

Δ_{ss} represents the Stokes shift; Φ is the fluorescent quantum yield determined using fluorescein as the reference (Φ = 0.92 in 0.1 M NaOH at 25 ℃)[5]; R.F.I represents the relative fluorescence intensity, using **NBD-N-nBu** as the standard.

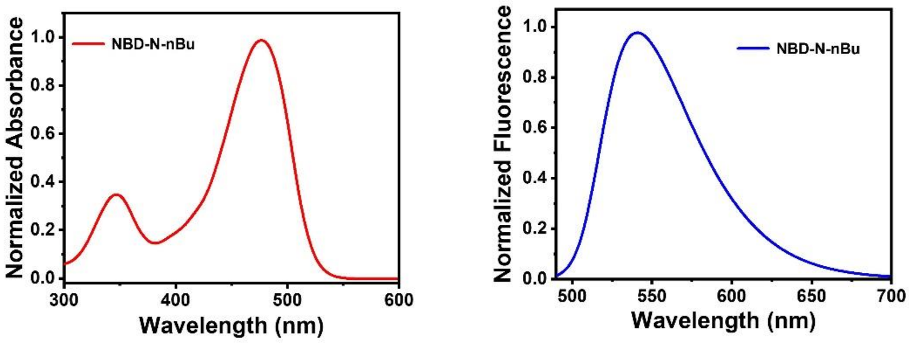

Figure S1 Normalized absorption (left) and fluorescence (right) spectra of **NBD-N-nBu** in DMSO.

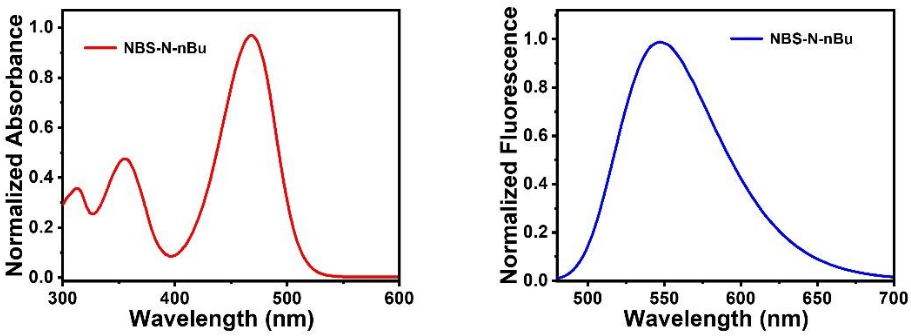

Figure S2 Normalized absorption (left) and fluorescence (right) spectra of **NBS-N-nBu** in DMSO.

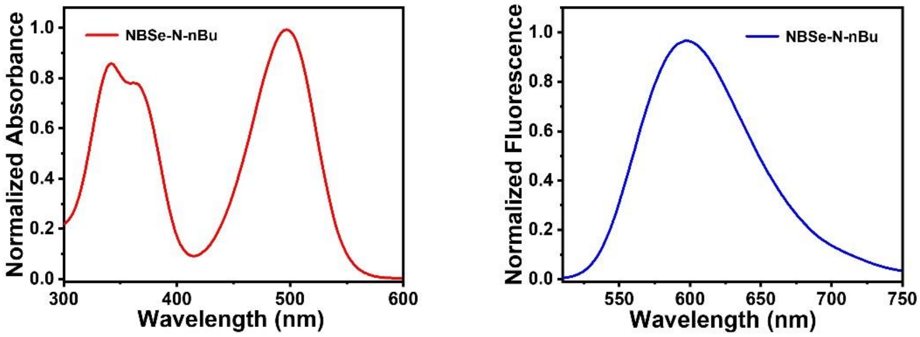

Figure S3 Normalized absorption (left) and fluorescence (right) spectra of **NBSe-N-nBu** in DMSO.

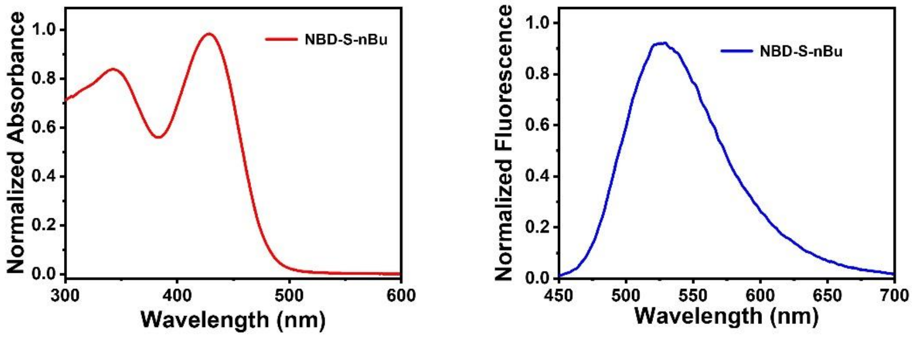

Figure S4 Normalized absorption (left) and fluorescence (right) spectra of **NBD-S-nBu** in DMSO.

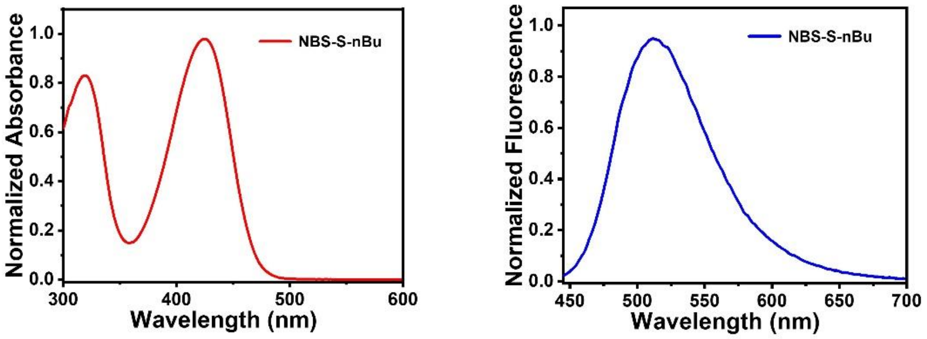

Figure S5 Normalized absorption (left) and fluorescence (right) spectra of **NBS-S-nBu** in DMSO.

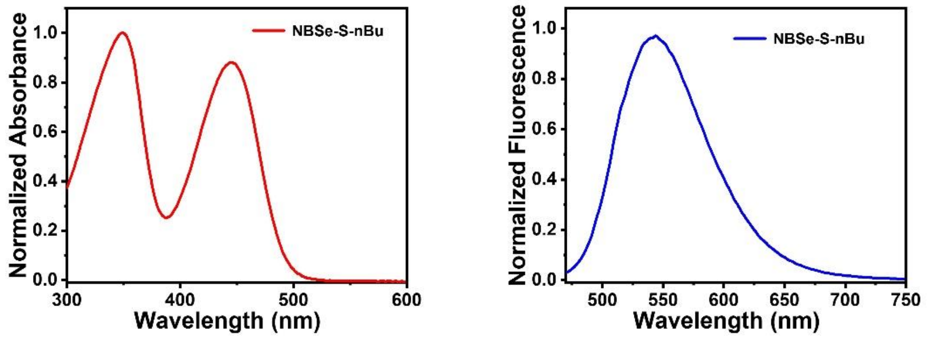

Figure S6 Normalized absorption (left) and fluorescence (right) spectra of **NBSe-S-nBu** in DMSO.

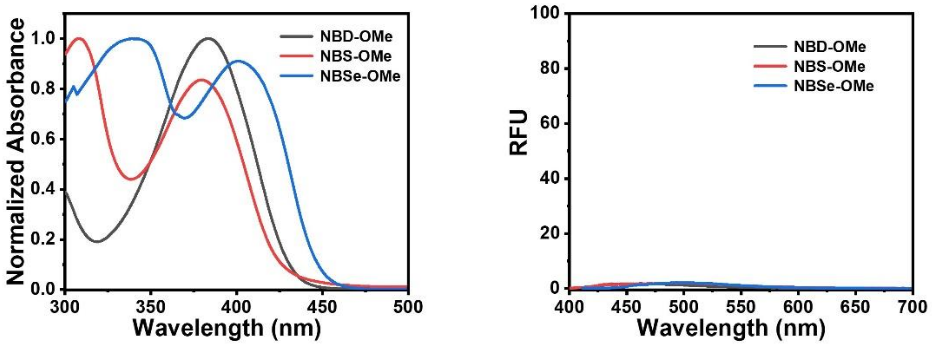

Figure S7 Absorption (left) and fluorescence (right) spectra of **NBD-OMe** (black line), **NBS-OMe** (red line) and **NBSe-OMe** (blue line) in DMSO.

4. Theoretical calculations

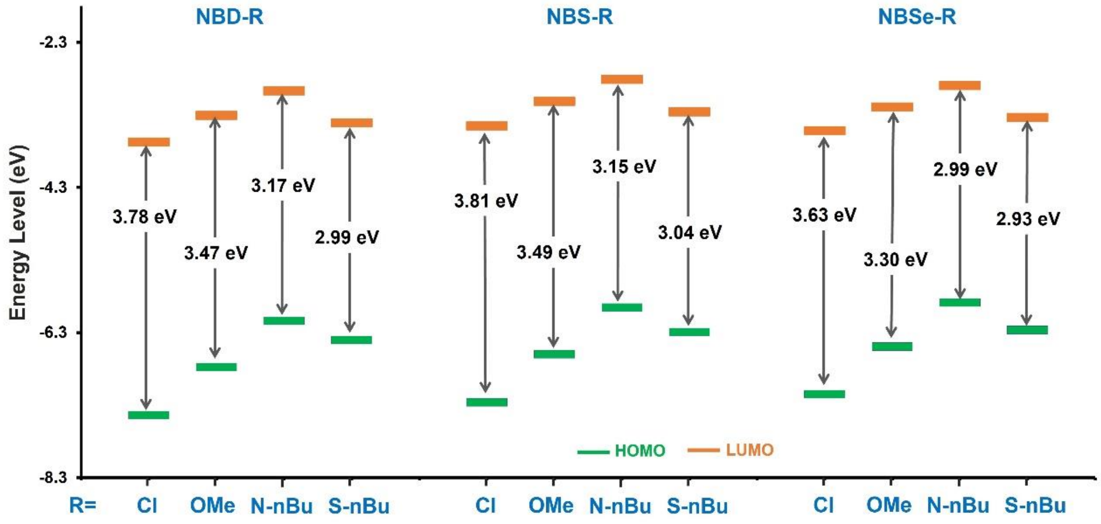

Figure S8 The energy gaps between LUMOs and HOMOs of **NBD-R**, **NBS-R** and **NBSe-R** in water. R denotes the substituted group at 4-position of NBX derivatives.

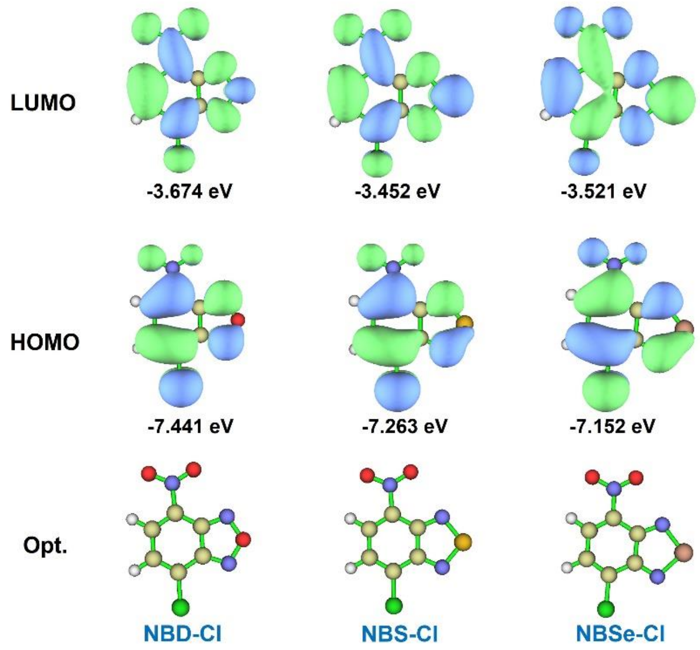

Figure S9 LUMOs (top row) and HOMOs (middle row) orbitals and optimized molecular structures (bottom row) of **NBD-Cl**, **NBS-Cl** and **NBSe-Cl** in water.

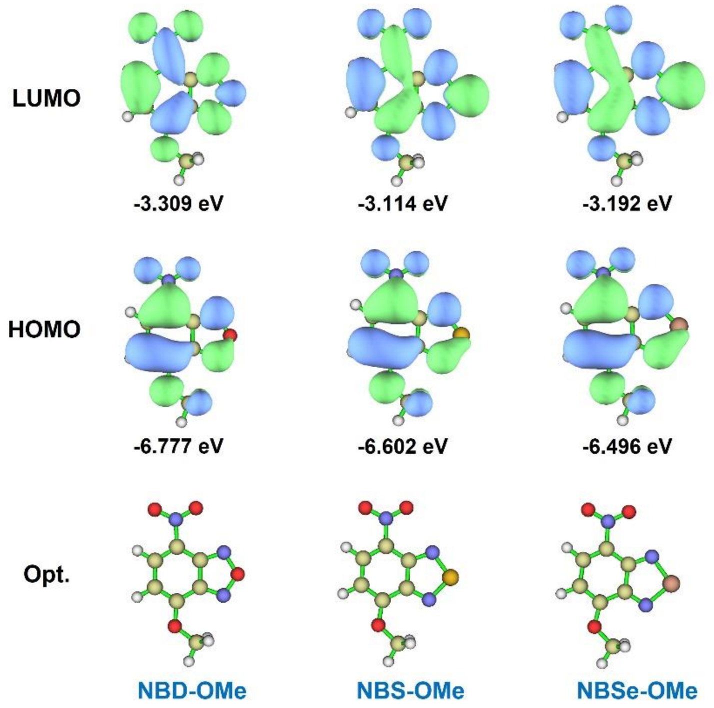

Figure S10 LUMOs (top row) and HOMOs (middle row) orbitals and optimized molecular structures (bottom row) of **NBD-OMe**, **NBS-OMe** and **NBSe-OMe** in water.

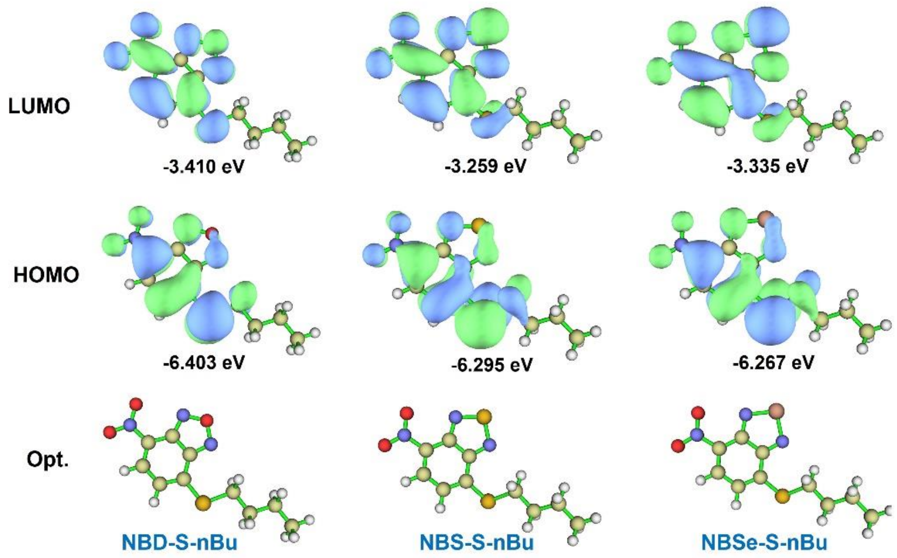

Figure S11 LUMOs (top row) and HOMOs (middle row) orbitals and optimized molecular structures (bottom row) of **NBD-S-nBu**, **NBS-S-nBu** and **NBSe-S-nBu** in water.

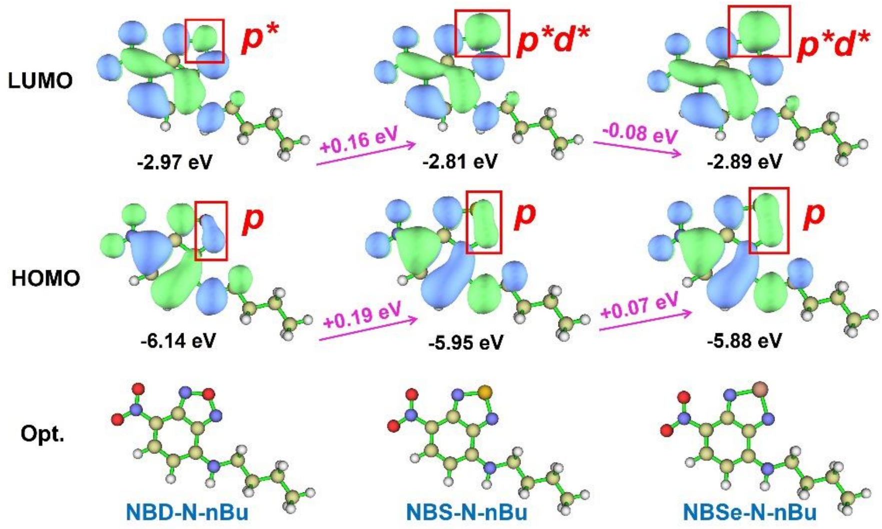

Figure S12 LUMOs (top row) and HOMOs (middle row) orbitals and optimized molecular structures (bottom row) of **NBD-N-nBu**, **NBS-N-nBu** and **NBSe-N-nBu** in water.

Note: NBX fluorophores with an amino donor were taken as the representatives to study the characters of frontier molecular orbitals. As shown in Figure S12, the diazole fragments in these molecules function as electron-withdrawing groups. For HOMO orbitals, O, S, and Se atoms participate π-systems via n-π/*p*-π conjugation by their lone electron pairs, respectively, and the conjugation effect was enhanced in the order of O < S < Se due to their decreased electronegativity. As a result, the HOMO levels of **NBD-N-nBu**, **NBS-N-nBu** and **NBSe-N-nBu** were decreased sequentially. For LUMO orbitals, there are two types of conjugation effects affecting the LUMO energy levels: 1) n*-π* conjugation results in the increase of LUMO level due to the strong electron donating ability of lone pairs; 2) *d*π*-*p*π* conjugation, between the vacant *d** orbital of S[6] /Se[6c-e, 7] atom and adjacent π* orbital, allows the transfer of the adjacent π* electron to vacant *d** orbital resulting in the decrease of the LUMO level. Consequently, the LUMO energy levels of **NBS-N-nBu** and **NBSe-N-nBu** were 0.16 eV and 0.08 eV higher than that of **NBD-N-nBu**, respectively. In addition to the vacant 4*d*-functions, the 3*d* orbitals of Se atom may also contribute to the extension of valence shell and participate in π conjugation.[6e] Thus, the *d*-function makes a more substantial contribution to the description of the π-hyperconjugation in **NBSe-N-nBu** than **NBS-N-nBu**. As a result, the HOMO energy level was increased by 0.26 eV and the LUMO energy level was increased by 0.08 eV from **NBD-N-nBu** to **NBSe-N-nBu**, leading to the remarkable decrease in HOMO-LUMO energy gaps. This result was in good agreement with the fact that **NBSe-N-nBu** displayed a red-shifted absorption compared to **NBD-N-nBu**. From **NBD-N-nBu** to **NBS-N-nBu**, the increases in the HOMO and LUMO energy levels were 0.19 eV and 0.16 eV respectively, and we observed a small red-shift in the absorption spectra.

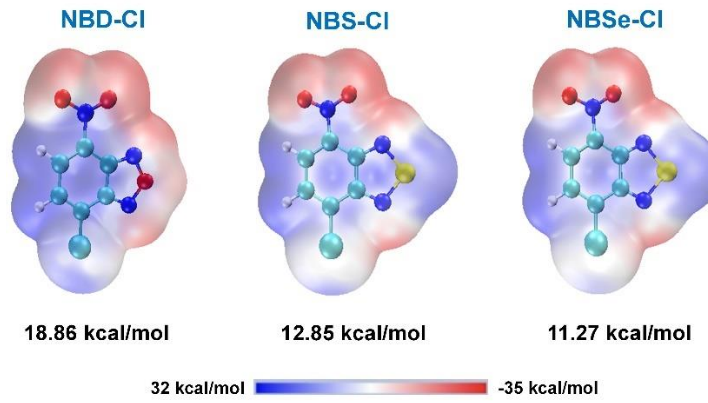

Figure S13 Electrostatic potentials of **NBD-Cl**, **NBS-Cl** and **NBSe-Cl**.

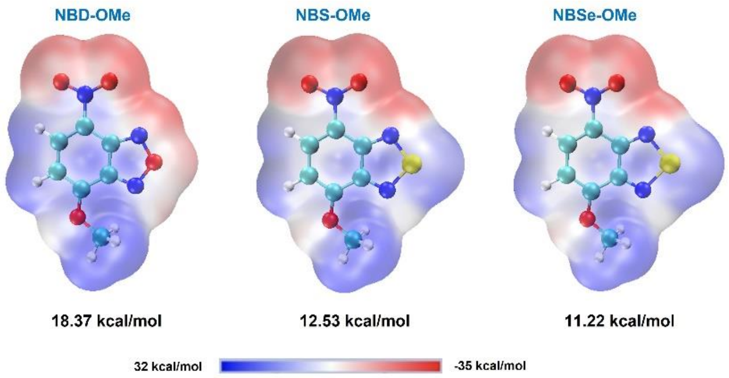

Figure S14 Electrostatic potentials of **NBD-OMe**, **NBS-OMe** and **NBSe-OMe**.

Note: The electrostatic potential calculations were utilized to assess the electrophilic substitution reactions' activity. Figure S13 illustrates the electrostatic potential maps of **NBD-Cl**, **NBS-Cl**, and **NBSe-Cl**. It is noteworthy that the benzene ring in **NBSe-Cl** exhibits a less positive electrostatic potential than **NBD-Cl** and **NBS-Cl** due to the 4*d*-function of Se. The electrostatic potential energy of C4 in **NBSe-Cl** is 11.27 kcal/mol, which is lower than that of **NBS-Cl** (12.85 kcal/mol) and **NBD-Cl** (18.86 kcal/mol). As a result, the reactivity of nucleophilic substitution at the C4 position follows the sequence of **NBSe-Cl** < **NBS-Cl** < **NBD-Cl**. A similar trend was observed for **NBSe-OMe**, **NBS-OMe** and **NBD-OMe** (Figure S14).

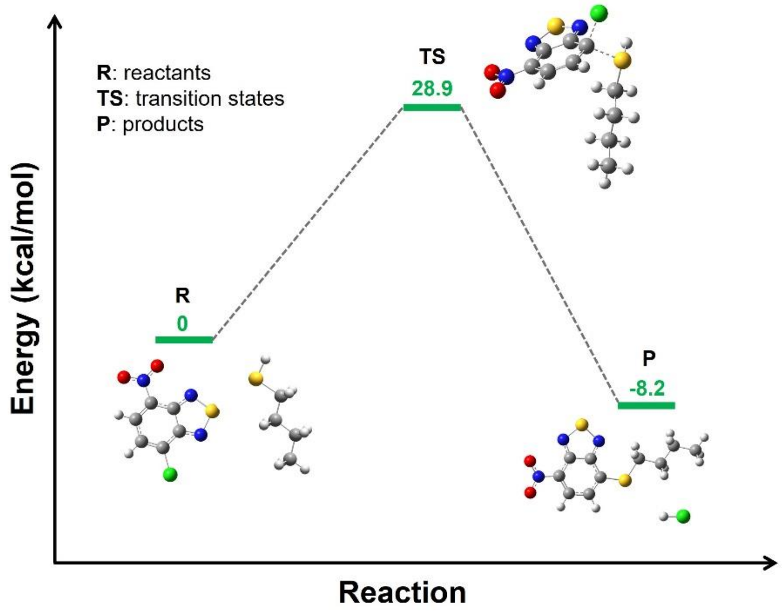

Figure S15 Computed energy profiles and transition states in the reaction of **NBS-Cl** with *n*-butylthiol. Gibbs free energies, kcal/mol.

5. Supplementary data for small-molecule thiols profiling

5.1 Fluorescence responses of NBX-based probes towards Cys

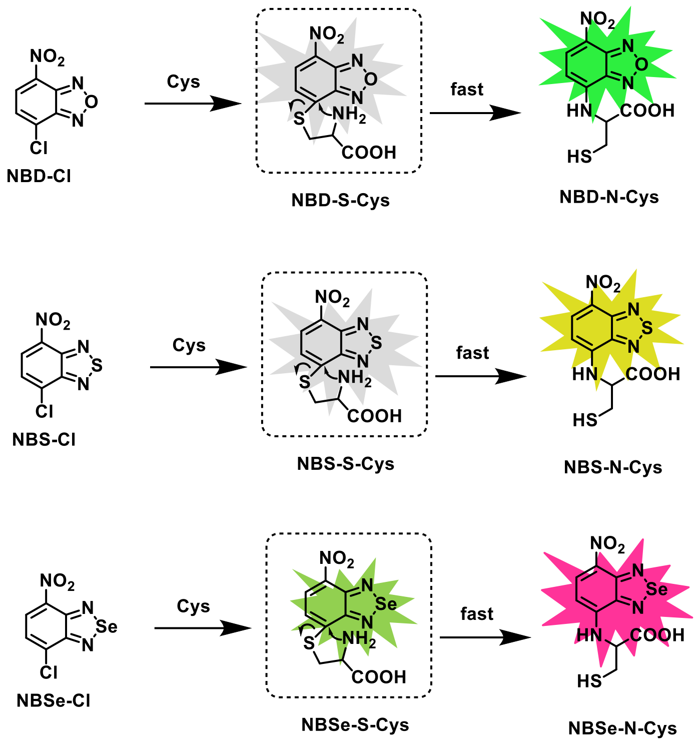

Scheme 3. Proposed sensing mechanism of **NBX-Cl** towards Cys.

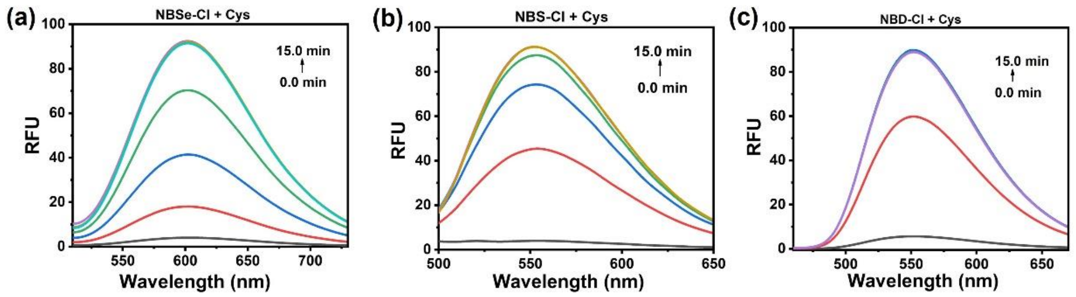

Figure S16 Time-dependent fluorescence responses of **NBSe-Cl/NBS-Cl/NBD-Cl** (10 μM) toward Cys (100 μM) in PBS buffer (pH 7.4, 10 mM, containing 20% DMSO) at room temperature, respectively. (a) **NBSe-Cl** (λ_{ex} = 485 nm); (b) **NBS-Cl** (λ_{ex} = 470 nm); and (c) **NBD-Cl** (λ_{ex} = 430 nm).

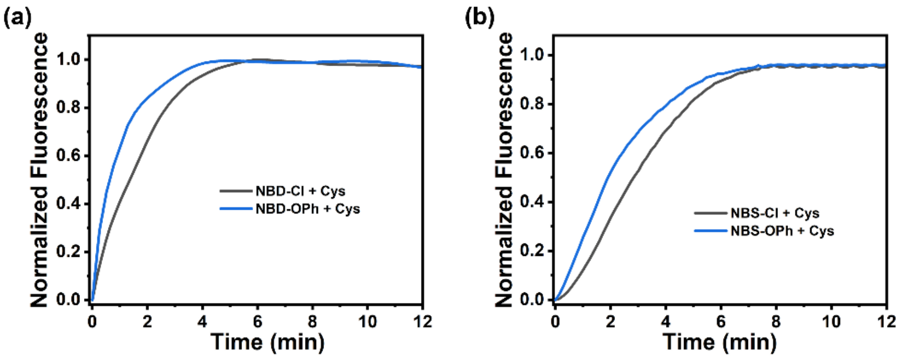

Figure S17 (a) Normalized time-dependent fluorescence intensity at 550 nm of **NBD-Cl** (black) and **NBD-OPh** (blue) after the addition of Cys. λ_{ex} = 450 nm. (b) Normalized time-dependent fluorescence intensity at 553 nm of **NBS-Cl** (black) and **NBS-OPh** (blue) after the addition of Cys. λ_{ex} = 470 nm. The experiments were performed in PBS buffer (pH 7.4, 10 mM, containing 20% DMSO) at room temperature. Probes' concentration: 10 μM; Cys's concentration: 100 μM.

5.2 Fluorescence responses of NBX-based probes toward *n*-butylthiol

In this model study, *n*-butylthiol served as a reactant. The rate constants of the reaction between NBX-R and *n*-butylthiol were obtained based on the second-order reaction kinetics.

The second-order reaction equation: $v = k[A][B]$ (Eq-2)

The rate of disappearance of A is:

$$\frac{d[A]}{dt} = -k[A][B] \qquad \text{(Eq-3)}$$

The integrated rate equation under the condition that $[A]$ and $[B]$ are not equal is:

$$\frac{1}{[B]_0-[A]_0}\ln\frac{[B][A]_0}{[A][B]_0} = kt \qquad \text{(Eq-4)}$$

However, when $[B]_0 >> [A]_0$, then $[B] \approx [B]_0$, then Equation Eq-4 becomes:

$$\frac{1}{[B]_0-[A]_0}\ln\frac{[B][A]_0}{[A][B]_0} \approx \frac{1}{[B]}\ln\frac{[A]_0}{[A]} = kt \qquad \text{(Eq-5)}$$

Or

$$[A] = [A]_0 e^{-[B]kt} \qquad \text{(Eq-6)}$$

This functional form of the decay kinetics is similar to the first order kinetics. That is to say, when the initial concentration of one of the reactants is significantly higher than another, the second-order kinetics can be approximated as first-order kinetics (pseudo first-order kinetics). Based on this assumption, we can write the pseudo-first-order reaction equation as:

$$[A] = [A]_0 e^{-[B]_0 kt} \qquad \text{(Eq-7)}$$

Or

$$[A] = [A]_0 e^{-k't} \qquad \text{(Eq-8)}$$

Where $[A]_0$ is the initial concentration of A, $[B]_0$ is the initial concentration of B, k is the second-order reaction rate constant, k' is the pseudo first-order reaction rate constant, $k' = k[B]_0$, and $[A]$ is the concentration of A at time t.

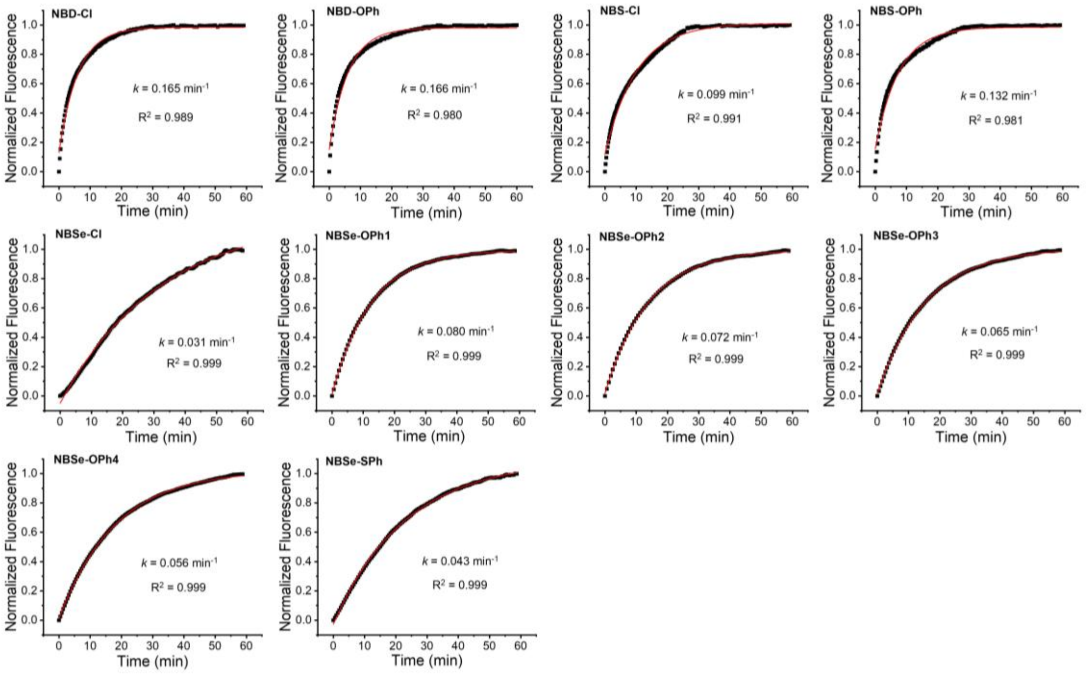

Figure S18 Normalized time-dependent fluorescence measurement of NBX-R probes after the addition of *n*-butylthiol in PBS buffer (*p*H 7.4) containing 10% DMSO at room temperature. NBD-Cl and NBD-OPh: λ_{ex} = 425 nm, λ_{em} = 525 nm; NBS-Cl and NBS-OPh: λ_{ex} = 425 nm, λ_{em} = 510 nm; NBSe-Cl, NBSe-OPh1, NBSe-OPh2, NBSe-OPh3, and NBSe-OPh4: λ_{ex} = 450 nm, λ_{em} = 545 nm. Probes' concentration: 10 μM; *n*-butylthiol's concentration: 200 μM.

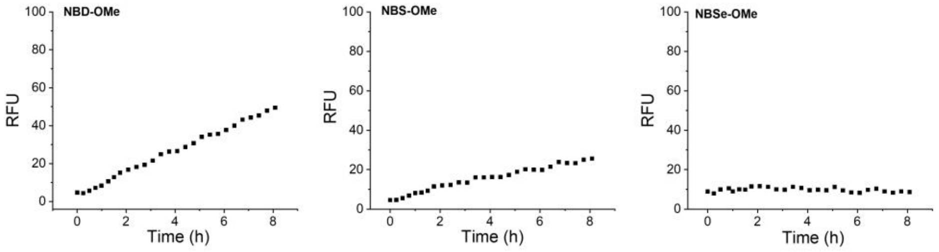

Figure S19 Time-dependent fluorescence measurement of NBD-OMe, NBS-OMe and NBSe-OMe after the addition of *n*-butylthiol in PBS buffer (*p*H 7.4) containing 10% DMSO at room temperature. NBD-OMe: λ_{ex} = 425 nm, λ_{em} = 525 nm; NBS-OMe: λ_{ex} = 425 nm, λ_{em} = 510 nm; NBSe-OMe: λ_{ex} = 450 nm, λ_{em} = 545 nm. Probes' concentration: 10 μM; *n*-butylthiol's concentration: 200 μM.

Table S2. The pseudo first-order reaction rate constants (*k'*) and the second-order reaction rate constants (*k*) of probes NBX-R.

Probes	k' (min^{-1})	k (M^{-1} s^{-1})
NBD-Cl	0.165	13.75
NBD-OPh	0.166	13.83
NBS-Cl	0.099	8.25
NBS-OPh	0.132	11.00
NBSe-Cl	0.031	2.58
NBSe-OPh1	0.080	6.67
NBSe-OPh2	0.072	6.00
NBSe-OPh3	0.065	5.42
NBSe-OPh4	0.056	4.67
NBSe-SPh	0.043	3.58

Note: The kinetic parameters for **NBX-R** toward *n*-butylthiol could be determined using equation Eq-8 by fluorescence method. As shown in Figure S18, the fluorescence signal changes were closely monitored when these probes were incubated with *n*-butylthiol in PBS buffer (pH = 7.4). By fitting the fluorescence data to an exponential equation, the pseudo first-order rate constant (*k'*) of probes NBX-R was determined, as depicted in Table S2. According to $k' = k[B]_0$, the second-order rate constants (*k*) were determined to be 13.75, 8.25 and 2.58 $M^{-1}s^{-1}$ for NBD-Cl, NBS-Cl, and NBSe-Cl, respectively. Probes NBD-OPh, NBS-OPh and NBSe-OPh exhibited the similar tendency. These results implied that the reactivities of similar NBX derivatives with only heteroatom differences are in the order of NBD > NBS > NBSe. The second-order rate constant of NBSe-OPh1, NBSe-OPh2, NBSe-OPh3 and NBSe-OPh4 was 6.67, 6.00, 5.42 and 4.67 $M^{-1}s^{-1}$, which was higher than NBSe-Cl and NBSe-SPh. Especially, NBSe-OPh1 displayed the highest *k*, owing to the leaving ability and water-solubility of its substituent.

In addition, all alkoxy-substituted NBX derivatives displayed low reactivity (Figure S19): they required 60 min to obtain detectable signals after incubating NBD-OMe/NBS-OMe with *n*-butylthiol, and their fluorescence intensity steadily increased over an 8-h test; NBSe-OMe was nearly inert to *n*-butylthiol and no observable fluorescence signals were obtained after 8-h incubation. Therefore, the rate constants of NBX-OMe with *n*-butylthiol can not be determined.

(a)

Compounds	*k* (M^{-1} s^{-1})	σ_p
NBSe-OPh1	6.67	0.45
NBSe-OPh2	6.00	0.78
NBSe-OPh3	5.42	0.00
NBSe-OPh4	4.67	-0.27

(b)

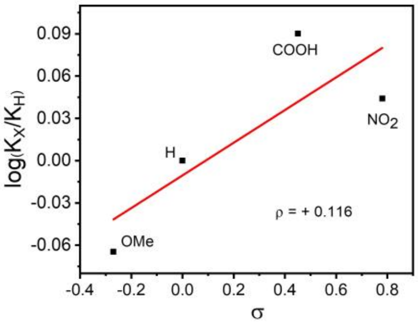

Figure S20 (a) The second-order reaction rate constants (*k*) of probes NBSe-OPh1, NBSe-OPh2, NBSe-OPh3 and NBSe-OPh4. (b) The Hammett plots from (a) were linearly fitted to give the slope as $\rho = + 0.116$. In the vertical axis ($\log(K_X/K_H)$), K_X represents the kinetic constants of NBSe-OPh1, NBSe-OPh2 and NBSe-OPh4 in (a), K_H represents the kinetic constant of NBSe-OPh3.

Note: Among these probes, NBSe-OPh1, NBSe-OPh2, NBSe-OPh3 and NBSe-OPh4 can be studied based on Hammett's correlation. As shown in Figure S20, the construction of a Hammett plot using σ_p values[8] revealed a positive slope with reaction constant $\rho = + 0.116$. This result implies that the S_N2 reaction is facilitated by electron-withdrawing moieties in the phenoxy substituents. In addition, NBSe-OPh1 with a -COOH moiety displayed a faster reaction rate than NBSe-OPh2 with a -NO_2 moiety, contributing to the slightly lower value of ρ. This increased reaction rate is likely due to the enhanced solubility provided by the -COOH group.

6. Supplementary data for protein thiols profiling

6.1 Fluorescence responses of NBSe-based probes towards bovine β-lactoglobulin

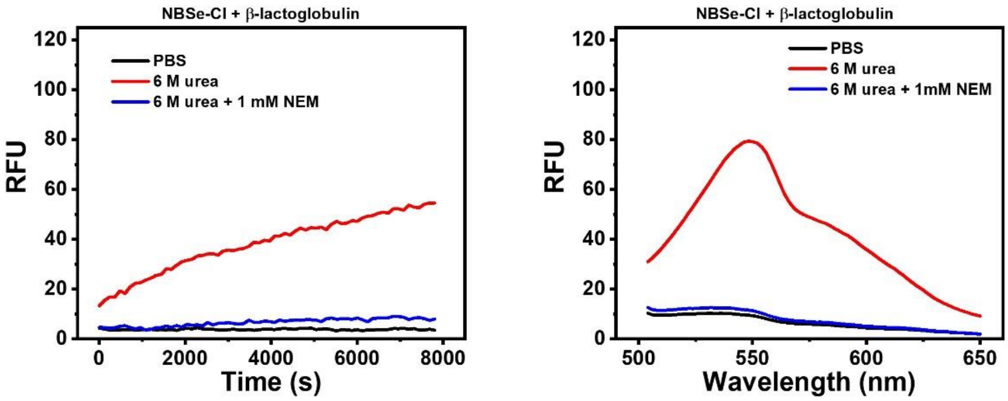

Figure S21 Time-dependent fluorescence intensity at 548 nm (left) and fluorescence spectra (right) of **NBSe-Cl** (10 μM) with bovine β-lactoglobulin (100 μM) in different testing media at room temperature. Black line: PBS buffer (pH 7.4, 10 mM) containing 10% DMSO; red line: 6 M urea; blue line: 6 M urea containing 1 mM N-ethylmaleimide (NEM). λ_{ex} = 450 nm; λ_{em} = 548 nm.

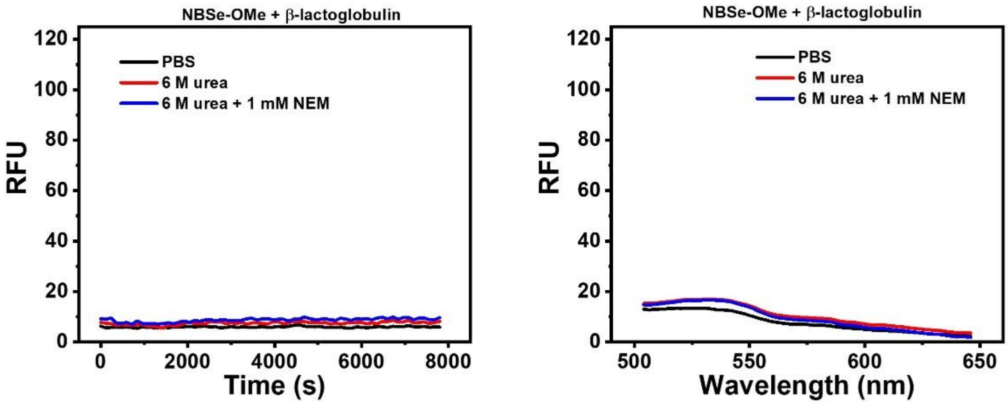

Figure S22 Time-dependent fluorescence intensity at 548 nm (left) and fluorescence spectra (right) of **NBSe-OMe** (10 μM) with bovine β-lactoglobulin (100 μM) in different testing media at room temperature. Black line: PBS buffer (pH 7.4, 10 mM) containing 10% DMSO; red line: 6 M urea; blue line: 6 M urea containing 1 mM N-ethylmaleimide (NEM). λ_{ex} = 450 nm; λ_{em} = 548 nm. **NBSe-OMe** demonstrated low reactivity and did not react with cysteine residues in proteins, irrespective of the protein's folding state.

6.2 Intact protein mass and site-mapping experiments of bovine β-lactoglobulin

Intact protein mass

Bovin β-lactoglobulin (5 μM) was incubated with NBSe-OPh1 (20 μM) in 6 M urea or in PBS buffer at room temperature for 1 h. For desalination, cold acetone (4 equiv.) was added to the samples followed by centrifugation (4 °C, 12000 rpm, 1 min). After that, the desalted samples were dissolved with 0.1% formic acid and the labeled protein samples (estimated final protein concentration to be 10 μM) was obtained. 6 μL of the sample was analyzed by mass spectrometry as described above. After charge envelope deconvolution, relative amounts of unmodified β-lactoglobulin and the covalent adduct were determined by quantifying the mass peak intensities using Mass lynx V4.2 software (Waters, USA).

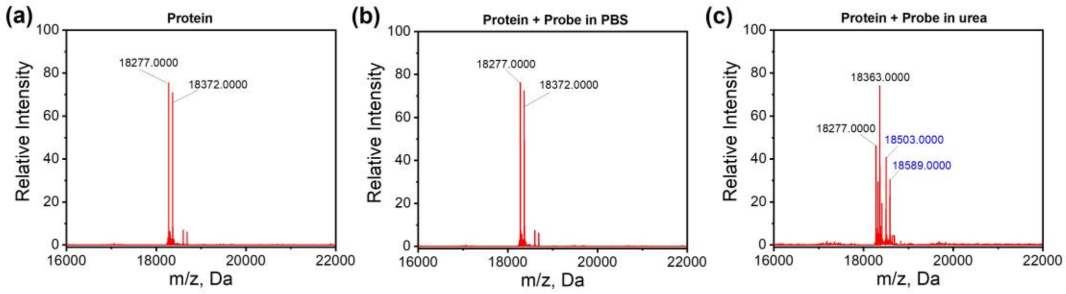

Figure S23 Intact protein MS assay. (a) Pure protein β-lactoglobulin. (b) β-lactoglobulin (5 μM) incubated with NBSe-OPh1 (20 μM) in PBS 7.4 buffer at room temperature for 1 h. (c) β-lactoglobulin (5 μM) incubated with NBSe-OPh1 (20 μM) in 6 M urea at room temperature for 1 h.

Note: As shown in Figure S23a, pure protein β-lactoglobulin showed two MW peaks at 18277 Da and 18372 Da. When incubated with probe **NBSe-OPh1** in PBS buffer at room temperature for 1 h, β-lactoglobulin displayed no changes at mass spectrometry (Figure S23b). In contrast, two new protein peaks (+226 Da) was detected by intact MS after urea treatment, indicating that one cysteine reacted with probe (Figure 23c).

Site-mapping analysis

Bovin β-lactoglobulin (5 μM) was incubated with NBSe-OPh1 (40 μM) in 6 M urea or in PBS buffer at room temperature for 1 h. Then cold acetone (4 equiv.) was added to the samples followed by centrifugation to remove small molecules. After that, the complexes were resuspended in 150 μL of 1 × PBS buffer, supplemented with 5 mM DTT and incubated at 37 °C for 45 min to eliminate any possible disulfide bond. Then the cysteines in β-lactoglobulin were alkylated by adding IAA at a final concentration of 15 mM and incubated at room temperature for 1 h in the dark. Subsequently, the complexes were cleaned by SP3 beads according to the manual, resuspended in 150 μL of 20 mM NH_4HCO_4, and digested by trypsin (trypsin: protein = 1: 50) at 37 °C for overnight. After digestion, the produced peptides were isolated and dried by vacuum centrifugation, resuspended by 50 μL of 0.1% formic acid. 50 ng peptides were then analyzed by LC-MS/MS. The LC-MS/MS experiment was performed on an UltiMate 3000 Nano HPLC system coupled to an Orbitrap Exploris 480 (Thermo Fisher Scientific). Peptides were separated with a 90-min gradient (buffer A:

0.1% FA in deionized water; buffer B: 0.1% FA in 80% acetonitrile with a flow rate of 0.3 μL/min). Data processing was performed on Proteome Discoverer software. Two missed cleavage sites of trypsin were allowed. Carbamidomethyl/+57.021 Da (C), Probe modification/ 226.923 Da (C) and Oxidation/+15.995 Da (M) were used as variable modifications. The samples were searched against a protein database (Uniprot, P02754). All spectra used a target false discovery rate (FDR) of 1%.

#1	b^+	b^{2+}	b^{3+}	Seq.	y^+	y^{2+}	y^{3+}	#2
1	164.07061	82.53894	55.36172	Y				23
2	277.15467	139.08097	93.05641	L	2825.10531	1413.05629	942.37329	22
3	390.23873	195.623	130.7511	L	2712.02125	1356.51426	904.6786	21
4	537.30715	269.15721	179.7739	F	2598.93718	1299.97223	866.98391	20
5	697.3378	349.17254	233.11745	C-Carbamidomethyl	2451.86877	1226.43802	817.96111	19
6	828.37828	414.69278	276.79761	M	2291.83812	1146.4227	764.61756	18
7	957.42087	479.21407	319.81181	E	2160.79763	1080.90246	720.9374	17
8	1071.4638	536.23554	357.82612	N	2031.75504	1016.38116	677.9232	16
9	1158.49583	579.75155	386.83679	S	1917.71211	959.3597	639.90889	15
10	1229.53294	615.27011	410.51583	A	1830.68009	915.84368	610.89821	14
11	1358.57554	679.79141	453.53003	E	1759.64297	880.32512	587.21917	13
12	1455.6283	728.31779	485.88095	P	1630.60038	815.80383	544.20498	12
13	1584.67089	792.83908	528.89515	E	1533.54761	767.27745	511.85406	11
14	1712.72947	856.86837	571.58134	Q	1404.50502	702.75615	468.83986	10
15	1799.7615	900.38439	600.59202	S	1276.44644	638.72686	426.15367	9
16	1912.84556	956.92642	638.28671	L	1189.41442	595.21085	397.14299	8
17	1983.88268	992.44498	661.96574	A	1076.33035	538.66881	359.4483	7
18	2143.91332	1072.4603	715.30929	C-Carbamidomethyl	1005.29324	503.15026	335.76926	6
19	2271.9719	1136.48959	757.99548	Q	845.26259	423.13493	282.42571	5
20	2601.90448	1301.45588	867.97301	C-ModRXJ	717.20401	359.10564	239.73952	4
21	2714.98855	1357.99791	905.6677	L	387.27143	194.13935	129.76199	3
22	2814.05696	1407.53212	938.69051	V	274.18737	137.59732	92.06731	2
23				R	175.11895	88.06311	59.0445	1

Figure S24 The mass list of fragment ion detected by LC-MS/MS for digested β-lactoglobulin (5 μM) incubated with NBSe-OPh1 (40 μM) in 6 M urea at room temperature for 1 h.

6.3 Fluorescence responses of NBSe-based probes toward GSTP1

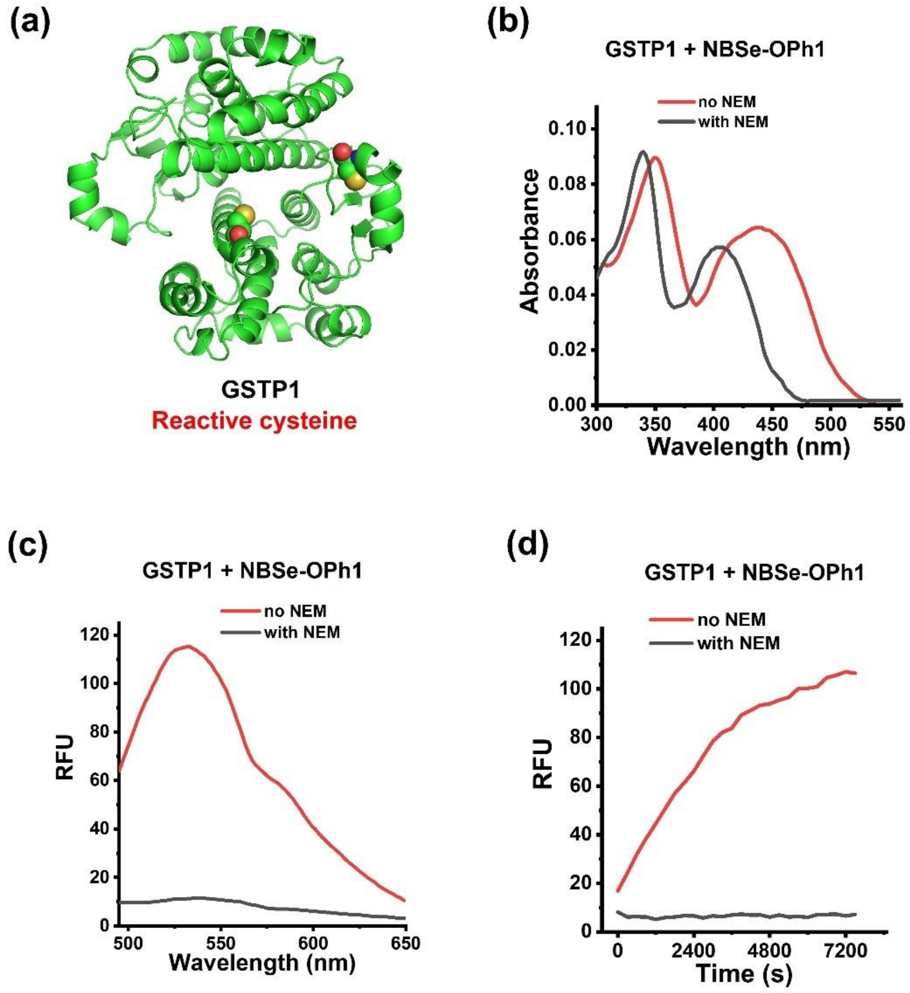

Figure S25 (a) Structure of GSTP1 containing four cysteines (Cys47 and Cys101 are reactive). Absorbance (b) and fluorescence (c) of **NBSe-OPh1** (10 μM) in response to GSTP1 (40 μM) in PBS buffer (pH 7.4, 10 mM, containing 10% DMSO) at room temperature. (d) Time-dependent fluorescence intensities at 530 nm of **NBSe-OPh1** (10 μM) with GSTP1 (40 μM) in PBS buffer. λ_{ex} = 450 nm. NEM's concentration: 1 mM.

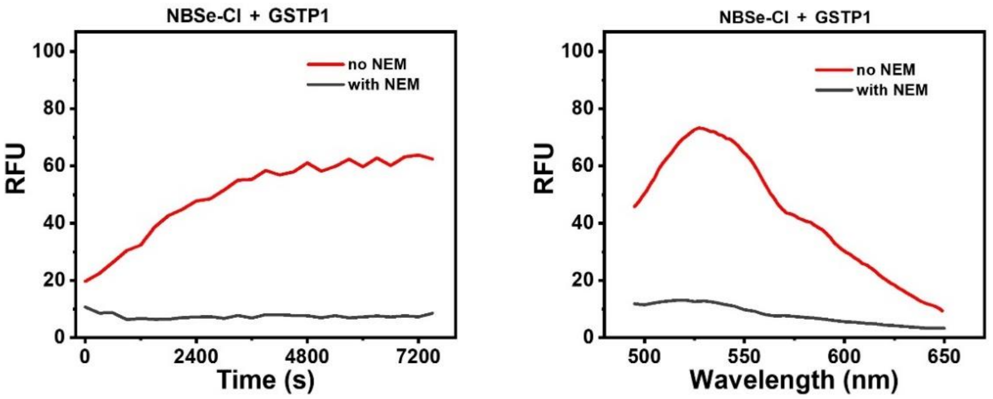

Figure S26 Time-dependent fluorescence intensities at 530 nm (left) and fluorescence spectra (right) of **NBSe-Cl** (10 μM) with GSTP1 (40 μM) in PBS buffer (pH 7.4, 10 mM, containing 10% DMSO) at room temperature in the absence (red line) and presence (black line) of NEM (1 mM), respectively.

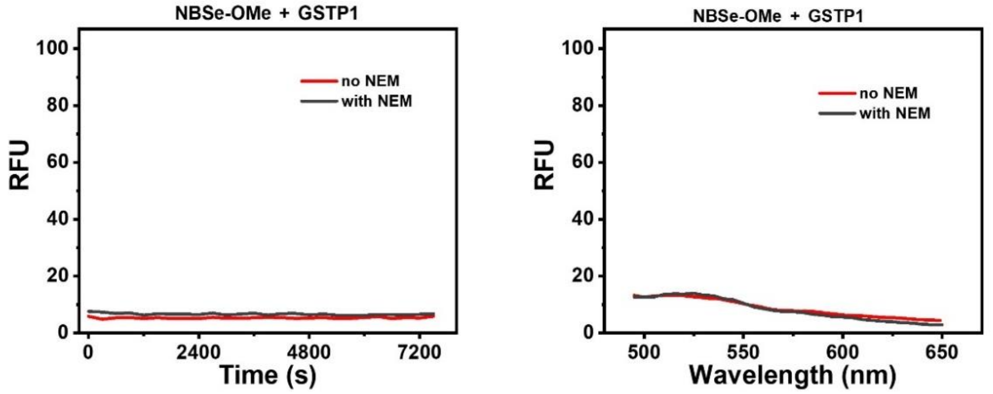

Figure S27 Time-dependent fluorescence intensities at 530 nm (left) and fluorescence spectra (right) of **NBSe-OMe** (10 μM) with GSTP1 (40 μM) in PBS buffer (pH 7.4, 10 mM, containing 10% DMSO) at room temperature in the absence (red line) or presence (black line) of NEM (1 mM).

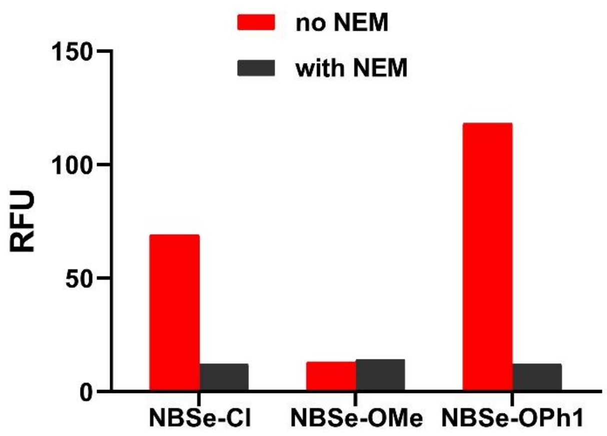

Figure S28 Fluorescence intensities of **NBSe-Cl**, **NBSe-OMe** and **NBSe-OPh1** in response to GSTP1 in PBS buffer (pH 7.4, 10 mM, containing 10% DMSO) at room temperature for 2 hours. Probes' concentration: 10 μM; NEM's concentration: 1 mM. λ_{ex} = 450 nm.

6.4 Fluorescence responses of NBSe-based probes towards HeLa lysate

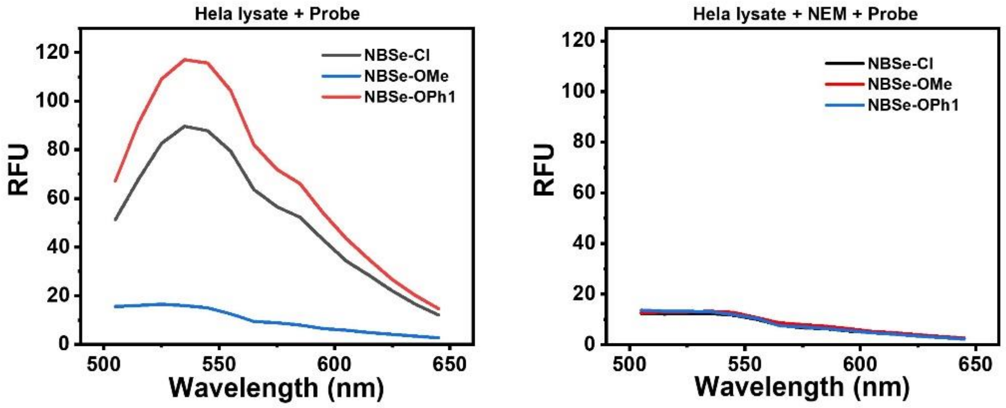

Figure S29 Fluorescence spectra of HeLa cell lysate in the absence (left) and presence (right) of NEM (10 mM) after a 2-hour incubation with **NBSe-Cl**, **NBSe-OMe** and **NBSe-OPh1**, respectively, in PBS buffer (pH 7.4, 10 mM, containing 10% DMSO) at room temperature. The concentration of each probe is 10 μM. GSH and other small molecules (below 10 kDa) were removed from HeLa lysate by centrifugal ultrafiltration method. λ_{ex} = 450 nm. Results showed that HeLa lysate could react with **NBSe-Cl** and **NBSe-OPh1** to generate fluorescent **NBSe-SR** products with a λ_{em}^{max} = 546 nm.

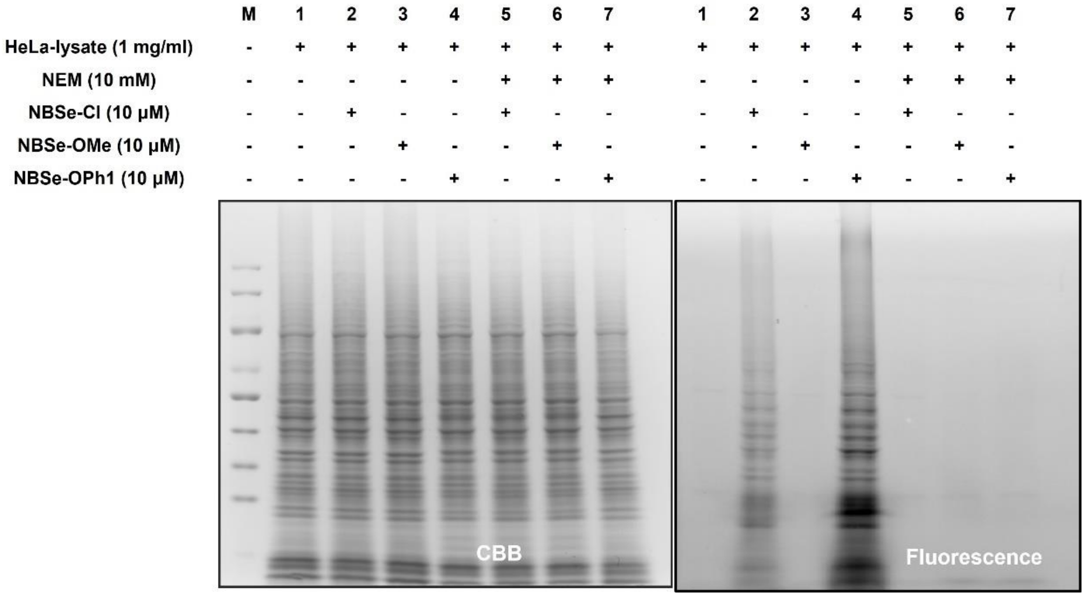

Figure S30 In-gel CBB and fluorescence labeling of HeLa cell lysate treated with probes **NBSe-Cl**, **NBSe-OMe** and **NBSe-OPh1**, respectively, in the absence or presence of NEM. The fluorescence images were taken at 546 nm with an excitation at 450 nm. The labeling conditions were as follows: 1 mg/mL HeLa cell lysate was incubated with 10 μM of each probe in 10 mM PBS buffer containing 10% DMSO for 2 h at room temperature. In lanes 5-7, HeLa lysate was preincubated with 10 mM NEM for 30 min at room temperature before the addition of each probe. Results revealed that both **NBSe-Cl** and **NBSe-OPh1** were reactive to a broad range of cellular proteins.

6.5 Fluoresecence responses of NBD-based probes towards HeLa lysate

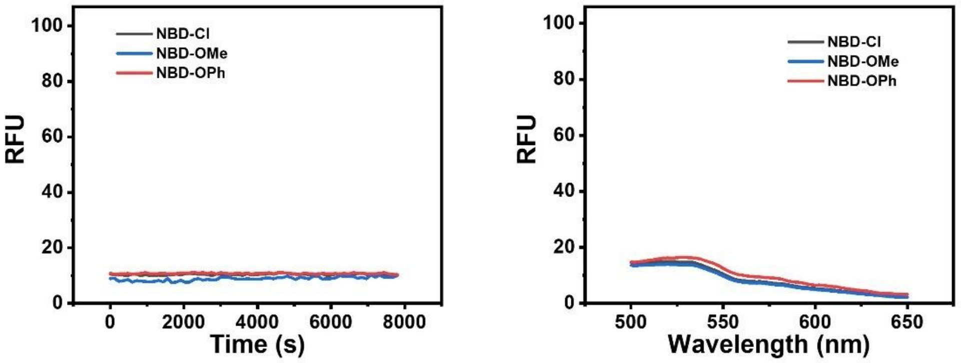

Figure S31. Time-dependent fluorescence intensities at 540 nm (left) and fluorescence spectra (right) of **NBD-Cl**, **NBD-OMe** and **NBD-OPh** in PBS buffer (pH 7.4, 10 mM, containing 10% DMSO) at room temperature. The concentration of each probe is 10 μM. λ_{ex} = 450 nm. Results showed that all three probes alone showed no changes in fluorescence signal over time.

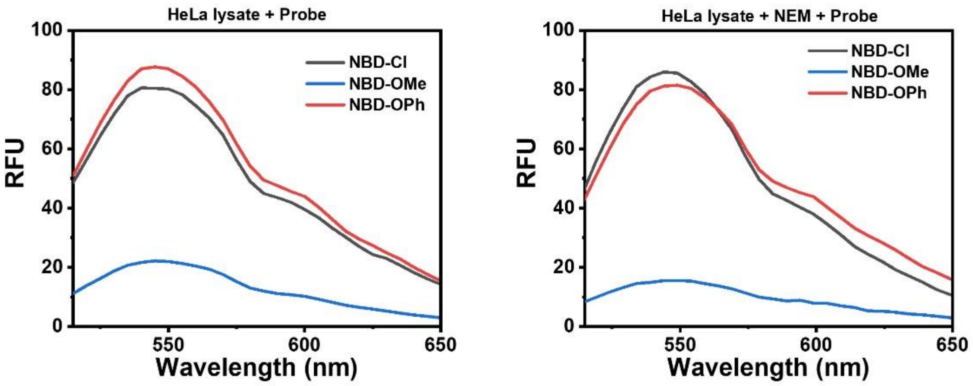

Figure S32. Fluorescence spectra of **NBD-Cl**, **NBD-OMe** and **NBD-OPh** after 2-hour incubation in HeLa cell lysate without (left) or with (right) NEM pretreatment, respectively. The concentration of each probe is 10 μM. GSH and other small molecules (below 10 kDa) were removed from HeLa lysate by centrifugal ultrafiltration method. All the tests were carried out in PBS buffer (pH 7.4, 10 mM, containing 10% DMSO) at room temperature. λ_{ex} = 450 nm.

Note: NBD-Cl and **NBD-OPh** exhibited high reactivity to HeLa lysate resulting in emission intensity enhancement at 540 nm (Figure S32). The fluorescence enhancement is probably due to the formation of amino-NBD adducts. Despite the addition of NEM into HeLa lysate to block the thiols, there were still remarkable fluorescence signals, suggesting other species in addition to thiols in the cell lysate reacted with probes.

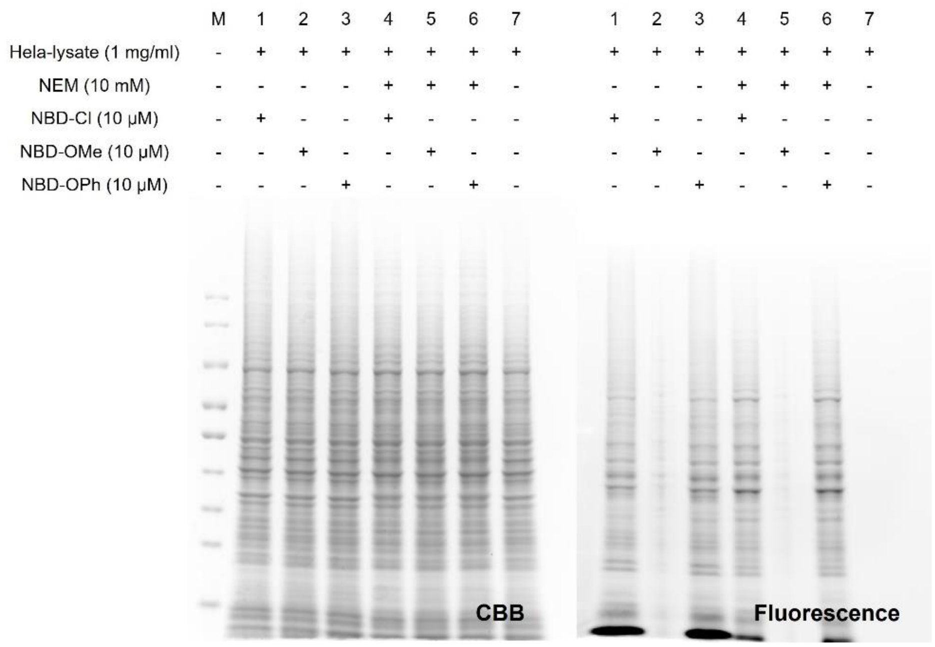

Figure S33. In-gel CBB and fluorescence labeling of HeLa cell lysate treated with probes **NBD-Cl**, **NBD-OMe** and **NBD-OPh**, respectively, in the absence or presence of NEM. The fluorescence images were captured at 550 nm with an excitation wavelength of 488 nm. The labeling conditions were as follows: 1 mg/mL HeLa cell lysate was incubated with 10 μM of each probe in 10 mM PBS buffer containing 10% DMSO at room temperature for 2 h. In lanes 4-6, HeLa lysate was preincubated with 10 mM NEM for 30 min at room temperature before adding each probe.

Note: In-gel fluorescent scanning results revealed that **NBD-Cl** and **NBD-OPh** could react with a wide variety of proteins regardless of the absence or presence of NEM. These findings suggested that both **NBD-Cl** and **NBD-OPh** exhibited poor selectivity in labeling cysteine residues of proteins.

6.6 IsoTOP-ABPP assay of NBSe-based probes responding to HeLa lysate

Preparation of IsoTOP-ABPP (isotopic tandem orthogonal proteolysis protein profiling) **samples**

HeLa cells were lysed and small molecules including biothiols were carefully removed by ultracentrifugation method. HeLa lysate (2 mg/mL) was incubated at room temperature for 3 h with 50 µM of probes **NBSe-OPh1** and **NBSe-Cl**, respectively. In the control sample, DMSO was used to replace the probes. Then, IA-alkyne (10 µM) was added to each sample, and the obtained mixture was incubated for 1 hour in the dark. Next, the light Az-UV-biotin (100 µM) was added to the probe-treated samples, and the heavy Az-UV-biotin (100 µM) was added to the DMSO-treated sample. Finally, click reagents (100 µM THPTA, 1 mM $CuSO_4$ and 2 mM sodium ascorbate) were added to the samples, which were incubated for 2 h in the dark.

The probe-treated and DMSO-treated samples were mixed in methanol, vortexed and centrifuged. The top layer was aspirated, and methanol was added, followed by centrifugation and aspiration of the supernatant. The pellets were resuspended with PBS buffer containing 1% SDS, and then mixed with 100 µL of neutravidin agarose resin. The samples were incubated with rotation for 4 h at room temperature.

The beads were pelleted by centrifugation, and washed two times with PBS containing 1% SDS, two times with PBS containing 0.1% SDS and three times with PBS. Subsequently, the beads were resuspended in 6 M Urea, and 25 µL of 100 mM DTT in 25 mM ammonium bicarbonate buffer were added, followed by incubation at 37°C for 45 min. After centrifugation at 800 rpm for 5 minutes to remove the supernatant, the beads were washed three times with PBS. Next, the beads were dissolved in 6 M Urea, and 25 µL of 400 mM IA-alkyne in 25 mM ammonium bicarbonate buffer were added, followed by incubation in the dark at 37°C for 45 min.

The beads were pelleted, and washed three times with PBS, then resuspended with 200 µL of 2 M Urea in PBS. At this point, 2 µL of 100 mM calcium chloride solution and 4 µg of trypsin were added to each sample, followed by overnight incubation at 37°C with shaking. The samples were centrifuged to remove the supernatant, and the obtained beads were washed three times with double distilled water. Then, 200 µL of 0.1% formic acid aqueous solution was added, and the mixture was irradiated under 365 nm UV lamp for 1 hour. The supernatant was collected by centrifugation to obtain peptides. The peptides were purified on a C18 desalting column eluting with methanol, acetonitrile and water (containing 0.1% TFA). The purified peptides were analyzed by LC-MS/MS.

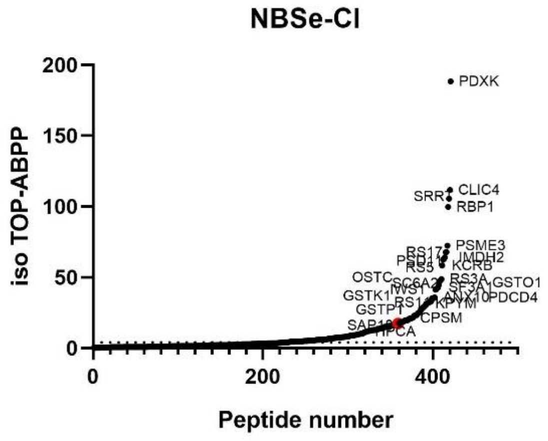

Figure S34 IsoTOP-ABPP ratio analysis of reactive cysteine residue in HeLa lysate treated with **NBSe-Cl**.

Table S3. Representative cysteine residues identified by **NBSe-OPh1** with high isoTOP-ABPP ratios.

Protein name	Peptide	Labeling sites	Function	Ratio
SAP18	TCPLLLR	Cys26	Unannotated	310.23
CPSM	VVAVDCGIK	Cys225	Unannotated	302.86
HPCA	LLQCDPSSASQF	Cys185	Unannotated	289.55
S10AA	DLDQCR	Cys62	Unannotated	217.19
IWS1	ALRPGDPGFCAR	Cys749	Unannotated	202.8
KPYM	GIFPVLCK	Cys152	Unannotated	185.71
IMDH2	HGFCGIPITDTGR	Cys140	Unannotated	169.48
GSTP1	ASCLYGQLPK	Cys47	Active site	150.3
PCBP1	INISEGNCPER	Cys54	Unannotated	122.45
ERD22	SCAGISGK	Cys29	Unannotated	110.92
SRRT	WLCPLSGK	Cys715	Unannotated	99.84
PSME2	CGFLPGNEK	Cys91	Unannotated	99.52
PDCD4	DLCPSR	Cys288	Unannotated	77.2
OSTC	VPFLVLECPNLK	Cys14	Unannotated	54.09
CLIC4	IGNCPFSQR	Cys35	Unannotated	42.4
GSTO1	FCPFAER	Cys32	Active site	10.84
RL14	ALVDGPCTQVR	Cys42	Unannotated	230.59
RS5	AQCPIVER	Cys155	Unannotated	142.99
RS21	TYAICGAIR	Cys56	Unannotated	137.83
RS17	VCEEIAIIPSKK	Cys35	Unannotated	80.65

Table S4. Representative cysteine residues identified by **NBSe-Cl** with high isoTOP-ABPP ratios.

Protein name	Peptide	Labeling sites	Function	Ratio
PDXK	TIQCAK	Cys273	Unannotated	188.4
CLIC4	IGNCPFSQR	Cys35	Unannotated	111.71
SRRT	WLCPLSGK	Cys715	Unannotated	105.65
PSME3	RLDECEEAFQGTK	Cys92	Unannotated	72.4
IMDH2	HGFCGIPITDTGR	Cys140	Unannotated	64.12
KCRB	LGYILTCPSNLGTGLR	Cys283	Unannotated	58.34
GSTO1	FCPFAER	Cys32	Active site	47.32
SF3A1	EVLDQVCYR	Cys244	Unannotated	42.69
OSTC	VPFLVLECPNLK	Cys14	Unannotated	41.64
IWS1	ALRPGDPGFCAR	Cys749	Unannotated	41.34
PDCD4	GTVDCVQAR	Cys288	Unannotated	35.28
GSTK1	ETTEAACR	Cys176	Unannotated	32.83
KPYM	GIFPVLCK	Cys152	Unannotated	31.53
GSTP1	ASCLYGQLPK	Cys47	Active site	17.37
CPSM	VVAVDCGIK	Cys225	Unannotated	21.23
SAP18	TCPLLLR	Cys26	Unannotated	16.01
RS17	VCEEIAIIPSKK	Cys35	Unannotated	67.74
RS5	AQCPIVER	Cys155	Unannotated	62.91
RS3A	ACQSIYPLHDVFVR	Cys201	Unannotated	48.8
RS11	CPFTGNVSIR	Cys60	Unannotated	33.08

Note: We performed isoTOP-ABPP experiments to check the reactivity of probe **NBSe-Cl** and **NBSe-OPh1**. A total of 584 proteins were detected by **NBSe-OPh1**, 344 of which yielded a ratio of > 4. Moreover, **NBSe-Cl** detected a total of 421 proteins, of which, 188 proteins with a ratio > 4. There are 151 proteins detected by both **NBSe-Cl** and **NBSe-OPh1**, such as ribosomal proteins RS5, inosine-5'-monophosphate dehydrogenase 2 (IMDH2), pyruvate kinase PKM (KPYM) and GSTP1, indicating that these two probes have similar proteomic selectivity (Tables S3 and S4). Notably, Cys47 in GSTP1 was detected by **NBSe-OPh1** and yielded a ratio of 150.3 (Table S3), signifying that it is a highly reactive site, which is in a good agreement with the pure GSTP1 assay.

6.7 Stability analysis of thiol-addition product of NBSe-based probes

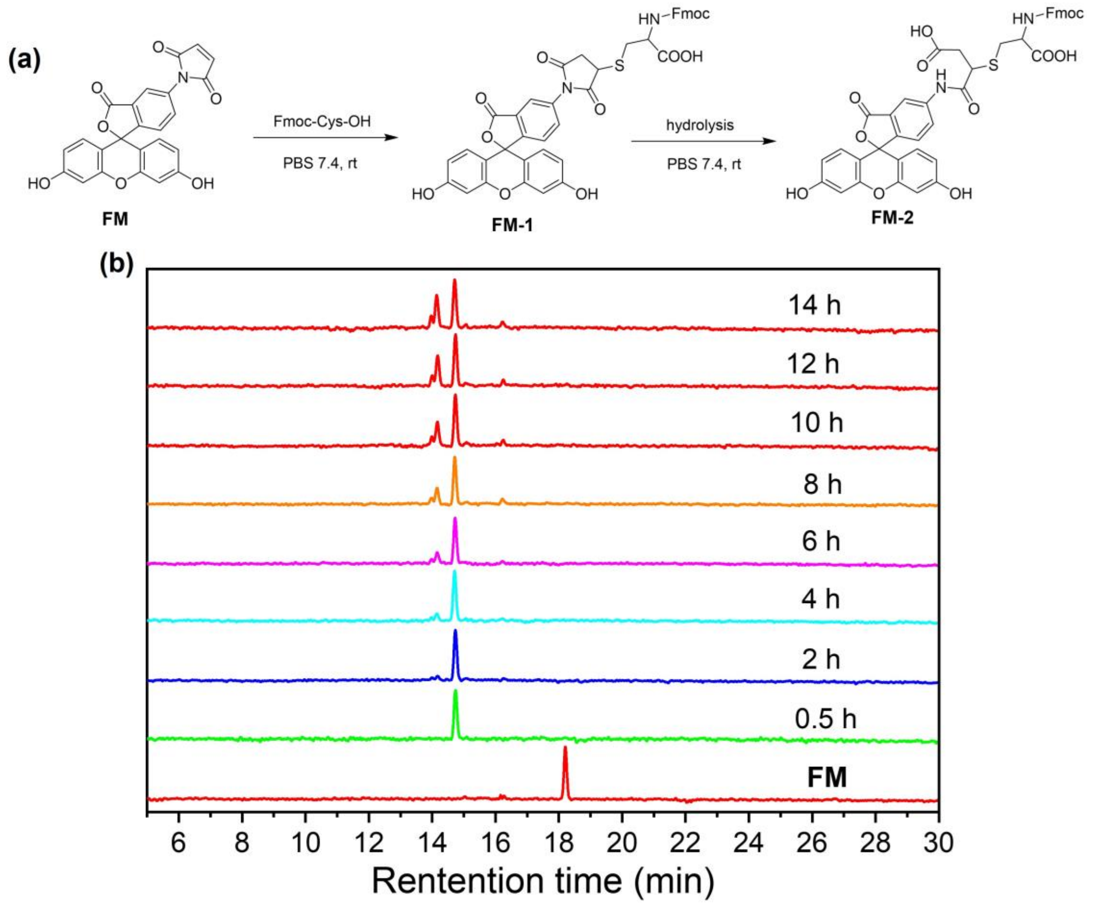

Figure S35 Reversed-phase HPLC analysis of the reaction mixture of **FM** with Fmoc-Cys-OH with a detection wavelength of 495 nm. The HPLC assay conditions are as follows: Probe **FM** concentration: 20 μM; Fmoc-Cys-OH: 200 μM; incubation conditions: PBS 7.4 buffer, room temperature.

Note: As shown in Figure S35, probe **FM** initially exhibited a peak with a retention time of 18.2 min. After incubation with Fmoc-Cys-OH for 30 min, a new peak appeared at 14.8 min, corresponding to the thiol-addition product **FM-1**. After 4 hours, other peaks emerged at 13.9 minutes and gradually increased over time. These peaks correspond to the hydrolysis product FM-2 or other side products. We observed that these side products reached half of the total content after 14 hours. In sharp contrast, when our probe **NBSe-OPh3** was incubated with Fmoc-Cys-OH for 14 h, only a single peak at 12.6 min was observed (Figure S36), indicating the excellent stability of the adduct **NBSe-Cys-Fmoc**. These results suggested that NBSe-based probes can form stable reaction products in the modification of cysteine in proteins.

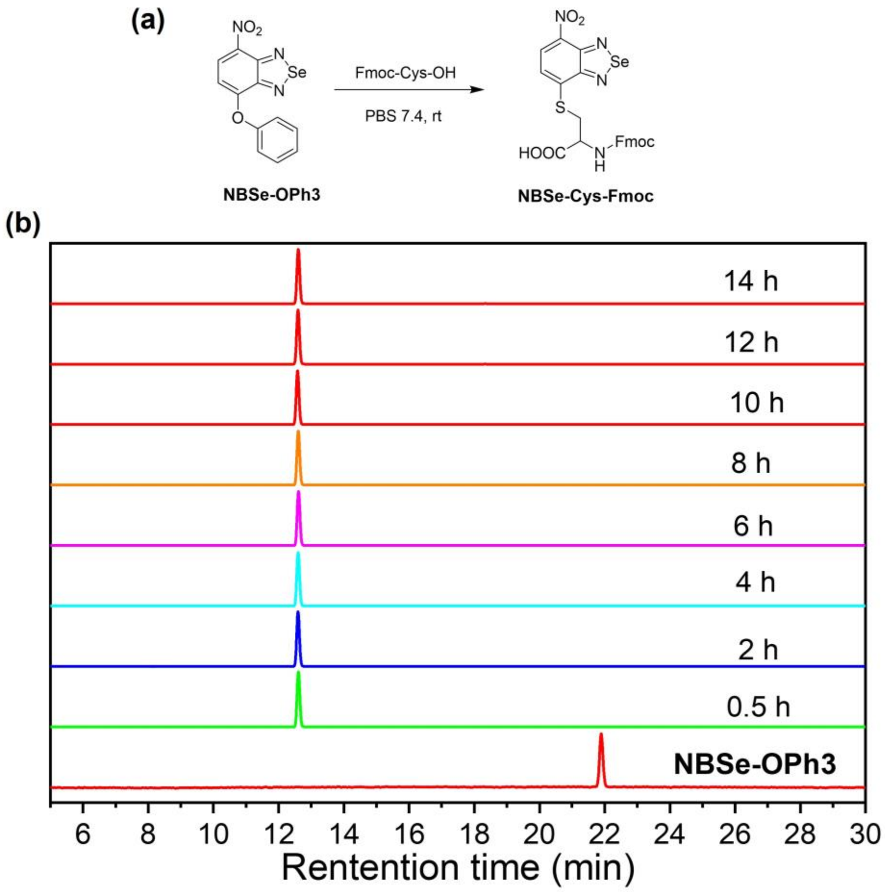

Figure S36 Reversed-phase HPLC analysis of the reaction mixture of **NBSe-OPh3** with Fmoc-Cys-OH with a detection wavelength of 450 nm. The HPLC assay conditions are as follows: Probe **NBSe-OPh3** concentration: 20 μM; Fmoc-Cys-OH: 200 μM; incubation conditions: PBS 7.4 buffer, room temperature.

7. Supplementary data for GSH-activated PDT

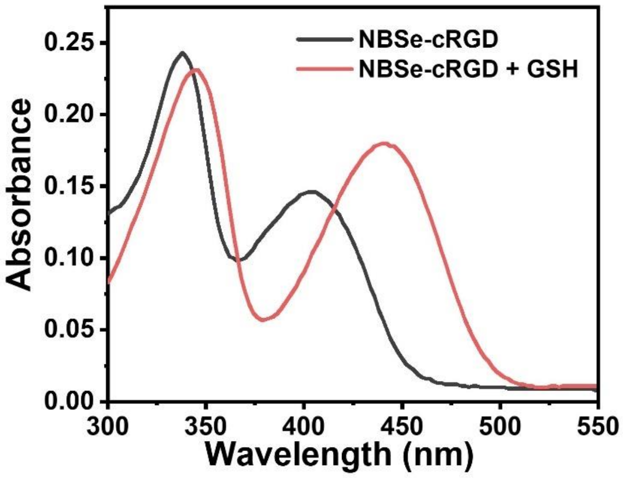

Figure S37 Absorption responses of **NBSe-cRGD** (10 μM) towards GSH (500 μM) in PBS buffer (pH 7.4, 10 mM) at room temperature.

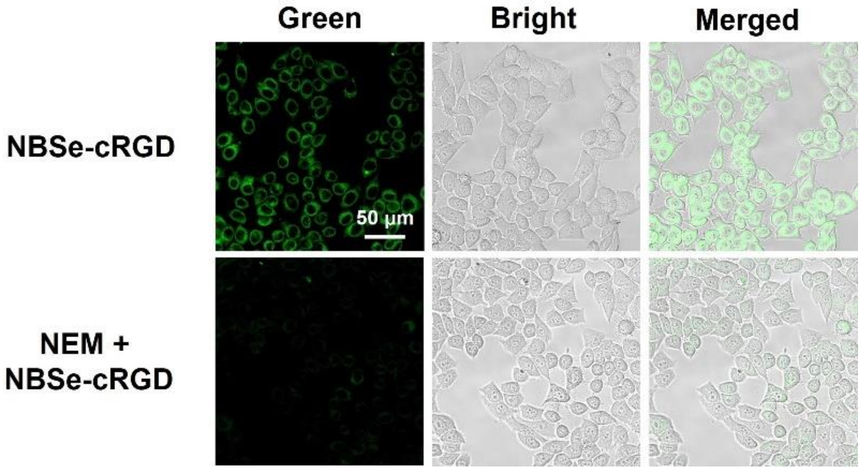

Figure S38 Fluorescence imaging of HeLa cells with **NBSe-cRGD**. Top row: cells were incubated with **NBSe-cRGD** (10 μM) for 60 min; bottom row: cells were pre-incubated with NEM (10 mM) for 30 min, and then incubated with **NBSe-cRGD** (10 μM) for 60 min. λ_{ex} = 488 nm; λ_{em} = 500-550 nm.

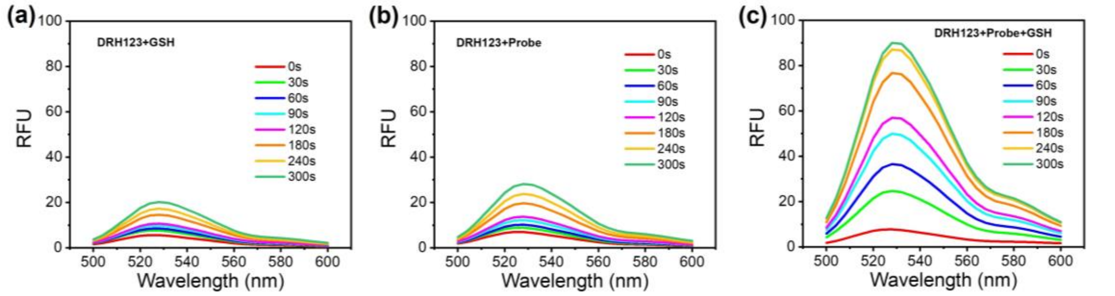

Figure S39 Changes of the emission spectra of DHR123 (10 μM) under different conditions in PBS 7.4 buffers with light irradiation. (a) GSH (100 μM); (b) Probe NBSe-cRGD (10 μM); and (c) the mixture of GSH and probe **NBSe-cRGD**. LED light: 400-800 nm, 30 mW/cm^2. λ_{ex} = 460 nm.

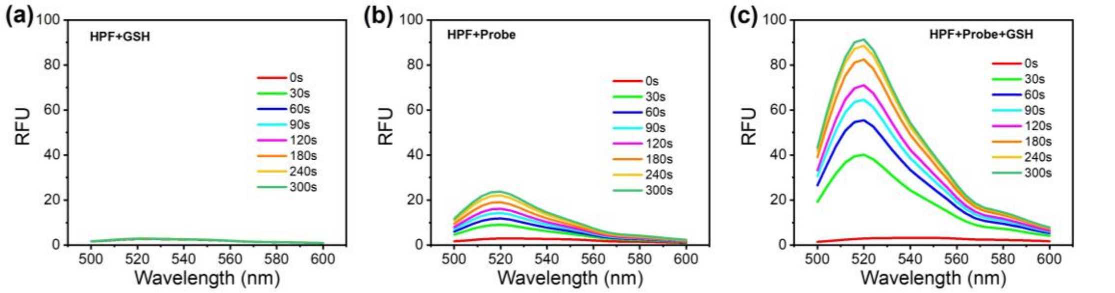

Figure S40 Changes of the emission spectra of HPF (10 μM) under different conditions in PBS 7.4 buffers with light irradiation. (a) GSH (100 μM); (b) Probe **NBSe-cRGD** (10 μM); and (c) the mixture of GSH and probe NBSe-cRGD. LED light: 400-800 nm, 30 mW/cm^2. λ_{ex} = 460 nm.

Note: After 5 min of LED illumination, the fluorescence of DHR123 increased rapidly in the presence of **NBSe-cRGD** and GSH, indicating $O_2^{-\bullet}$ generation (Figure S39). In the absence of **NBSe-cRGD** or GSH, only slight increase in the fluorescence of DHR123 was observed. The similar phenomenon was observed in HPF groups, implying OH• generation of **NBSe-cRGD** in the presence of GSH (Figure S40). These results suggest that the GSH-activated photosensitizer **NBSe-cRGD** is capable of generating ROS. Our previous ABDA assay also demonstrated its singlet oxygen generation ability. Therefore, it acts as both a type 1 and type 2 photosensitizer, with its PDT effect resulting from the contribution of both ROS and singlet oxygen.

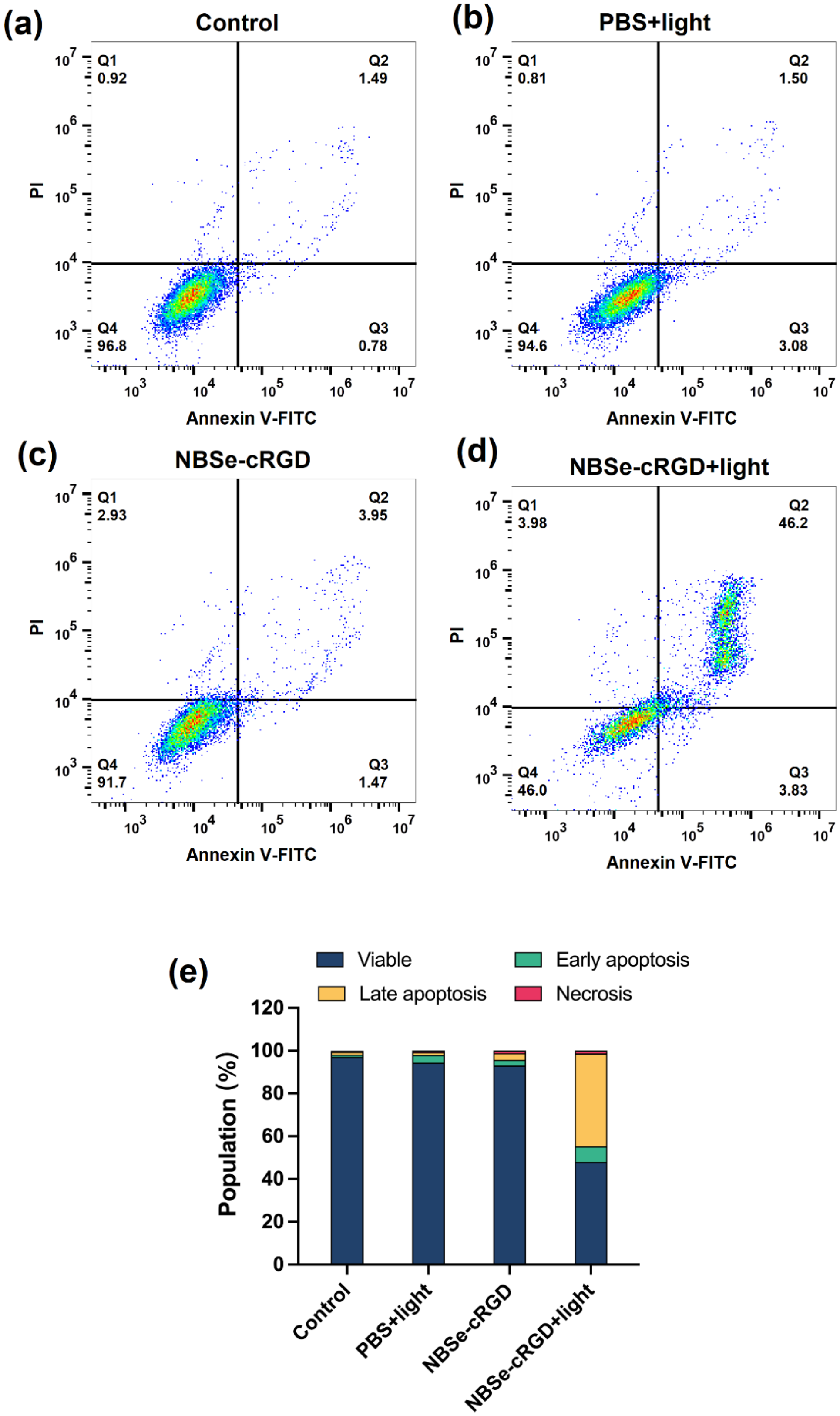

Figure S41 Cell apoptosis study by flow cytometry analysis of HeLa cells incubated with **NBSe-cRGD** (10 µM) after LED irradiation (400-800 nm, 30 mW/cm^2, 15 min).

8. Supplementary data for reactivity study of alkoxy-NBSe toward Lysine

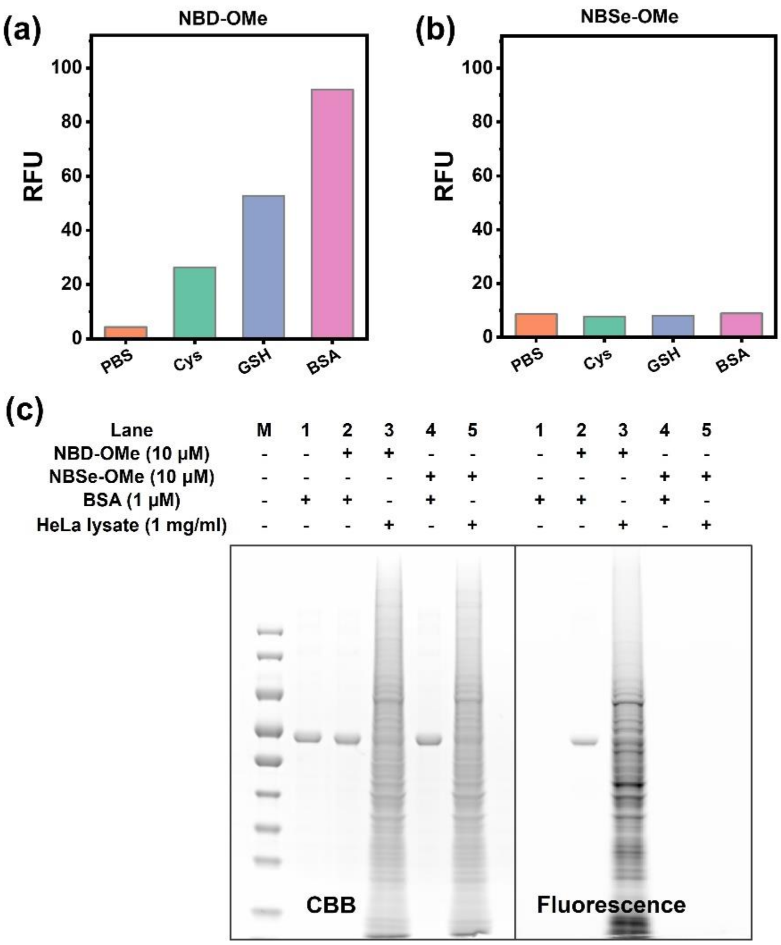

Figure S42 Relative fluorescence intensities of **NBD-OMe** (a) and **NBSe-OMe** (b) incubated with Cys (100 μM), GSH (1 mM) and BSA (1 μM), respectively (incubation condition: 3 hours in PBS 7.4 buffer at 37 ℃). (c) In-gel analysis of **NBD-OMe** and **NBSe-OMe** reacting with BSA and HeLa lysate (incubation condition: 3 hours in PBS 7.4 buffer at 37 ℃).

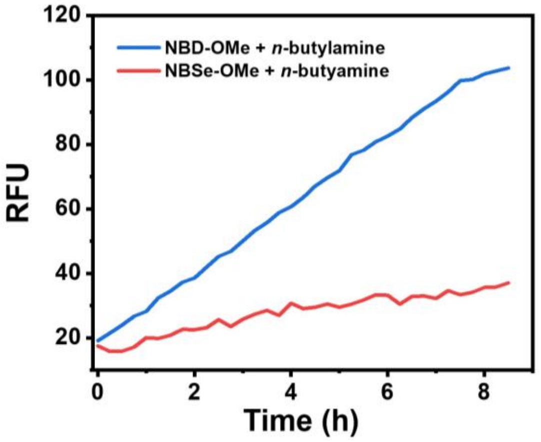

Figure S43 Kinetic study of **NBD-OMe** and **NBSe-OMe** with *n*-butylamine in DMSO at room temperature. Probe concentration: 10 μM; *n*-butylamine concentration: 100 μM.

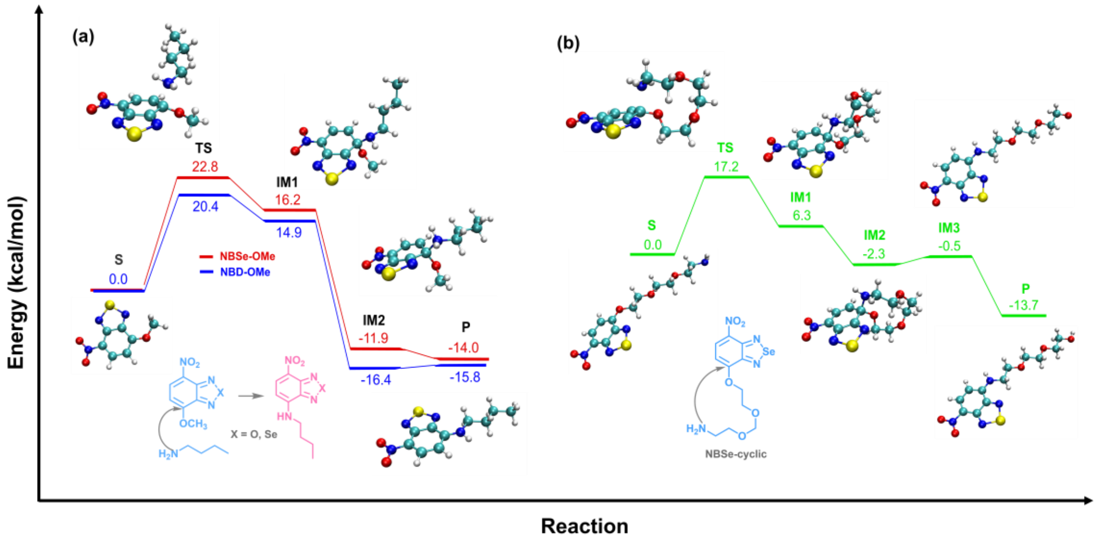

Figure S44 (a) Computed energy profiles, transition state and intermediate structures in the reaction of **NBSe-OMe** (red curve) and **NBD-OMe** (blue curve) with *n*-butylamine, respectively. (b) Computed energy profiles, transition state and intermediate structures in the intramolecular reaction of **NBSe-cyclic**. Gibbs free energies, kcal/mol.

Note: We performed DFT calculation to investigate the reaction mechanism of **NBSe-OMe** and **NBD-OMe** with amine. As depicted in Figure S44a, the addition of *n*-butylamine to the aromatic ring of **NBSe-OMe**/**NBD-OMe** constitutes the rate-determining step in this S_NAr-based reaction (TS in Figure 44a). Following elimination of methoxy group, **NBSe-N-nBu** or **NBD-N-nBu** is formed (P in Figure 44a). The Gibbs free energy changes were calculated to be -14.0 and -15.8 kcal/mol for the reaction of **NBSe-OMe** and **NBD-OMe** with *n*-butylamine, respectively. These findings suggest that the nucleophilic substitution reaction encounters a lower energy barrier and is thermodynamically favored. Notably, the transition state Gibbs free energy for **NBD-OMe**'s reaction (20.4 kcal/mol) with *n*-butylamine was found to be 2.4 kcal/mol lower than that of **NBSe-OMe** (22.8 kcal/mol), signifying a reaction rate approximately 58 times faster[9]. These results illustrated that while both **NBD-OMe** and **NBSe-OMe** can react with amino groups, **NBSe-OMe**'s reactivity is reduced. However, this slower reaction rate can be alleviated by leveraging intramolecular or proximity reactions. For example, the activation energy of the transition state TS1-NBSe-cyclic was determined to be 17.2 kcal/mol (Figure 44b), indicating a low activation energy barrier conducive to the intramolecular transformation from alkoxy-NBSe to amino-NBSe.

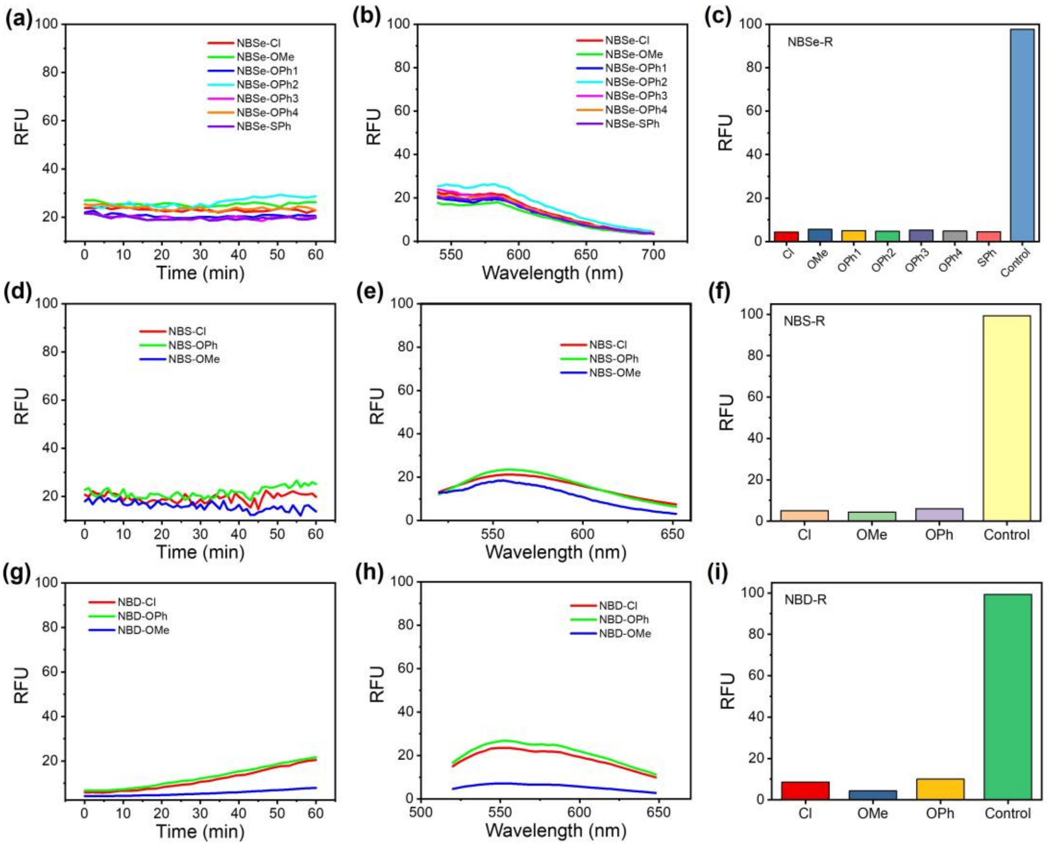

Figure S45 (a-c) Time-dependent fluorescence intensities at 600 nm (a), fluorescence spectra (b) and relative fluorescence intensity at 600 nm (c) of NBSe-R reacting with lysine. The control group in (c) is the reaction of NBSe-Cl with cysteine. λ_{ex} = 490 nm. (d-f) Time-dependent fluorescence intensities at 555 nm (d), fluorescence spectra (e) and relative fluorescence intensity at 555 nm (f) of NBS-R reacting with lysine. The control group in (f) is the reaction of NBS-Cl with cysteine. λ_{ex} = 470 nm. (g-i) Time-dependent fluorescence intensities at 550 nm (g), fluorescence spectra (h) and relative fluorescence intensity at 550 nm (i) of NBD-R reacting with lysine. The control group in (i) is the reaction of NBD-Cl with cysteine. λ_{ex} = 470 nm. Probes' concentration: 10 μM; lysine's concentration: 100 μM; incubation conditions: PBS buffer (pH 7.4, 10 mM, containing 20% DMSO) at room temperature for 60 min.

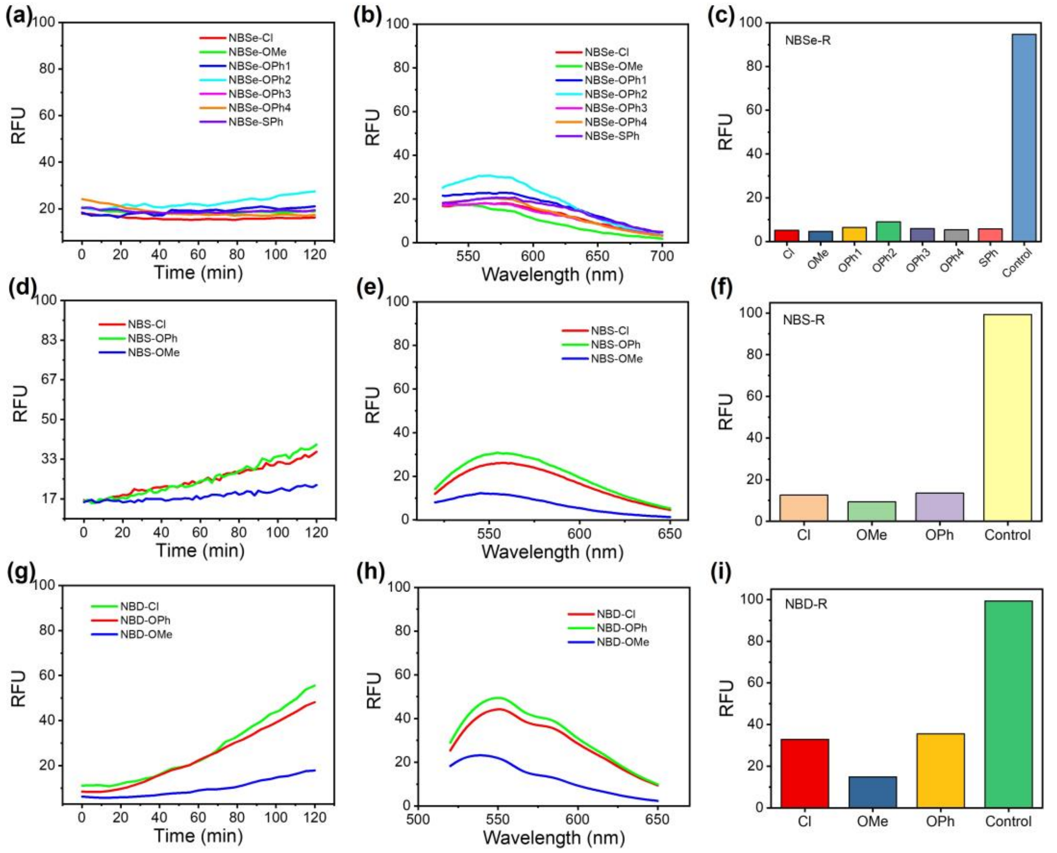

Figure S46 (a-c) Time-dependent fluorescence intensities at 600 nm (a), fluorescence spectra (b) and relative fluorescence intensity at 600 nm (c) of NBSe-R reacting with lysine. The control group in (c) is the reaction of NBSe-Cl with cysteine. λ_{ex} = 490 nm. (d-f) Time-dependent fluorescence intensities at 555 nm (d), fluorescence spectra (e) and relative fluorescence intensity at 555 nm (f) of NBS-R reacting with lysine. The control group in (f) is the reaction of NBS-Cl with cysteine. λ_{ex} = 470 nm. (g-i) Time-dependent fluorescence intensities at 550 nm (g), fluorescence spectra (h) and relative fluorescence intensity at 550 nm (i) of NBD-R reacting with lysine. The control group in (i) is the reaction of NBD-Cl with cysteine. λ_{ex} = 470 nm. Probes' concentration: 10 µM; lysine's concentration: 100 µM; incubation conditions: HEPES buffer (pH 8.0, 10 mM, containing 20% DMSO) at 37 ℃ for 2 h.

9. Supplementary data for HDAC detection

9.1 HPLC analysis on the reaction mixture

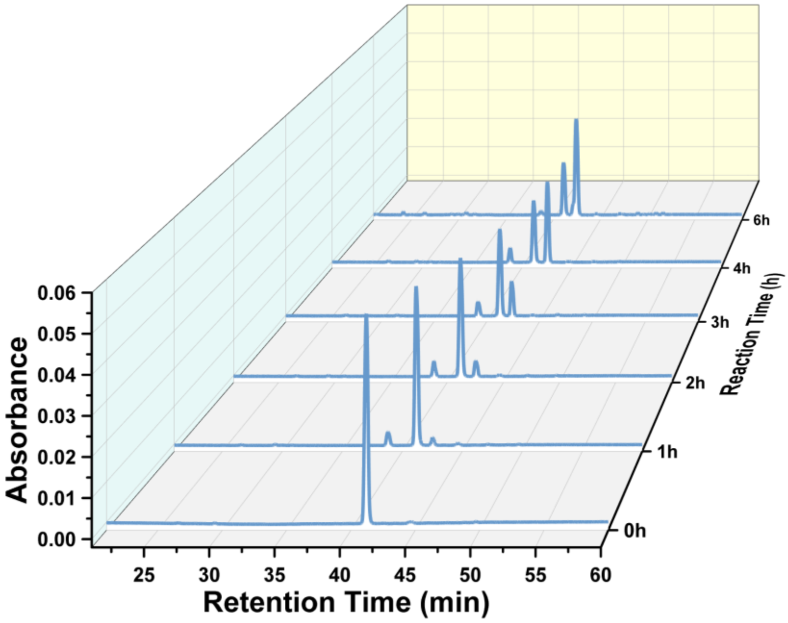

Figure S47 HPLC analysis on the reaction mixture of **NBSe-HDAC** (30 μM) with Sirt1 (0.5 μM) at different time intervals in HEPES buffer (pH 8.0) containing 0.5 mM NAD^+ at 37 °C. Detection wavelength: 330 nm.

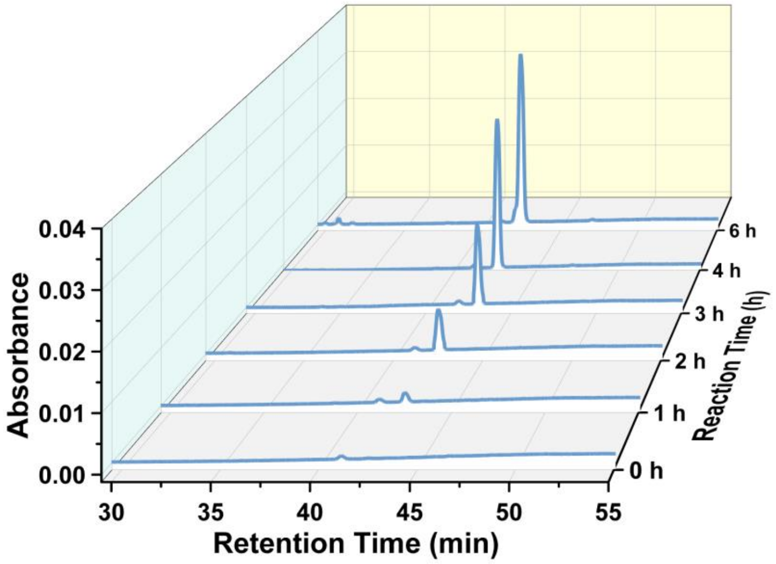

Figure S48 HPLC analysis on the reaction mixture of **NBSe-HDAC** (30 μM) with Sirt1 (0.5 μM) at different time intervals in HEPES buffer (pH 8.0) containing 0.5 mM NAD^+ at 37 °C. Detection wavelength: 500 nm.

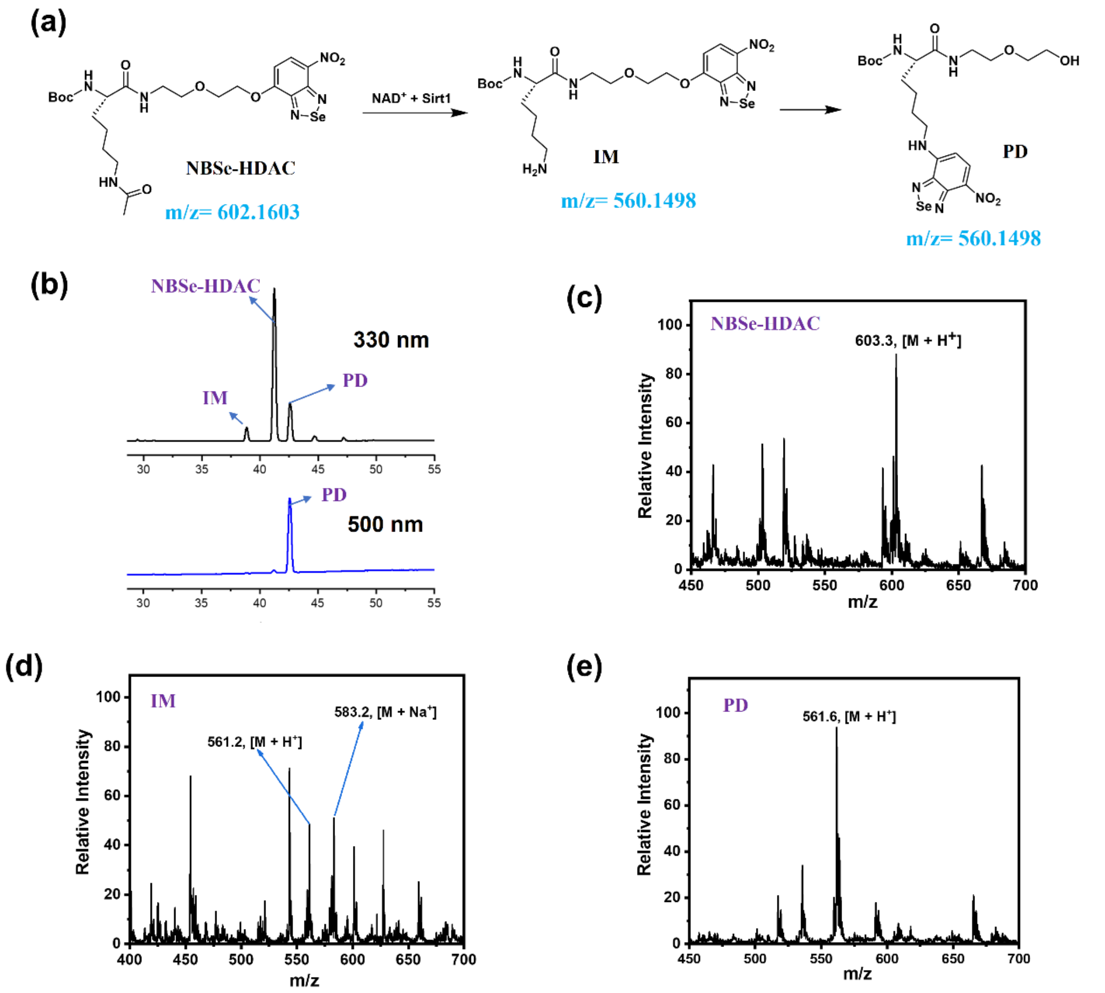

Figure S49 (a) Reaction mechanism study of **NBSe-HDAC** towards Sirt1. (b) HPLC analysis of the reaction mixture of **NBSe-HDAC** with Sirt1 with different detection wavelengths (330 nm and 500 nm). (c-e) Mass analysis of the eluted fraction with retention times of 41.70 min (peak **NBSe-HDAC**), 39.0 min (peak **IM**) and 43.05 min (peak **PD**) respectively. The HPLC assay conditions are as follows: **NBSe-HDAC** concentration: 30 μM; Sirt1 concentration: 1 μM; NAD^+ concentration: 1 mM; incubation conditions: HEPES 8.0 buffer, 37 °C, 3 h.

Note: MS analysis showed that the peak with a retention time of 43.05 min should be assigned to the expected deacetylated/exchanged product, **PD**. The peak with a retention time of 39.0 min corresponded to the expected deacetylated product, **IM**.

9.2 Kinetic parameters of the enzymatic reaction

The kinetic parameters of the enzymatic reaction of Sirt1 towards the **NBSe-HDAC** probe could be determined using a modified Michaelis–Menten equation (Eq-9, Eq-10) by fluorescence method followed the previous protocol published in Kikuchi's work.[10]

$$F_t = F_0 + (F_{max} - F_0)(Ae^{-k_z t} + Bt - A)/[X]_0 \qquad \text{(Eq-9)}$$

$$B = \frac{k_Y[E]_0[X]}{K_m+[X]} \qquad \text{(Eq-10)}$$

Where, F_t: observed fluorescence intensity; F_0: initial fluorescence intensity; F_{max}: maximum fluorescence intensity; A: constant; $[X]_0$: total concentration of the probe; k_z is 0.00734 min^{-1}, which was obtained from the first-order rate constant (*k*) of the model probe **NBSe-MD** (Figure S50); $[E]_0$: concentration of enzyme; [X]: concentration of the probe; k_Y: turnover number (k_{cat}); K_m: Michaelis constant.

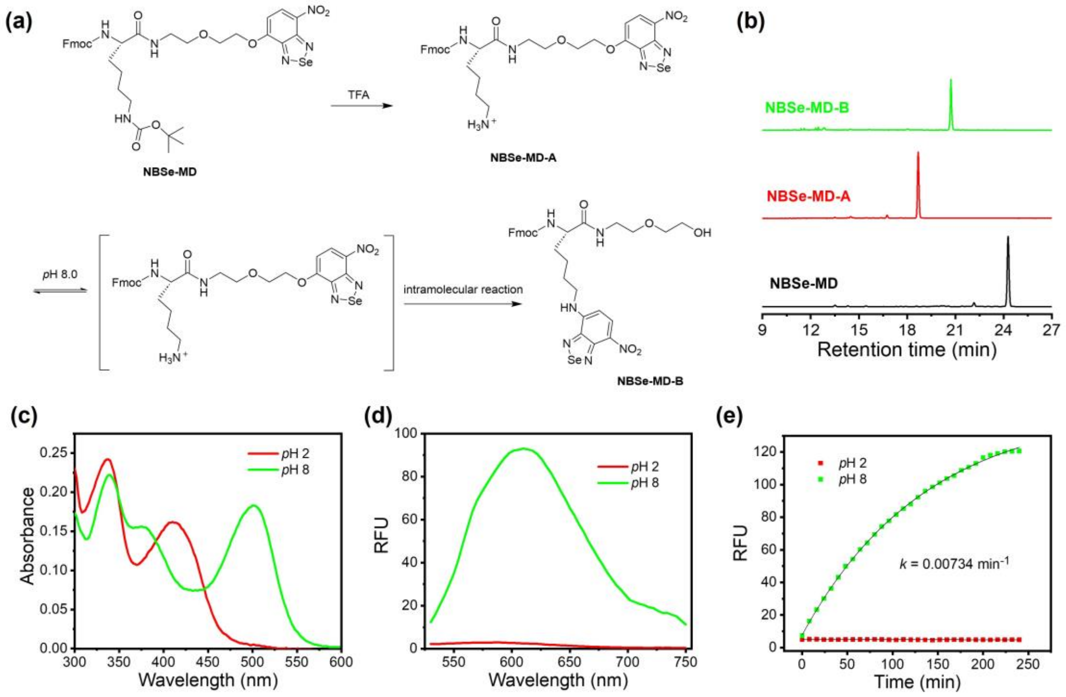

Figure S50 (a) Schematic illustration of the principle of **NBSe-MD** as a model probe. (b) Reversed-phase HPLC analysis of **NBSe-MD-A** at pH 2.0 and pH 8.0. (c) Absorption spectra of **NBSe-MD-A** (10 μM) at pH 2.0 and pH 8.0. (d) Fluorescence spectra of **NBSe-MD-A** (10 μM) at pH 2.0 and pH 8.0 (λ_{ex} = 490 nm). (e) Time-dependent fluorescence measurement of **NBSe-MD-A** at pH 2.0 and pH 8.0 (λ_{ex} = 490 nm; λ_{em} = 600 nm). The first-order rate constant *k* of **NBSe-MD-A** was determined to be 0.00734 min^{-1}.

Generally, we first measured the time-dependent fluorescence increase of **NBSe-HDAC** with Sirt1 at different probe concentrations. Through equation Eq-9, we can derive *B* values at different probe concentrations (Figure 51a). Subsequently we fitted these data using equation Eq-10 to derive k_{cat} and K_m values (Figure 51b). The k_{cat} and K_m values were found to be 4.8×10^{-1} s^{-1} and 1.61×10^{-4} M, respectively. The reactivity of Sirt1 towards substrate **NBSe-HDAC** was $k_{cat}/K_m = 2.99 \times 10^3$ $M^{-1}s^{-1}$. This value is similar to the reported values of H3K9Ac peptide substrates[11].

(a)

Concentration (μM)	*B* (μM min^{-1})
2	0.0205 ± 0.00279
4	0.0409 ± 0.00401
6	0.09516 ± 0.00568
8	0.12027 ± 0.00679
10	0.16961 ± 0.00917
15	0.25706 ± 0.01097
20	0.34735 ± 0.01442
25	0.40725 ± 0.01674
30	0.4507 ± 0.02139
35	0.50998 ± 0.01464

(b)

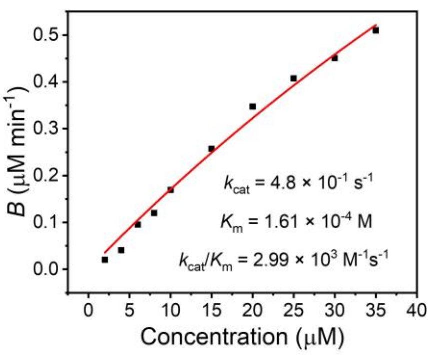

Figure S51 (a) *B* values obtained using various concentrations of **NBSe-HDAC** probe with Sirt1 by fitting equation Eq-9. (b) *B* values were plotted against various concentrations of **NBSe-HDAC** to determine turnover number (k_{cat}) and Michaelis constant (K_m) by fitting equation Eq-10.

9.3 Selectivity analysis of NBSe-HDAC probe

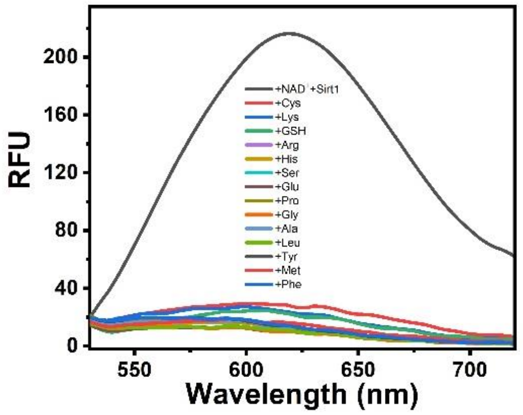

Figure S52. Fluorescence spectra of **NBSe-HDAC** (10 μM) in response to different analytes (100 μM) after 5 h incubation. λ_{ex} = 490 nm.

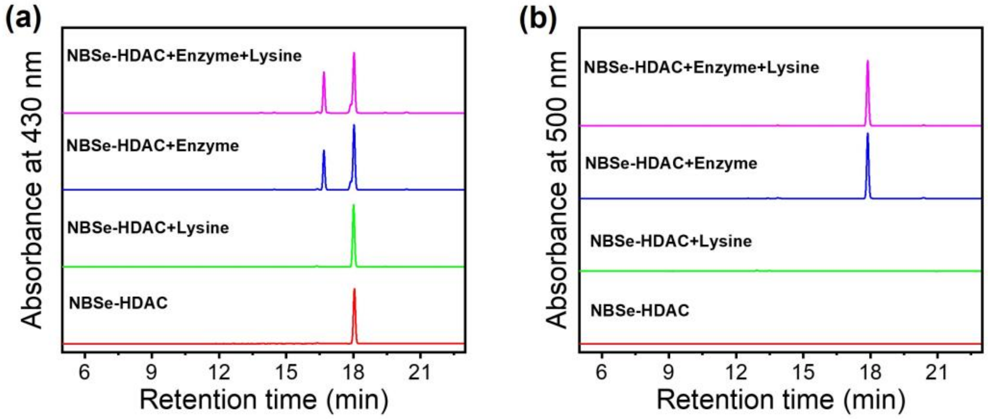

Figure S53. Reversed-phase HPLC analysis of the reaction mixture of **NBSe-HDAC** with enzyme or lysine under detection wavelengths (a) 430 nm and (b) 500 nm. The HPLC assay conditions are as follows: **NBSe-HDAC** concentration: 20 μM; Sirt1 concentration: 0.5 μM; NAD^+ concentration: 0.5 mM; lysine concentration: 1 mM. incubation conditions: HEPES 8.0 buffer, 37 °C, 3 h.

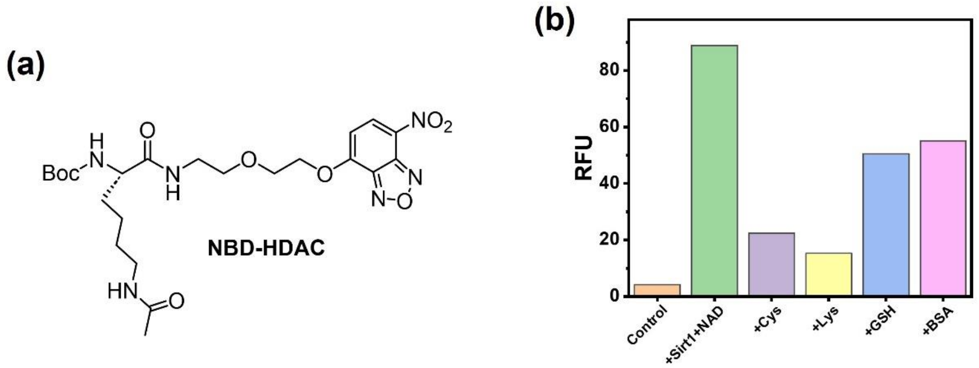

Figure S54. (a) The structure of probe **NBD-HDAC**. (b) Relative fluorescence intensity at 550 nm of **NBD-HDAC** towards various analytes. **NBD-HDAC** concentration: 10 μM; Sirt1 concentration: 0.1 μM; NAD^+: 200 μM; Cys: 100 μM, Lys: 100 μM; GSH: 1 mM; BSA: 1 μM. Incubation conditions: HEPES 8.0 buffer, 37 °C, 3 h.

10.Supplementary data for HaloTag detection

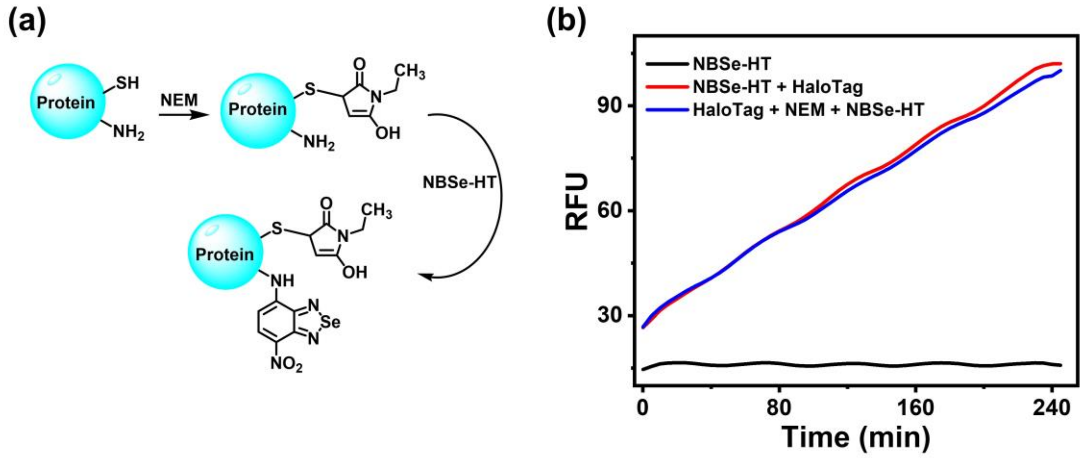

Figure S55 (a) Response mechanism of HaloTag protein towards NEM and **NBSe-HT**. (b) Kinetic study of **NBSe-HT** in response to HaloTag in the absence or presence of NEM in HEPES buffer.

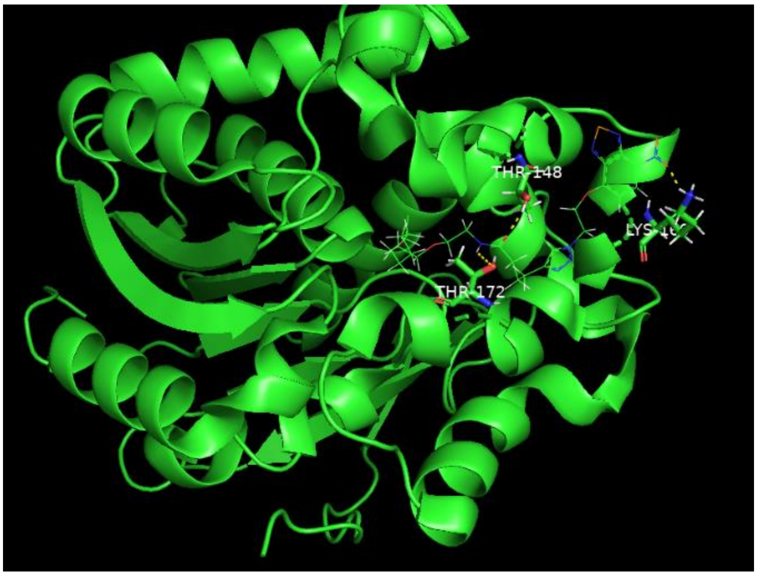

Figure S56 Docking experiments for analyzing the binding model of **NBSe-HT** with HaloTag7 (PDB code: 6Y7A).

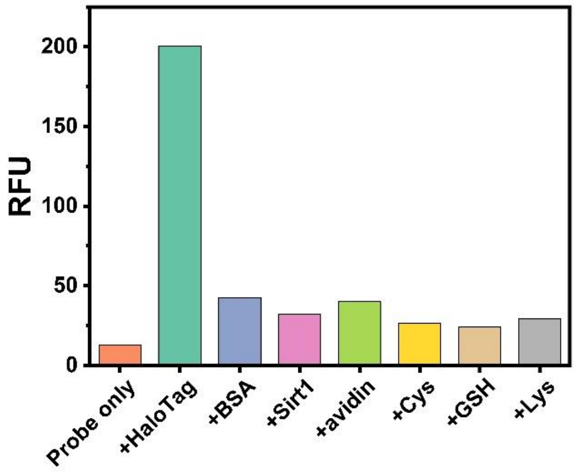

Figure S57 Fluorescence responses of **NBSe-HT** (10 μM) towards various biological analytes (HaloTag: 2 μM; BSA: 1 μM; Sirt1: 1 μM; avidin: 1 μM; Cys: 100 μM; GSH: 1 mM; Lys: 100 μM), respectively in HEPES buffer (pH 8.0) at 37 °C. Incubation time: 3 hours. λ_{ex} = 500 nm, λ_{em} = 600 nm.

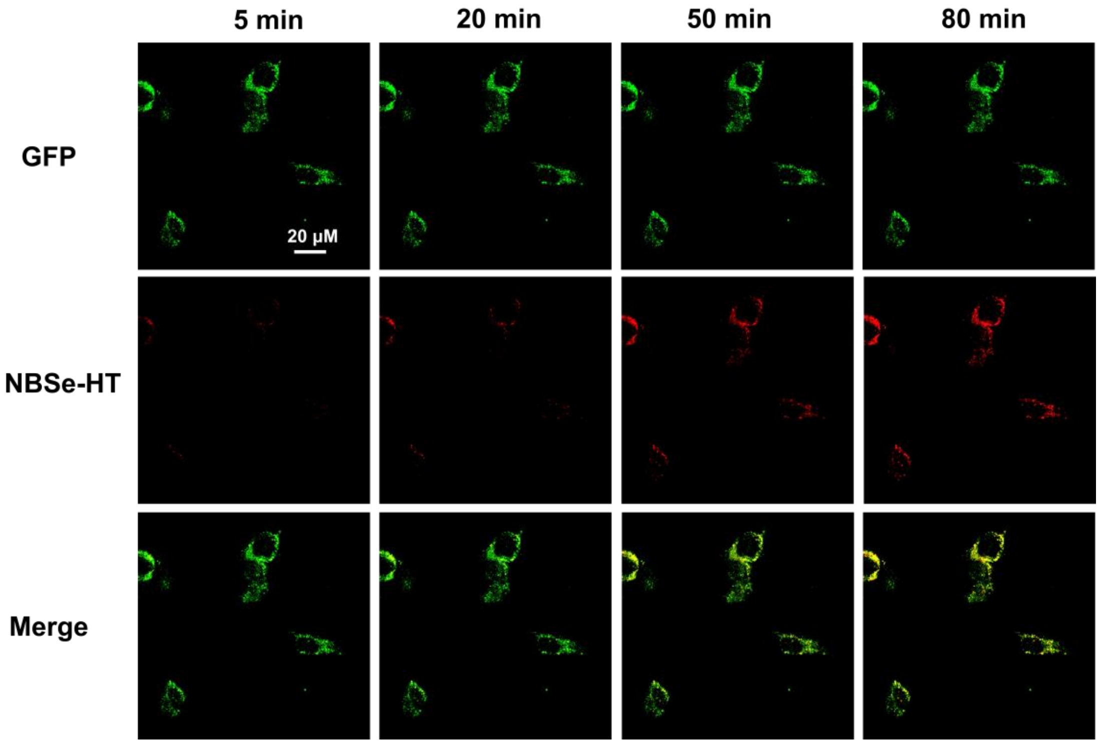

Figure S58. Confocal fluorescence images of HeLa cells transfected with HaloTag-GFP-Mito at different time points after incubation with **NBSe-HT** (10 μM). Emission signals were collected at 500-530 nm for the GFP channel (green) and 590-650 nm for the **NBSe-HT** channel (red). λ_{ex} = 488 nm.

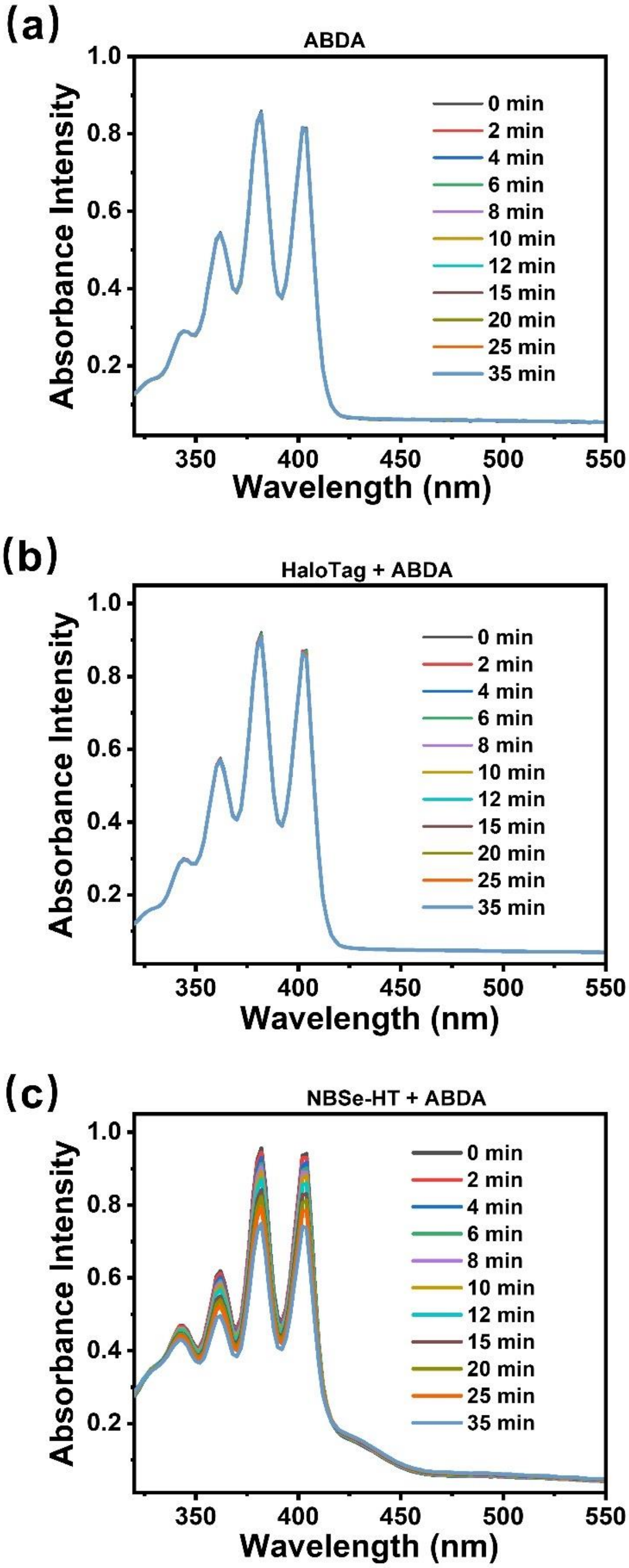

Figure S59. Absorption spectra of ABDA (5 μM) under different conditions in HEPES 8.0 buffer with light irradiation. (a) Blank; (b) HaloTag (5 μM); and (c) **NBSe-HT** (10 μM). LED light: 490-500 nm, 30 mW/cm^2.

11. Chemical synthesis

Synthesis of NBSe-Cl/F

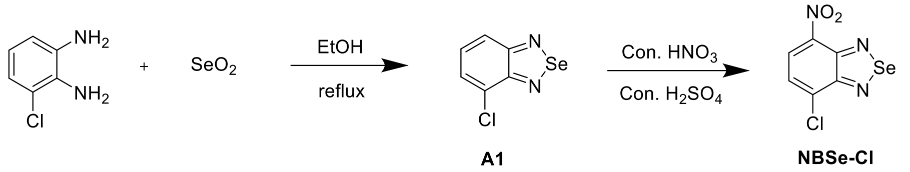

To the solution of 3-chlorobenzene-1,2-diamine (1 g, 7.04 mmol) in 40 mL anhydrous ethanol was added SeO_2 (959 mg, 8.56 mmol). The mixture was heated to reflux for 2 h. Then, the solvent was removed under vacuum. The residue was purified by silica gel column chromatograph (eluent: petroleum ether (PE) and ethyl acetate (EA)) to give 1.31 g compound **A1** as a yellow solid, yield 87%.

Under an ice bath, to the solution of compound **A1** (1.31g) in 30 mL of concentrated H_2SO_4 was added 7 mL concentrated HNO_3. After stirring at 0 ˚C for 1 h, the reaction mixture was slowly poured into 200 mL cold water. The yellow precipitate was collected by filtration, washed with water, and purified by silica gel column chromatography (eluent: dichloromethane (DCM)) to afford 1.35 g of **NBSe-Cl** as a pale yellow solid, yield: 89%. ^{1}H NMR (400 MHz, DMSO-d_6) δ 8.49 (d, J = 8.0 Hz, 1H), 7.93 (d, J = 8.0 Hz, 1H). ^{13}C NMR (100 MHz, DMSO-d_6) δ 157.26, 150.56, 140.31, 133.65, 127.84, 126.47. HRMS (ESI) m/z for $C_6HClN_3O_2Se$ $[M-H]^-$, calcd.: 261.8928, found: 261.8912.

NBSe-F was prepared in a similar procedure of **NBSe-Cl**. Yield: 76%. ^{1}H NMR (400 MHz, DMSO-d_6) δ 8.60 (dd, J = 8.4, 4.7 Hz, 1H), 7.54 (dd, J = 9.6, 8.5 Hz, 1H). ^{13}C NMR (101 MHz, DMSO-d_6) δ 157.84, 155.54, 152.29, 151.47, 129.65, 109.90. HRMS (ESI) m/z for $C_6H_3FN_3O_2Se$ $[M+H]^+$, calcd.:247.9369, found: 247.9394.

Synthesis of NBSe-N-nBu

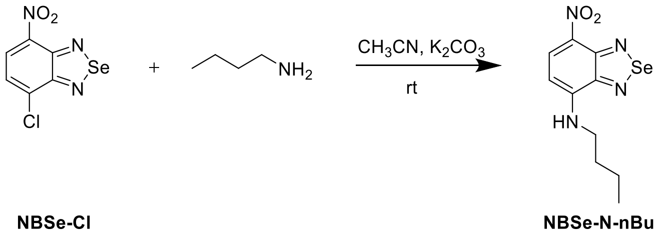

A mixture of **NBSe-Cl** (30 mg, 0.11 mmol), *n*-butylamine (13 mg, 0.23 mmol) and K_2CO_3 (48 mg, 0.34 mmol) in 3 mL of acetonitrile was stirred at room temperature for 4 h. Then, the reaction mixture was poured into 50 mL of water and the obtained mixture was extracted with dichloromethane (DCM) three time (3 x 30 mL). The organic layers were separated, combined, washed with H_2O, and dried over anhydrous $MgSO_4$. The solvent was removed under vacuum to give a residue, which was purified by silica gel column chromatograph (PE: EA = 50: 1, v/v) to afford 17 mg **NBSe-N-nBu** as a red solid, yield 50%. ^{1}H NMR (400 MHz, DMSO-d_6) δ 8.75 – 8.42 (m, 2H), 6.38 (d, J = 9.1 Hz, 1H), 3.62 (s, 2H), 1.74 – 1.55 (m, 2H), 1.37 (dt, J = 14.5, 7.3 Hz, 2H), 0.93 (t, J = 7.4 Hz, 3H). ^{13}C NMR (100 MHz, DMSO-d_6) δ 152.55, 152.41, 149.03, 135.87, 128.29, 97.67, 55.46, 30.50, 20.15, 14.22. HRMS (ESI) m/z for $C_{10}H_{13}N_4O_2Se$ $[M+H]^+$, calcd.:301.0198, found: 301.0135.

Synthesis of NBSe-S-nBu

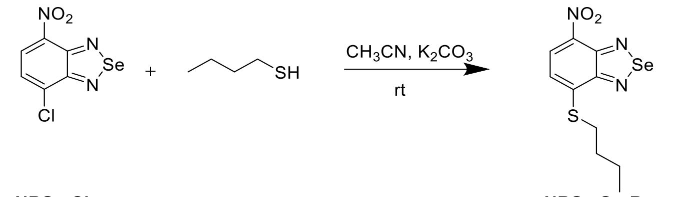

NBSe-S-nBu was prepared in a similar method for the synthesis of **NBSe-N-nBu**. Yield 56%. ^{1}H NMR (400 MHz, DMSO-*d₆*) δ 8.48 (d, J = 8.0 Hz, 1H), 7.40 (d, J = 8.0 Hz, 1H), 3.23 (t, J = 7.8 Hz, 2H), 1.82 – 1.67 (m, 2H), 1.58 – 1.45 (m, 2H), 0.95 (t, J = 7.5 Hz, 3H). ^{13}C NMR (100 MHz, DMSO-d_6) δ 155.32, 150.62, 144.64, 139.02, 128.87, 119.02, 30.42, 29.71, 20.51, 14.68. HRMS (ESI) m/z $C_{11}H_{12}N_3O_2SSe$ $[M+H]^+$, calcd.:317.9810, found: 317.9748.

Synthesis of compound A2

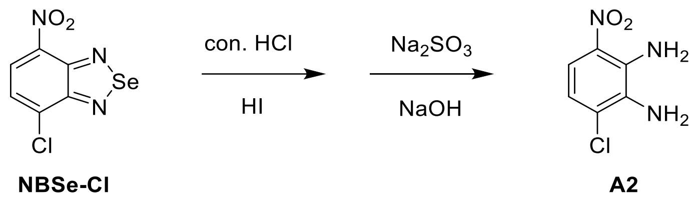

To the solution of **NBSe-Cl** (263 mg, 1.00 mmol) in 10 mL concentrated hydrochloric caid was added 2.5 mL of 57 % aqueous HI under an ice bath. After stirring at room temperature for 1 hour, 10 mL of saturated Na_2SO_3 solution was slowly added to the reaction mixture. Then, the reaction solution was basified with 2 M aqueous NaOH solution to pH 8.0. The aqueous mixture was extracted with DCM three time (3 x 50 mL). The organic layers were separated, combined, washed with H_2O, and filtered through diatomite. The organic solvent was removed by evaporation under vacuum to give 177 compound **A2** as a red solid, yield 95%. Compound **A2** was directly used for the next reaction without further purification.

Synthesis of NBS-Cl

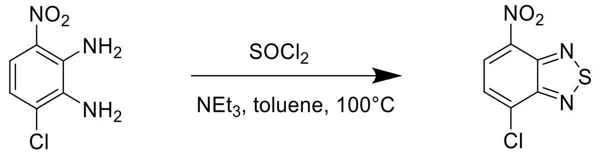

To a solution of compound **A2** (150 mg, 0.80 mmol) and triethylamine (540 mg) in 6 mL toluene, was added $SOCl_2$ (800 mg in 2 mL toluene) dropwise. The reaction mixture was heated at 100 °C for 2 h. Then, the solvent was removed under vacuum, and the residue was purified by silica gel column chromatography (DCM: PE = 1: 30, v/v) to afford 100 mg **NBS-Cl** as a white solid, yield 58%. ^{1}H NMR (400 MHz, DMSO-d_6) δ 8.68 (d, *J* = 8.2 Hz, 1H), 8.11 (d, *J* = 8.2 Hz, 1H). ^{13}C NMR (100 MHz, DMSO-d_6) δ 156.56, 146.61, 138.66, 133.61, 129.69, 128.04. HRMS (ESI) m/z for $C_6H_2ClN_3O_2SNa$ $[M+Na]^+$, calcd.: 237.9448, found: 237.9455.

Synthesis of NBS-N-nBu

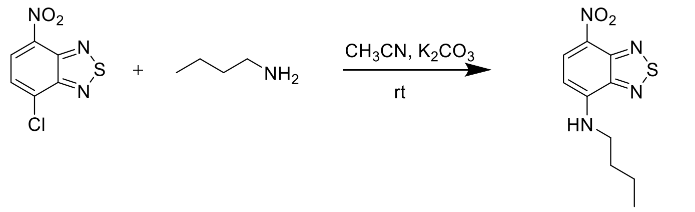

NBS-N-nBu was prepared in a similar procedure for **NBSe-N-nBu**. Yield 60%. ^{1}H NMR (400 MHz, DMSO-d_6) δ 8.78 (t, J = 6.2 Hz, 1H), 8.61 (d, J = 9.1 Hz, 1H), 6.58 (d, J = 9.1 Hz, 1H), 1.66 (p, J = 7.1 Hz, 2H), 1.40 (p, J = 7.3 Hz, 2H), 0.93 (t, J = 7.3 Hz, 3H). ^{13}C NMR (100 MHz, DMSO-d_6) δ 152.54, 152.39, 149.02, 135.88, 128.28, 97.91, 43.02, 30.49, 20.09, 14.21. HRMS (ESI) m/z for $C_{10}H_{12}N_4O_2SNa$ $[M+Na]^+$, calcd.: 275.0573, found: 275.0571.

Synthesis of NBS-S-nBu

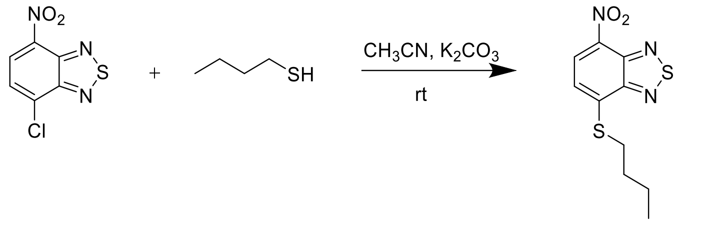

The preparation of **NBS-S-nBu** was performed according to the procedure for **NBSe-N-nBu**. Yield 80%. ^{1}H NMR (400 MHz, DMSO-d_6) δ 8.62 (d, J = 8.2 Hz, 1H), 7.61 (d, J = 8.2 Hz, 1H), 3.30 (t, J = 7.4 Hz, 2H), 1.75 (p, J = 7.4 Hz, 2H), 1.52 (p, J = 7.4 Hz, 2H), 0.95 (t, J = 7.4 Hz, 3H). ^{13}C NMR (100 MHz, DMSO-d_6) δ 153.84, 146.71, 144.13, 136.26, 129.73, 120.67, 30.44, 30.09, 20.79, 14.39. HRMS (ESI) m/z for $C_{10}H_{11}N_3O_2S_2Na$ $[M+Na]^+$, calcd.: 292.0185, found: 292.0181.

Synthesis of NBSe-OMe

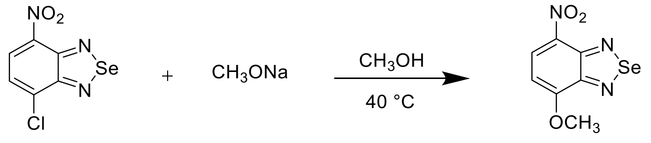

A solution of **NBSe-Cl** (100 mg, 0.38 mmol) and $NaOCH_3$ (41 mg, 0.76 mmol) in 5 mL anhydrous methanol was heated at 40 °C for 3 h. The solvent was removed to give a residue, which was purified by silica gel column chromatography (DCM: EA = 50: 1, v/v) to afford 54 mg **NBSe-OMe** as a pale-yellow solid, yield 58%. ^{1}H NMR (400 MHz, DMSO-d_6) δ 8.66 (d, J = 8.5 Hz, 1H), 6.99 (d, J = 8.6 Hz, 1H), 4.13 (s, 3H). ^{13}C NMR (100 MHz, DMSO-d_6) δ 157.17, 153.87, 152.02, 135.13, 132.21, 103.32, 57.21. HRMS (ESI) m/z for $C_7H_5N_3O_3SeNa$ $[M+Na]^+$, calcd.: 281.9388, found: 281.9389.

Synthesis of NBSe-OPh1

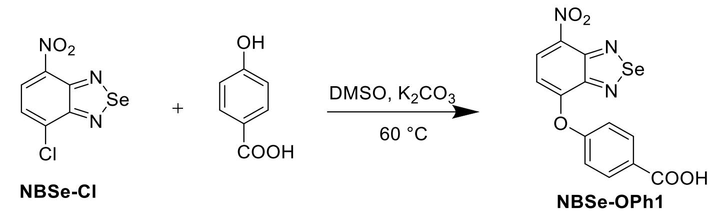

NBSe-Cl **NBSe-OPh1**

A suspension of **NBSe-Cl** (100 mg, 0.38 mmol), *p*-hydroxybenzoic acid (79 mg, 0.57 mmol) and K_2CO_3 (105 mg, 0.76 mmol) in 4 mL DMSO was heated at 60 °C for 6 h. After the removal of solvent, the obtained residue was purified by silica gel column chromatography (PE: DCM = 50: 1, v/v) to afford 46 mg **NBSe-OPh1** as a yellow solid. Yield: 33%. ^{1}H NMR (400 MHz, DMSO-d_6) δ 8.58 (d, J = 8.4 Hz, 1H), 8.04 (d, J = 8.6 Hz, 2H), 7.35 (d, J = 8.6 Hz, 2H), 7.01 (d, J = 8.4 Hz, 1H). ^{13}C NMR (100 MHz, DMSO-d_6) δ 167.12, 160.79, 158.83, 153.72, 153.63, 152.34, 137.00, 132.31, 130.48, 119.89, 111.17. HRMS (ESI) m/z for $C_{13}H_6N_3O_5Se$ [M-H]$^-$, calcd.: 363.9478, found: 363.9283.

Synthesis of NBSe-OPh2

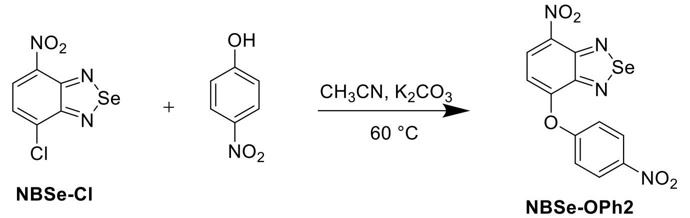

NBSe-Cl **NBSe-OPh2**

Compound **NBSe-OPh2** was obtained in the same synthetic method for compound **NBSe-OPh1**. Yield: 55%. ^{1}H NMR (400 MHz, DMSO-d_6) δ 8.60 (d, J = 8.3 Hz, 1H), 8.35 – 8.26 (m, 2H), 7.47 – 7.39 (m, 2H), 7.31 (d, J = 8.3 Hz, 1H). ^{13}C NMR (100 MHz, DMSO-d_6) δ 60.38, 153.67, 152.42, 151.34, 144.14, 138.13, 130.18, 125.67, 119.72, 114.00. HRMS (ESI) m/z for $C_{12}H_6N_4O_5Se$ [M-H]$^-$, calcd.: 364.9420, found: 364.9243.

Synthesis of NBSe-OPh3

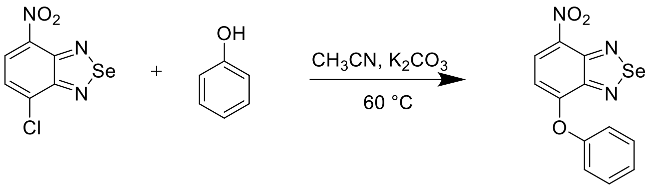

NBSe-Cl **NBSe-OPh3**

Compound **NBSe-OPh3** was obtained in the same synthetic method for compound **NBSe-OPh1**. yield: 44%. ^{1}H NMR (400 MHz, DMSO-d_6) δ 8.56 (d, J = 8.5 Hz, 1H), 7.59 – 7.52 (m, 2H), 7.40 – 7.31 (m, 3H), 6.69 (d, J = 8.4 Hz, 1H). ^{13}C NMR (100 MHz, DMSO-d_6) δ 155.72, 154.89, 153.51, 152.31, 135.89, 131.14, 131.07, 126.43, 121.04, 108.06. HRMS (ESI) m/z for $C_{12}H_8N_3O_3Se$ [M+H]$^+$, calcd.: 321.9725, found: 321.9669.

Synthesis of NBSe-OPh4

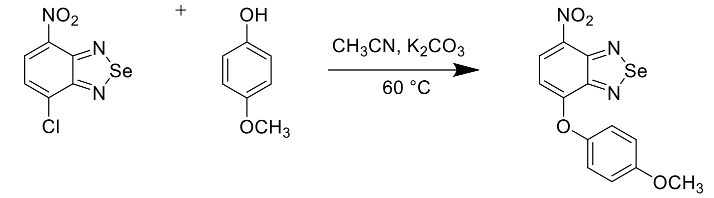

NBSe-Cl **NBSe-OPh4**

Compound **NBSe-OPh4** was obtained in the same synthetic method for compound **NBSe-OPh1**. Yield: 51%. ^{1}H NMR (400 MHz, DMSO-d_6) δ 8.55 (d, J = 8.5 Hz, 1H), 7.33 – 7.25 (m, 2H), 7.14 – 7.07 (m, 2H), 6.58 (d, J = 8.5 Hz, 1H), 3.81 (s, 3H). ^{13}C NMR (100 MHz, DMSO-d_6) δ 158.35, 156.80, 153.35, 152.26, 147.29, 135.40, 131.41, 122.48, 115.97, 106.74, 56.01. HRMS (ESI) m/z for $C_{13}H_{10}N_3O_4Se$ $[M+H]^+$, calcd.: 351.9831, found: 351.9772.

Synthesis of NBSe-SPh

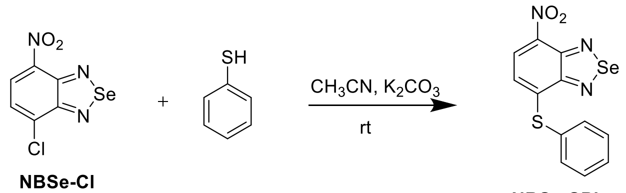

NBSe-Cl **NBSe-SPh**

A mixture of **NBSe-Cl** (100 mg, 0.38 mmol), thiophenol (63 mg, 0.57 mmol) and K_2CO_3 (105 mg, 0.76 mmol) in 6 mL anhydrous acetonitrile was stirred at room temperature for 2 h. After the removal of solvent, the residue was purified by silica gel column chromatography (DCM: EA = 50: 1, v/v) to afford 59 mg **NBSe-SPh** as a dark-yellow solid. Yield: 46%. ^{1}H NMR (400 MHz, DMSO-d_6) δ 8.39 (d, J = 8.1 Hz, 1H), 7.73 (s, 2H), 7.65 (s, 3H), 6.68 (d, J = 8.0 Hz, 1H). ^{13}C NMR (100 MHz, DMSO-d_6) δ 156.90 (s), 150.31 (s), 145.38 (s), 137.75 (s), 135.96 (s), 131.08 (s), 128.82 (s), 128.13 (s), 119.86 (s). HRMS (ESI) m/z for $C_{13}H_7N_3O_5SeNa$ $[M+Na]^+$, calcd.: 337.9497, found: 337.9624.

Synthesis of B2

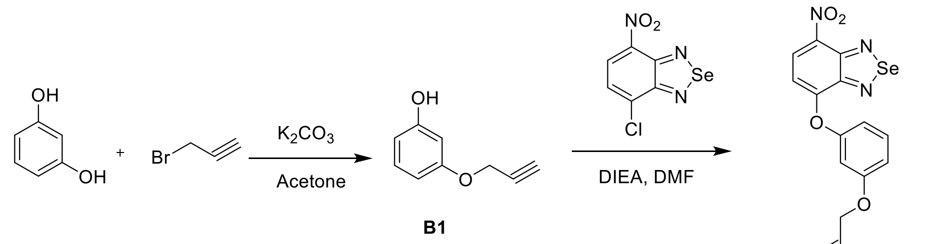

B1 **B2**

Resorcinol (550 mg, 5 mmol), propargyl bromide (570 mg, 4.9 mmol) and K_2CO_3 (1.38 g, 10 mmol) were placed in 30 mL acetone. The mixture was refluxed overnight. Then, the solvent was removed under vacuum to give a residue, which was purified by silica gel column chromatograph (petroleum ether (PE) and ethyl acetate (EA) mixture as eluent) to give 490 mg compound **B1** white solid, yield 67.6%.

The solution of **NBSe-Cl** (50 mg, 0.2 mmol), compound **B1** (45 mg, 0.3 mmol) and DIEA (52

mg, 0.4 mmol) in 5 mL DMF was stirred at room temperature for 6 h. Then, the reaction solution was poured into 30 mL of water. The obtained aqueous solution was extracted with DCM three times (3 x 30 mL). The organic layers were separated, collected and dried over anhydrous Na_2SO_4. The organic solvent was evaporated under vacuum to give a residue, which was purified by silica gel column chromatograph (PE: DCM = 10: 1, v/v) to afford 35 mg compound **B2** as a yellow solid, yield 46.7%. ^{1}H NMR (400 MHz, $CDCl_3$) δ 8.54 (d, J = 8.5 Hz, 1H), 7.49 – 7.41 (m, 1H), 7.01 (dd, J = 8.3, 1.8 Hz, 1H), 6.91 (dd, J = 4.1, 1.7 Hz, 2H), 6.69 (d, J = 8.5 Hz, 1H), 4.75 (d, J = 2.4 Hz, 2H), 2.57 (t, J = 2.3 Hz, 1H). ^{13}C NMR (100 MHz, $CDCl_3$) δ 159.22, 156.14, 154.44, 153.78, 152.63, 135.57, 131.07, 113.86, 113.09, 108.09, 106.83, 77.84, 76.13, 56.07. HRMS (ESI) m/z for $C_{15}H_{10}N_3O_4Se$ $[M+H]^+$, calcd.: 375.9831, found:375.9859.

Synthesis of NBSe-cRGD

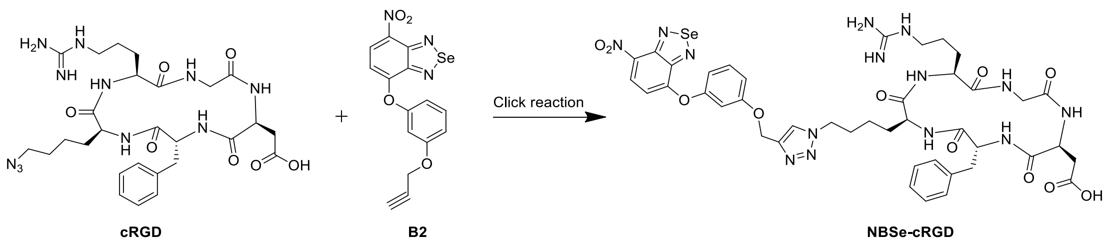

To the solution of **cRGD** (6.3 mg, 0.01 mmol) in 1 mL water was added compound **B2** (7.5 mg, 0.02 mmol in 1 mL DMSO). The mixture was then added to an aqueous solution (4 mL) of $CuSO_4$ (13 mg, 0.05 mmol), THPTA (26 mg, 0.06 mmol), and sodium ascorbate (20 mg, 0.1 mmol). The resulting mixture was stirred at room temperature for 6 h. Pure **NBSe-cRGD** was obtained through preparative HPLC and its chemical structure was confirmed by mass spectrometry. HRMS (ESI) m/z for $C_{42}H_{49}N_{14}O_{11}Se$ $[M+H]^+$, calcd.: 1005.2865, found:1005.2806.

Synthesis of NBSe-HDAC

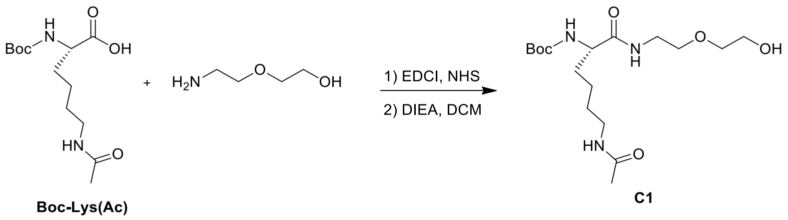

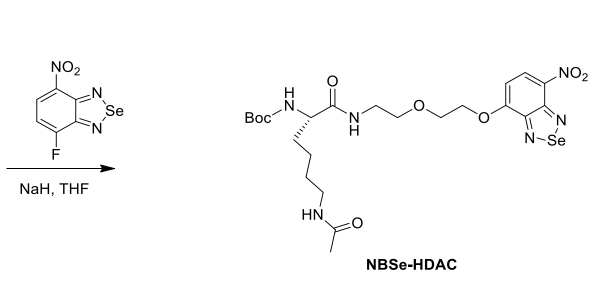

Compound **C1** was synthesized from Boc-Lys(Ac) and 2-aminoethanol through a condensation reaction according to the literature method.[12]

At room temperature, to the solution of compound **C1** (75 mg, 0.2 mmol) in 20 mL of anhydrous THF was added 4.8 mg NaH (60% in oil, 0.12 mmol). The reaction mixture was stirred for 10 min, then **NBSe-F** (37 mg, 0.15 mmol) was added. The mixture was stirred at room temperature overnight, and was then poured into 30 mL of ice water. The aqueous solution was extracted with DCM three times (3 x 50 mL). The organic layers were collected and dried over Na_2SO_4. The solvent was removed by distillation and the residue was purified by silica gel column chromatograph (DCM: MeOH = 50: 1, v/v) to give 32 mg compound **NBSe-HDAC** as a yellow solid. Yield: 35%. ^{1}H NMR (400 MHz, DMSO-d_6) δ 8.65 (d, J = 8.6 Hz, 1H), 7.96 (dd, J = 10.0, 4.3 Hz, 1H), 7.88 (t, J = 5.4 Hz, 1H), 7.02 (d, J = 8.6 Hz, 1H), 6.81 (d, J = 8.2 Hz, 1H), 4.49 – 4.42 (m, 2H), 3.92 – 3.87 (m, 2H), 3.86 – 3.80 (m, 1H), 3.54 – 3.50 (m, 2H), 3.24 (ddd, J = 19.5, 13.5, 6.9 Hz, 2H), 2.98 – 2.91 (m, 2H), 1.77 (s, 3H), 1.56 – 1.41 (m, 2H), 1.36 (d, J = 8.3 Hz, 9H), 1.29 (s, 2H), 1.19 (s, 2H). HRMS (ESI) m/z for $C_{23}H_{34}N_6O_8SeNa$ $[M+Na]^+$, calcd.: 625.1496, found: 625.1690.

Synthesis of NBSe-MD

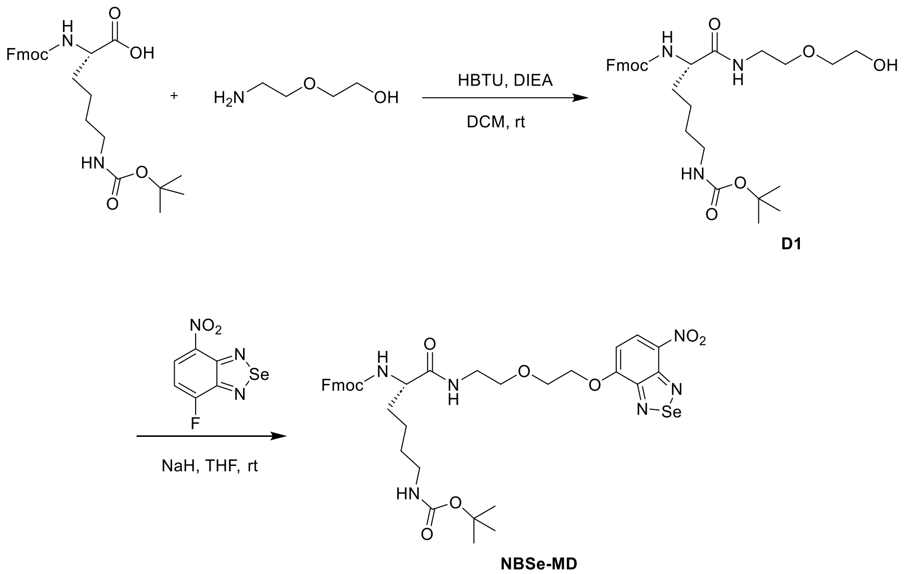

Compound **D1** was synthesized from Fmoc-Lys(Boc) and 2-aminoethanol through a condensation reaction according to the literature method.[12]

At room temperature, to the solution of compound **D1** (110 mg, 0.2 mmol) in 20 mL of anhydrous THF was added 4.8 mg NaH (60% in oil, 0.12 mmol). The reaction mixture was stirred for 10 min, then **NBSe-F** (37 mg, 0.15 mmol) was added. The mixture was stirred at room temperature for 5 hours and was then poured into 30 mL of ice water. The aqueous solution was extracted with DCM three times (3 x 50 mL). The organic layers were collected and dried over Na_2SO_4. The solvent was removed by distillation and the residue was purified by silica gel column chromatograph (DCM: MeOH = 100: 1, v/v) to give 15 mg compound **NBSe-MD** as a yellow solid. Yield: 13%. ^{1}H NMR (400 MHz, $CDCl_3$) δ 8.56 (d, J = 8.5 Hz, 1H), 7.75 (dd, J = 7.3, 4.2 Hz, 2H), 7.63 – 7.56 (m, 2H), 7.43 – 7.38 (m, 2H), 7.33 (dd, J = 6.7, 3.9 Hz, 2H), 6.65 (d, J = 8.6 Hz, 1H), 5.77 (d, J = 5.5 Hz, 1H), 4.39 (d, J = 6.6 Hz, 2H), 4.20 (t, J = 7.1 Hz, 1H), 3.99 (s, 2H), 3.74 – 3.72 (m, 4H), 3.54 (d, J = 16.6 Hz, 2H), 3.21 – 3.18 (m, 4H), 3.07 (d, J = 6.8 Hz, 1H), 1.36 (d, J = 5.7 Hz, 2H), 1.28 (s, 9H), 0.87 (d, J = 2.4 Hz, 2H). ^{13}C NMR (100 MHz, DMSO-d_6) δ 172.50, 162.78, 157.72, 156.39, 153.51,

152.03, 141.17, 134.78, 132.20, 128.28, 128.10, 127.83, 127.53, 125.80, 120.54, 103.33, 77.80, 72.60, 69.42, 66.06, 60.65, 57.68, 54.07, 47.14, 42.32, 36.25, 31.24, 28.75, 23.31, 18.56, 17.20, 12.97. HRMS (ESI) m/z for $C_{36}H_{42}N_6O_9SeNa$ $[M+Na]^+$, calcd.: 805.2071, found: 805.1927.

Synthesis of NBSe-HT

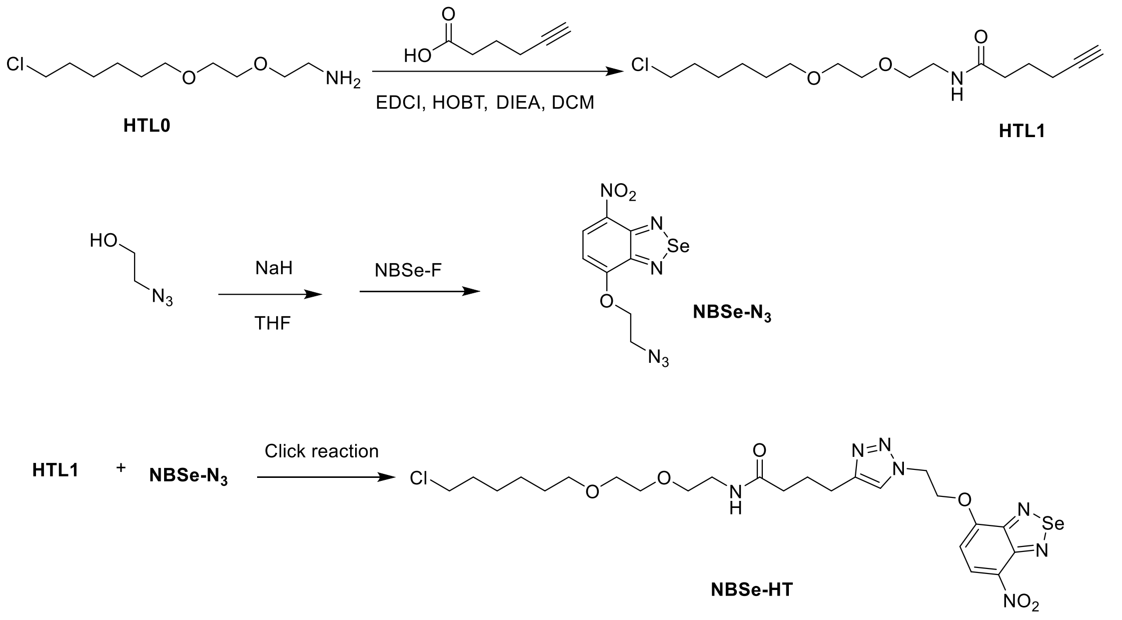

HTL0 was synthesized according to literature method.[13]

To a solution of 5-hexynoic acid (168 mg, 1.5 mmol), EDCI (337 mg, 1.8 mmol) and HOBT (270 mg, 2.0 mmol) in anhydrous DCM (20 mL) were added **HTL0** (223 mg, 1.0 mmol) and DIEA (516 mg, 4.0 mmol). The resulting solution was stirred at room temperature for 12 h, and then poured into 50 mL brine. The obtained mixture was extracted with DCM three times (3 x 50 mL). The organic layers were collected and dried over anhydrous Na_2SO_4. The organic solvent was removed to give a residue, which was subjected to silica gel column chromatography purification (DCM: MeOH = 30: 1, v/v) to afford 150 mg **HTL1** as a yellowish oil, yield: 47.3%. ^{1}H NMR (400 MHz, $CDCl_3$) δ 3.67 – 3.63 (m, 2H), 3.62 – 3.53 (m, 6H), 3.52 – 3.45 (m, 4H), 2.56 – 2.49 (m, 2H), 2.31 – 2.27 (m, 2H), 2.01 (dd, J = 3.4, 1.6 Hz, 1H), 1.88 (dd, J = 7.1, 4.5 Hz, 2H), 1.85 – 1.76 (m, 2H), 1.47 – 1.41 (m, 6H). ^{13}C NMR (100 MHz, $CDCl_3$) δ 172.61, 83.55, 71.22, 69.96, 69.08, 53.65, 45.17, 39.12, 34.96, 32.51, 29.33, 26.67, 25.41, 24.21, 23.48, 17.75. HRMS (ESI) m/z for $C_{16}H_{29}ClNO_3$ $[M+H]^+$, calcd.: 318.1830, found:318.1857.

At room temperature, 32 mg NaH (60% in oil, 0.8 mmol) was added to the solution of 2-azidoethanol (87 mg, 1 mmol) in anhydrous THF (50 mL). After stirring for 10 min, **NBSe-F** (148 mg, 0.6 mmol) was added to the above solution. The mixture was stirred at room temperature for 4 h, and then poured into 50 mL ice water. The reaction mixture was extracted with DCM three times (3 x 50 mL), and the organic layers were collected and dried over anhydrous Na_2SO_4. The solvent was removed by distillation and the residue was purified with silica gel column chromatograph (PE: DCM = 3: 1, v/v) to give 105 mg **NBSe-N$_3$** as a yellow solid, yield: 55.6%. ^{1}H NMR (400 MHz, DMSO-d_6) δ 8.65 (d, J = 8.5 Hz, 1H), 7.03 (d, J = 8.6 Hz, 1H), 4.57 – 4.47 (m, 2H), 3.91 – 3.82 (m, 2H). ^{13}C NMR (100 MHz, DMSO-d_6) δ 156.31 (s), 153.33 (s), 152.01 (s), 135.05 (s), 131.92 (s),

104.25 (s), 68.95 (s), 49.84 (s). HRMS (ESI) m/z for $C_8H_7N_6O_3Se$ $[M+H]^+$, calcd.: 314.9639, found:314.9769.

NBSe-N$_3$ (13 mg, 0.04 mmol), **HTL1** (9 mg, 0.03 mmol), THPTA (65 mg, 0.15 mmol), $CuSO_4$ (24 mg, 0.15 mmol) and ascorbic acid (26 mg, 0.15 mmol) were dissolved in 6 mL DMSO. The reaction solution was stirred at room temperature for 6 h, and then poured into 50 mL brine. The obtained mixture was extracted with DCM three times (3 x 50 mL), and the organic layers were collected and dried over anhydrous Na_2SO_4. The solvent was removed by distillation and the residue was purified by silica gel column chromatograph (DCM: MeOH = 10: 1, v/v) to give 11 mg **NBSe-HT** as a yellow solid, yield: 58.0%. ^{1}H NMR (400 MHz, $CDCl_3$) δ 8.62 (d, J = 8.4 Hz, 1H), 7.81 (s, 1H), 6.74 (d, J = 8.5 Hz, 1H), 6.26 (s, 1H), 4.98 (t, J = 4.9 Hz, 2H), 4.73 (t, J = 4.9 Hz, 2H), 3.67 (s, 6H), 3.61 – 3.56 (m, 4H), 3.48 (dd, J = 8.8, 4.3 Hz, 4H), 2.78 (t, J = 7.4 Hz, 2H), 2.25 (t, J = 7.5 Hz, 2H), 2.03 – 1.98 (m, 2H), 1.81 – 1.77 (m, 2H), 1.64 – 1.59 (m, 2H), 1.46 (dd, J = 15.0, 7.6 Hz, 2H). ^{13}C NMR (100 MHz, $CDCl_3$) δ 172.56, 170.47, 164.37, 155.37, 150.02, 131.14, 103.29, 71.28, 70.57, 70.25, 70.03, 69.83, 68.23, 45.10, 39.16, 35.61, 32.51, 29.72, 29.41, 26.68, 25.45, 24.77. HRMS (ESI) m/z for $C_{24}H_{34}ClN_7O_6Se$ $[M+H]^+$, calcd.: 632.1497, found:632.1525.

12. NMR and HRMS spectra

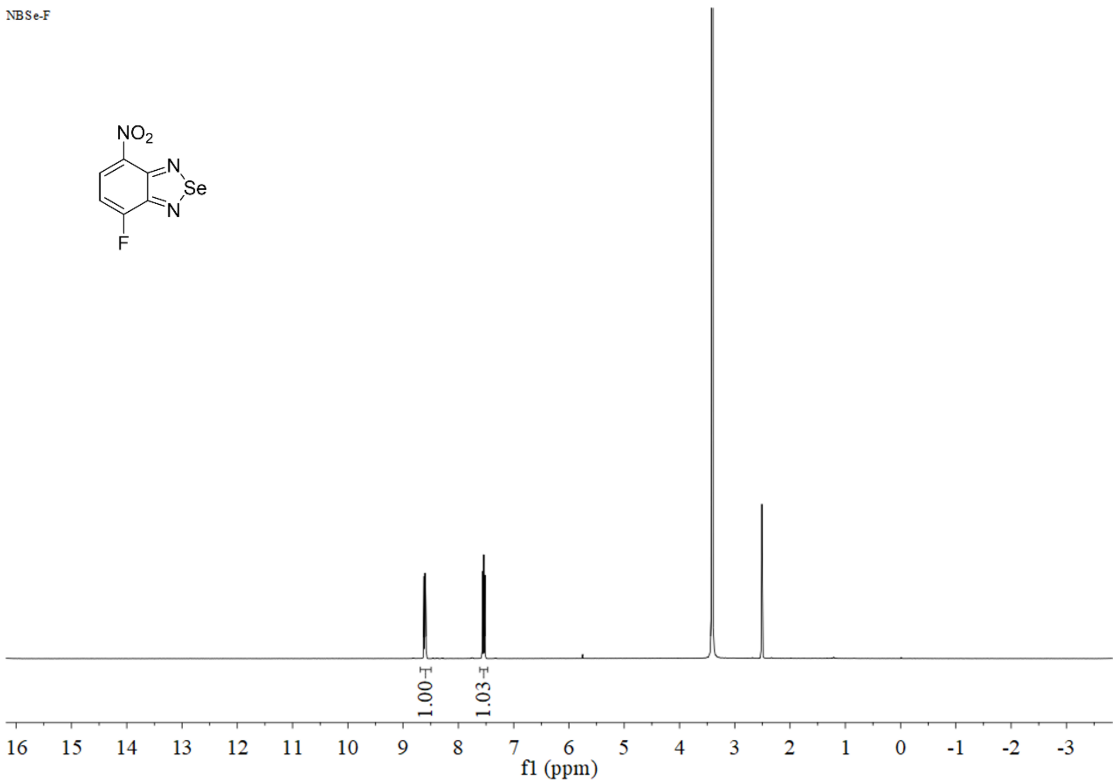

^{1}H NMR spectrum of compound **NBSe-F** in DMSO-d_6.

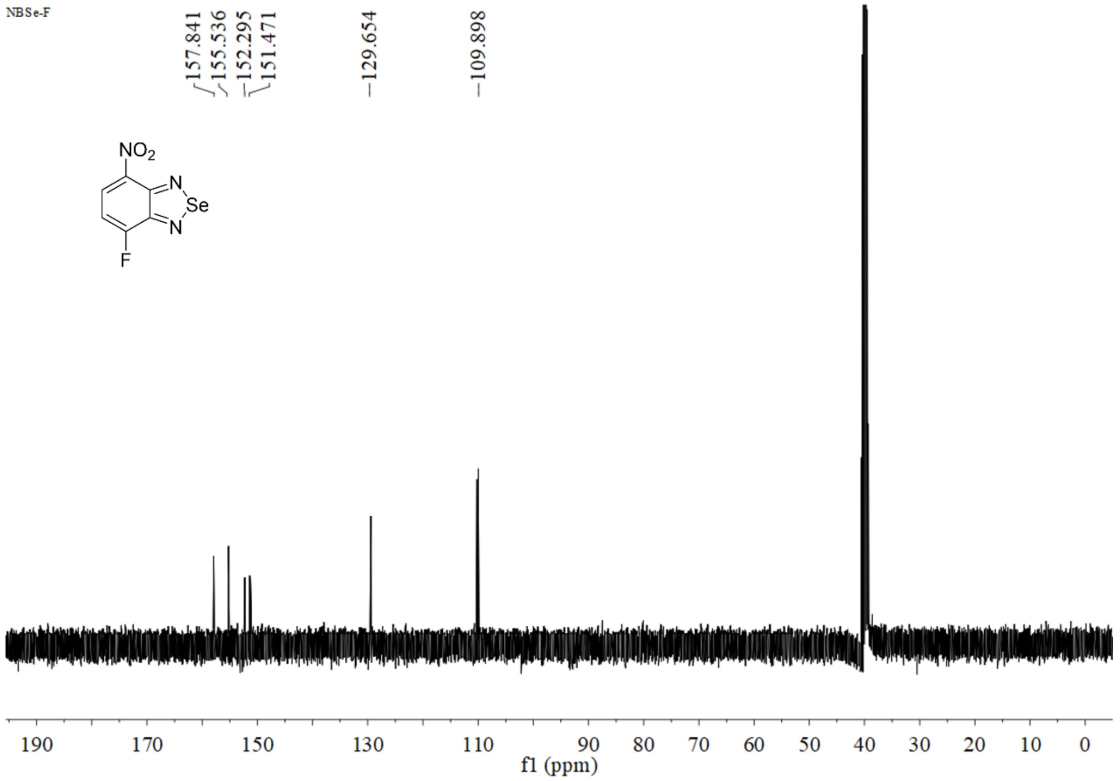

^{13}C NMR spectrum of compound **NBSe-F** in DMSO-d_6.

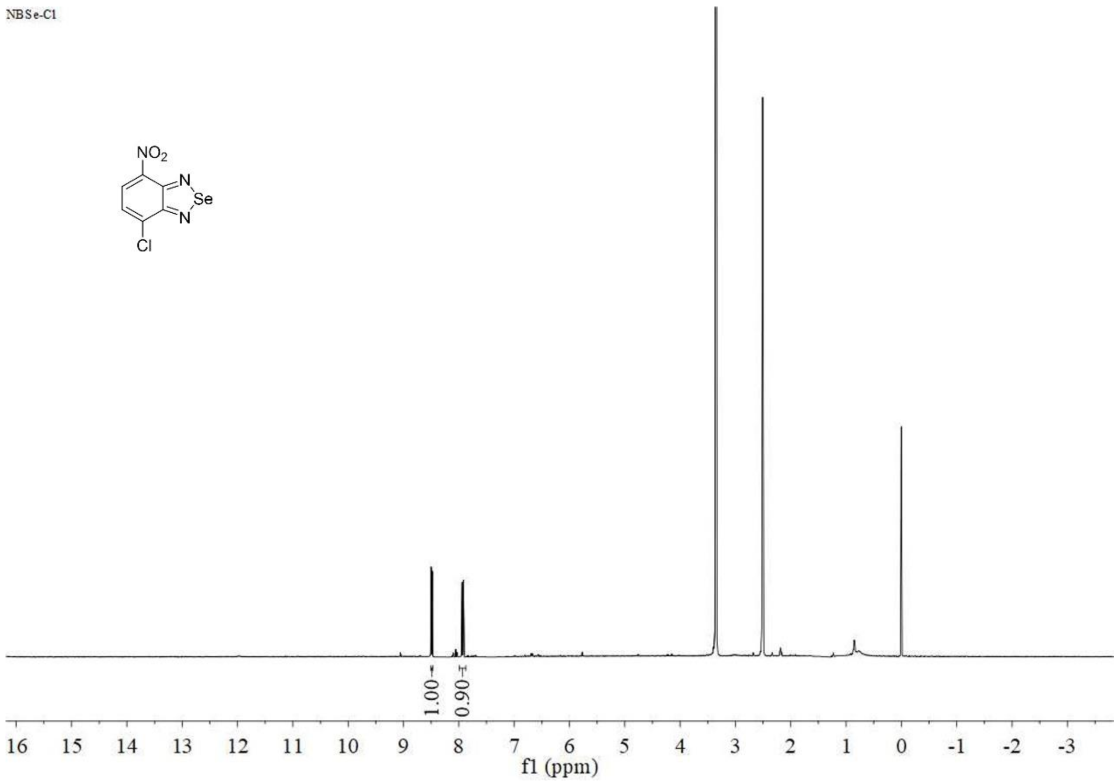

^{1}H NMR spectrum of compound **NBSe-Cl** in DMSO-d_6.

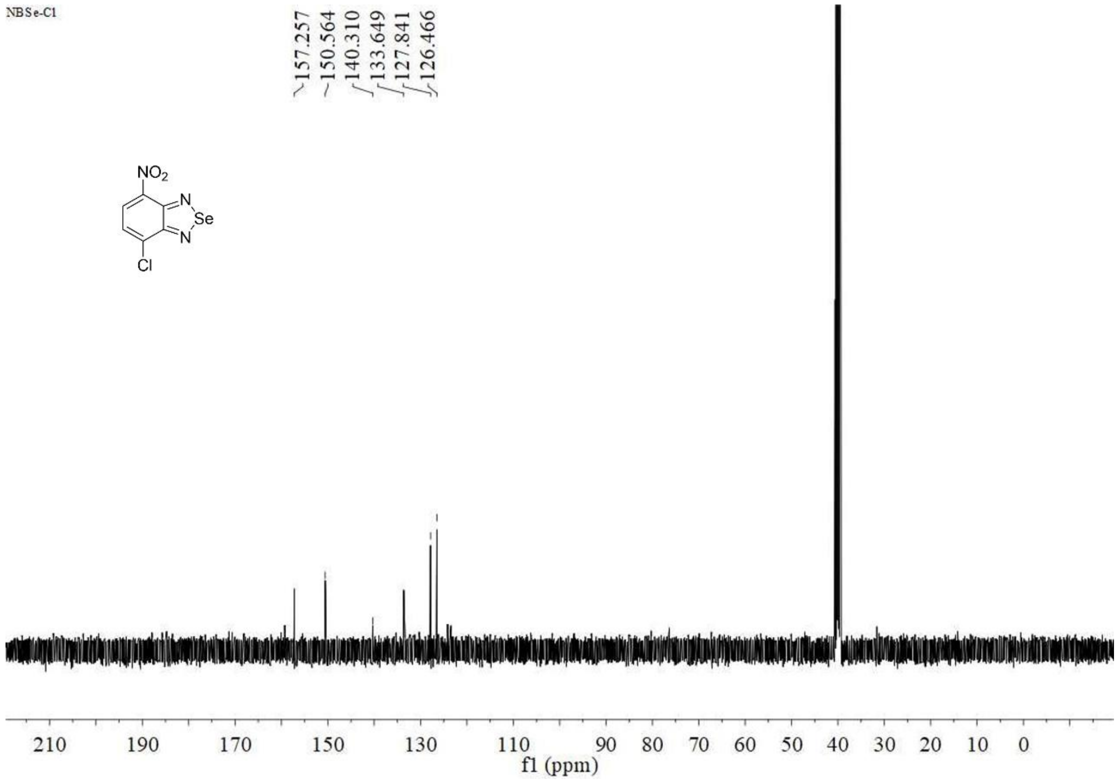

^{13}C NMR spectrum of compound **NBSe-Cl** in DMSO-d_6.

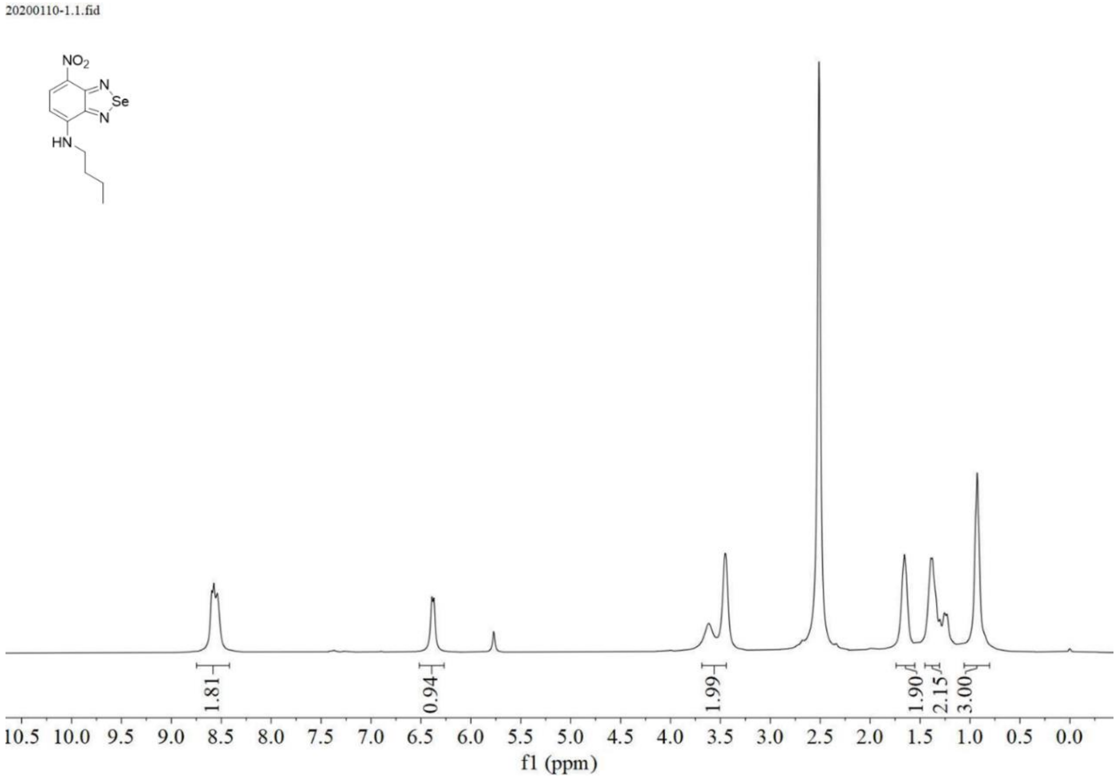

^{1}H NMR spectrum of compound **NBSe-N-nBu** in DMSO-d_6.

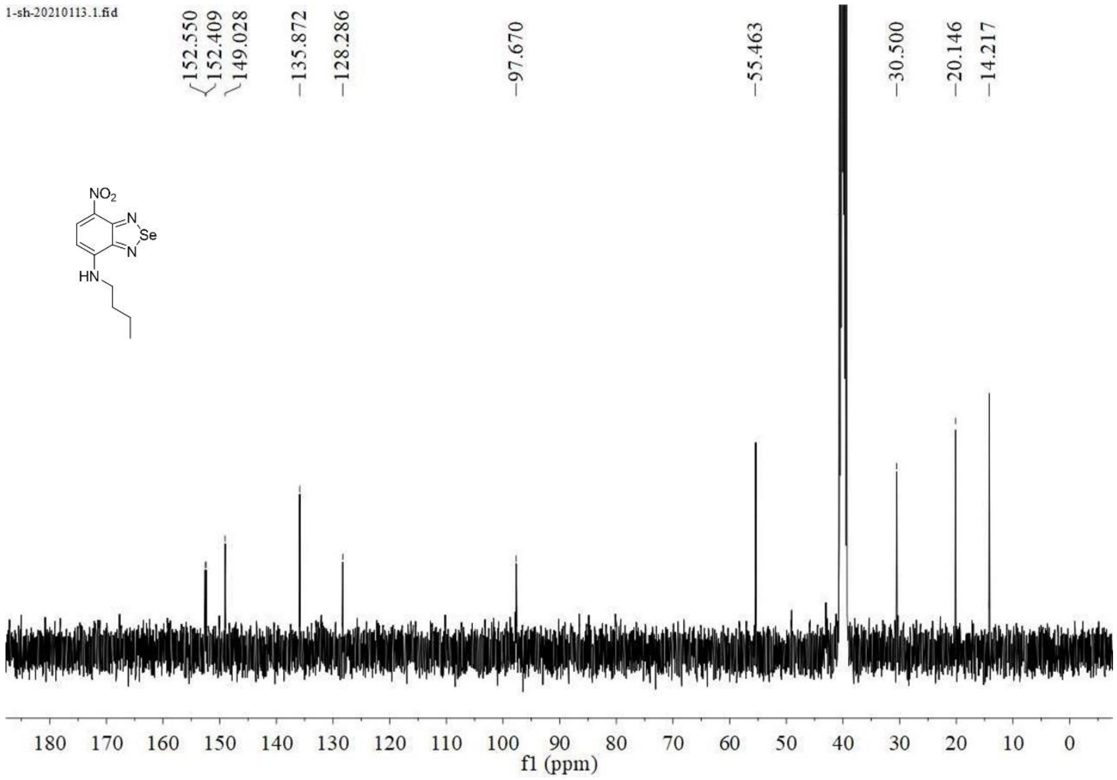

^{13}C NMR spectrum of compound **NBSe-N-nBu** in DMSO-d_6.

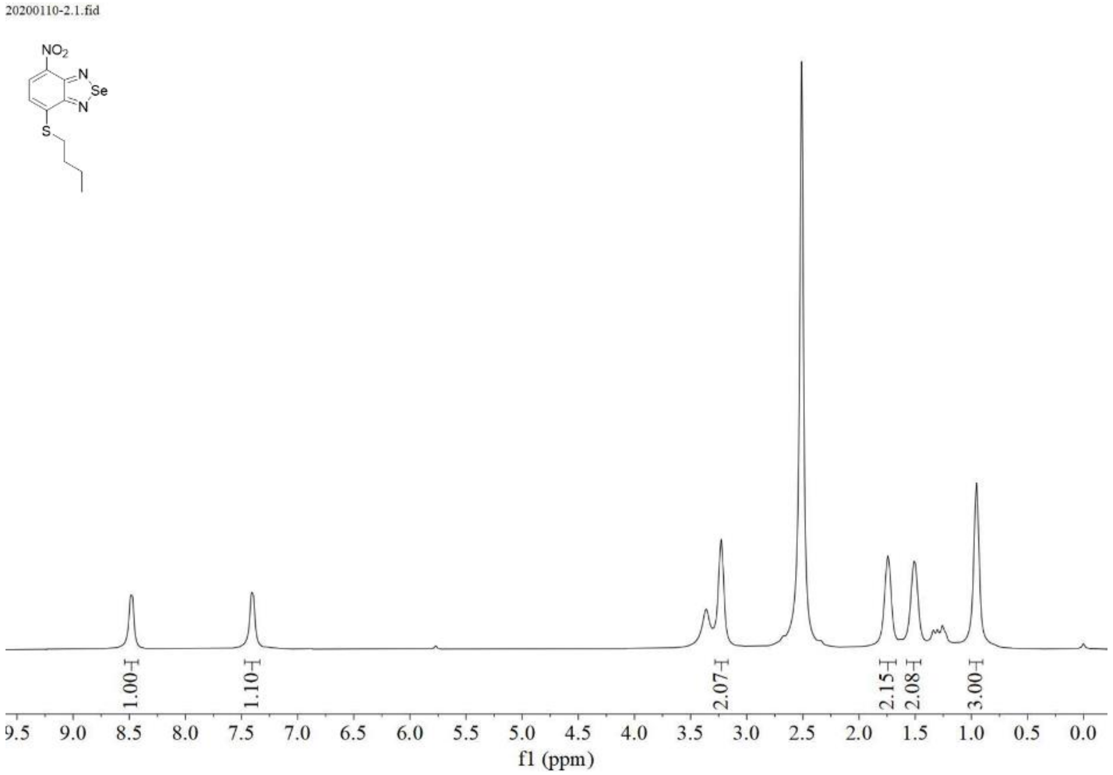

^{1}H NMR spectrum of compound **NBSe-S-nBu** in DMSO-*d6*.

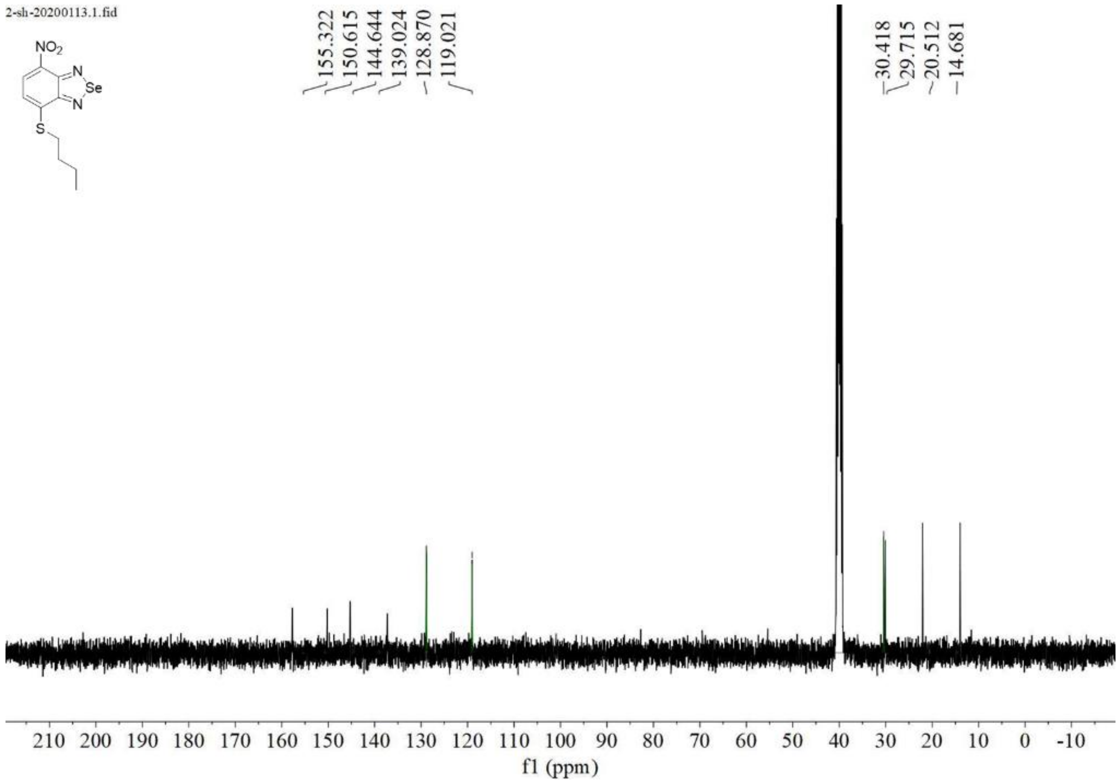

^{13}C NMR spectrum of compound **NBSe-S-nBu** in DMSO-*d6*.

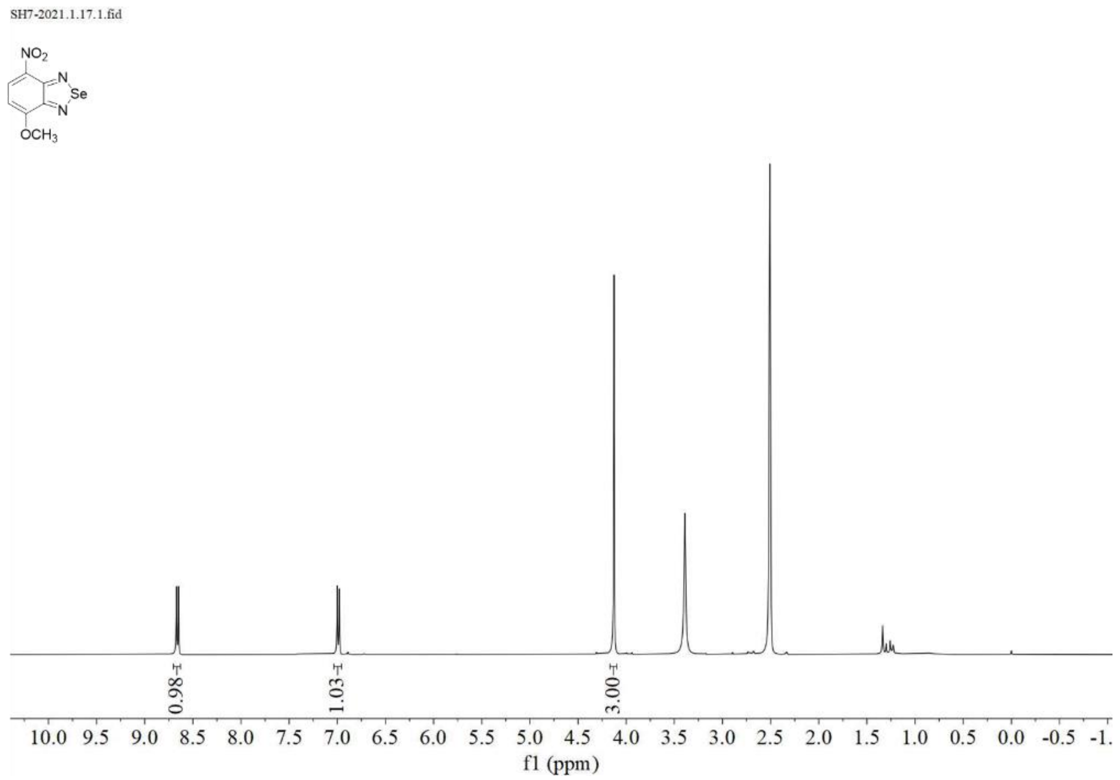

^1H NMR spectrum of compound **NBSe-OMe** in DMSO-d_6.

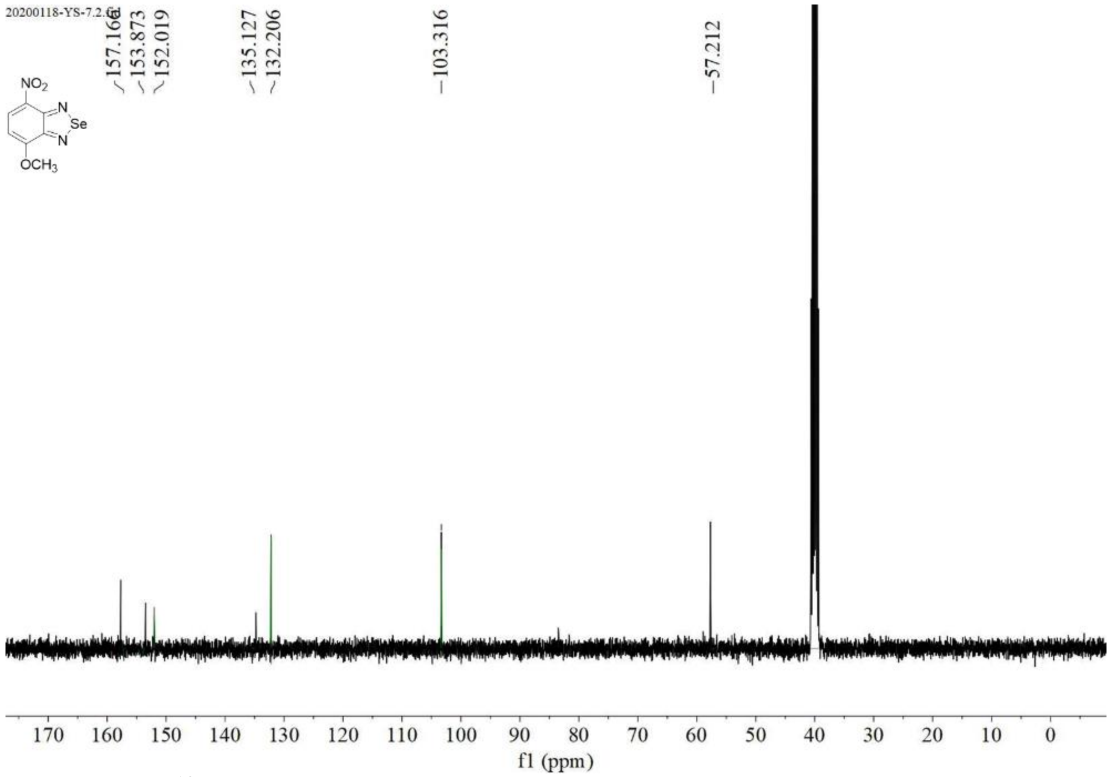

^{13}C NMR spectrum of compound **NBSe-OMe** in DMSO-d_6.

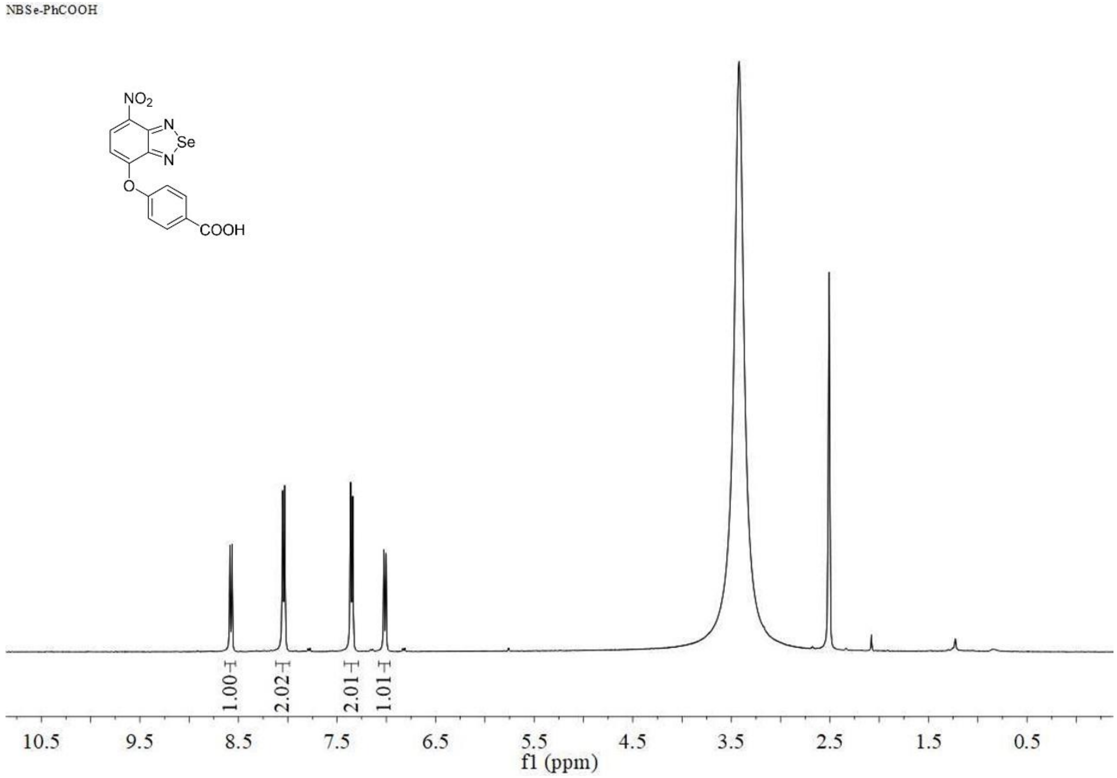

^{1}H NMR spectrum of compound **NBSe-OPh1** in DMSO-*d₆*.

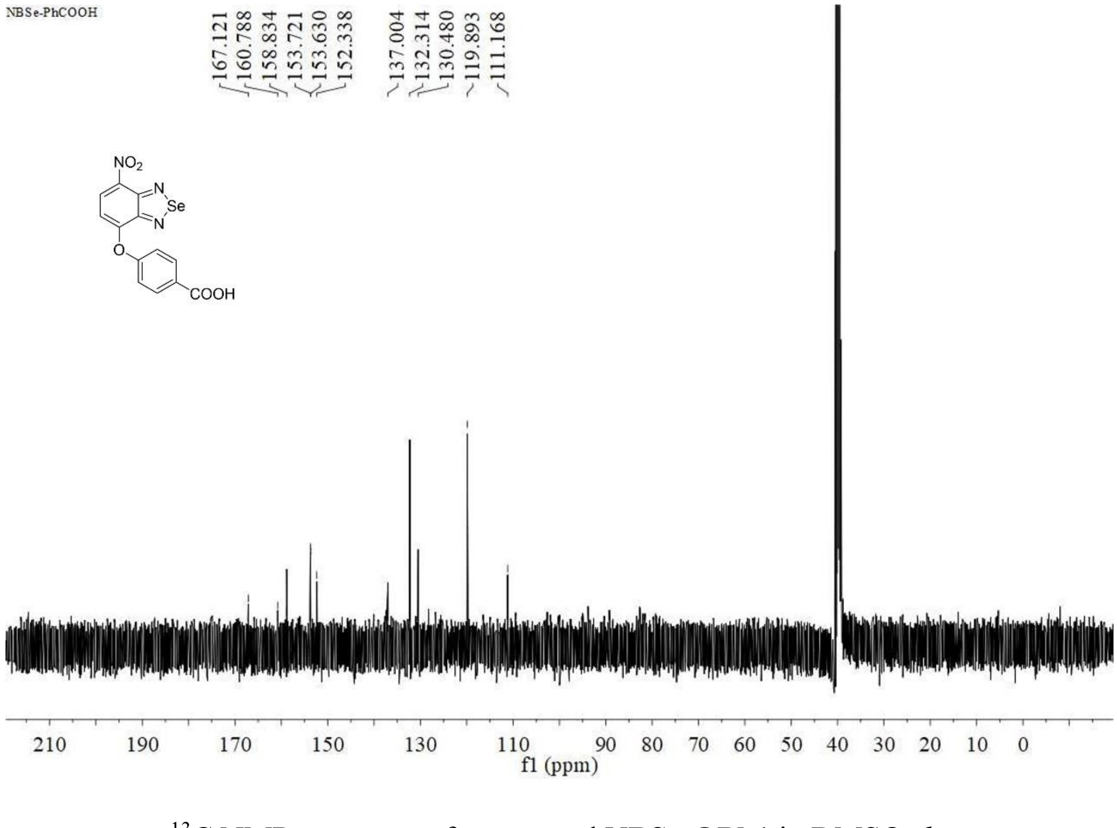

^{13}C NMR spectrum of compound **NBSe-OPh1** in DMSO-*d₆*.

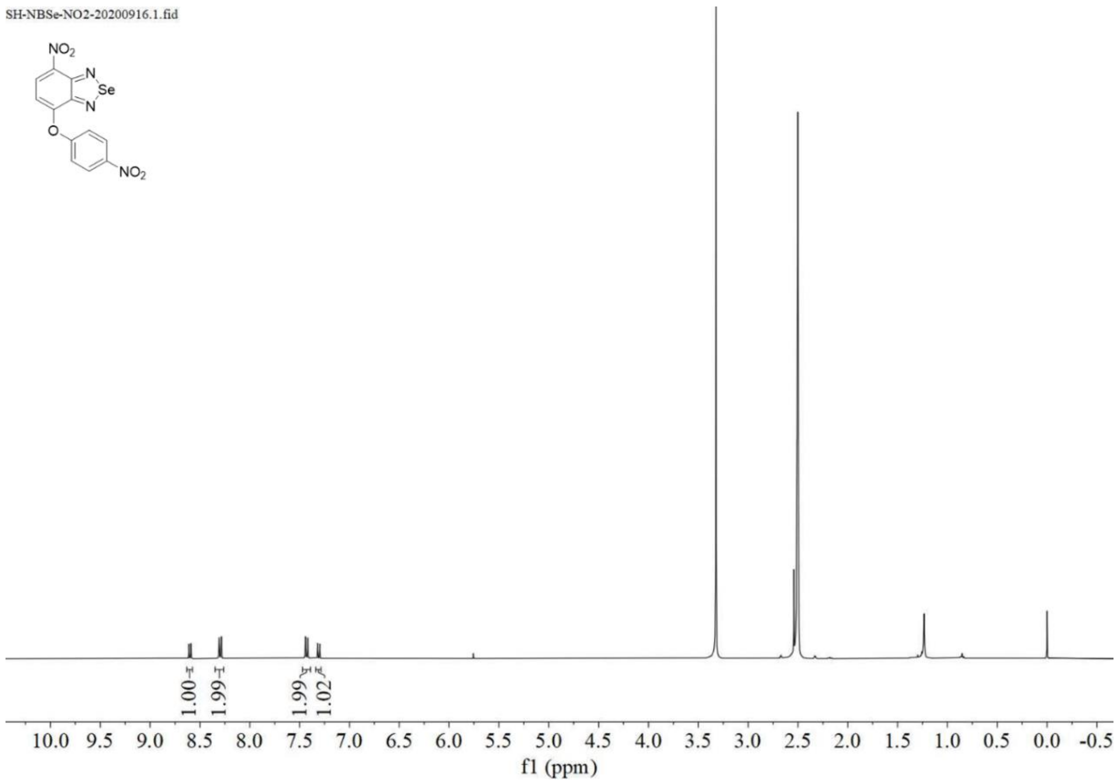

^{1}H NMR spectrum of compound **NBSe-OPh2** in DMSO-*d6*.

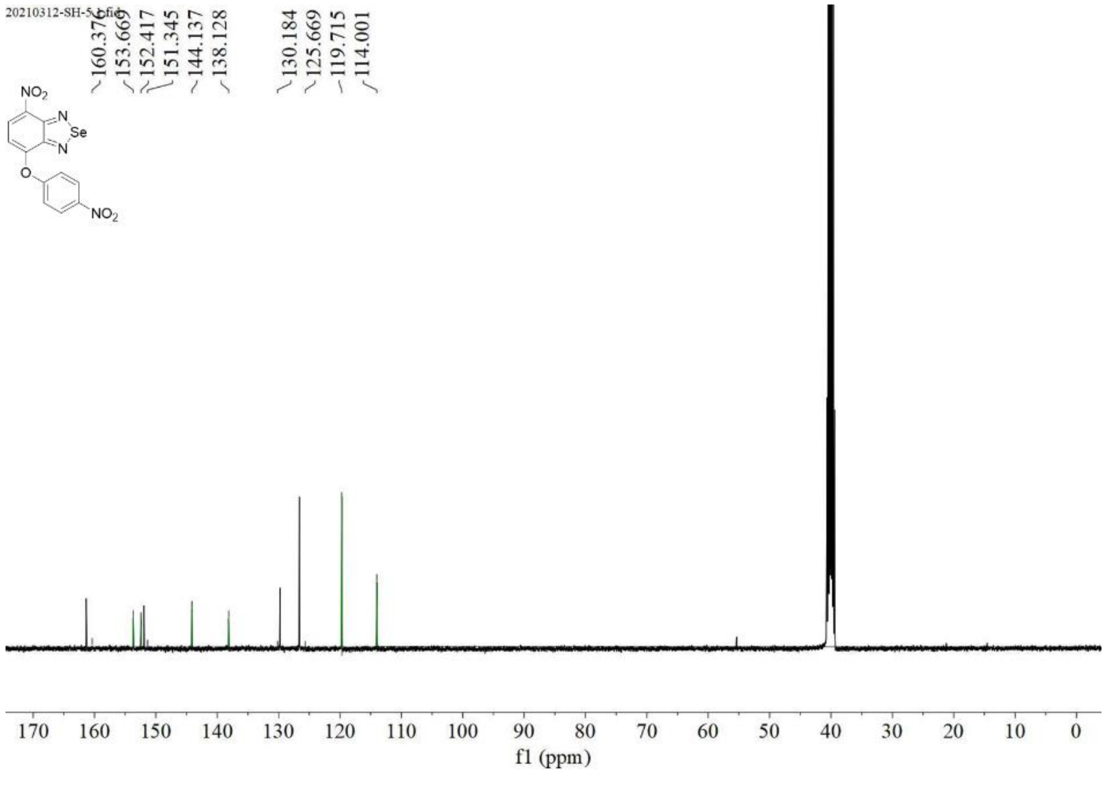

^{13}C NMR spectrum of compound **NBSe-OPh2** in DMSO-*d6*.

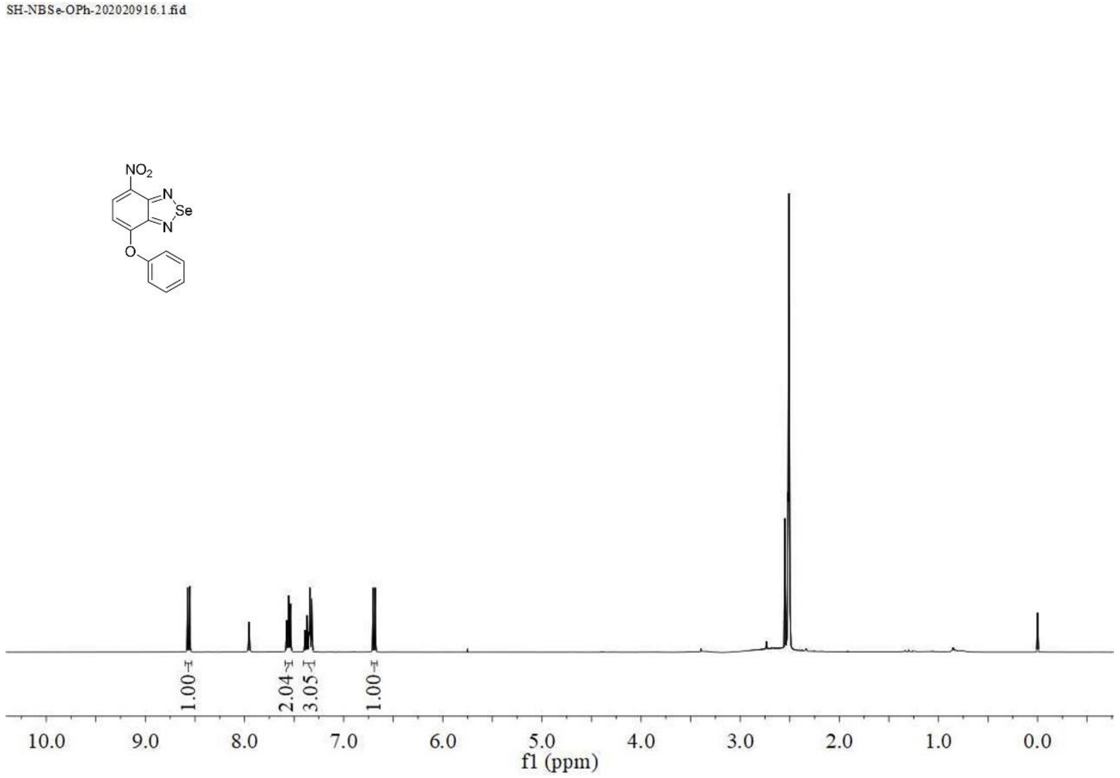

^{1}H NMR spectrum of compound **NBSe-OPh3** in DMSO-*d6*.

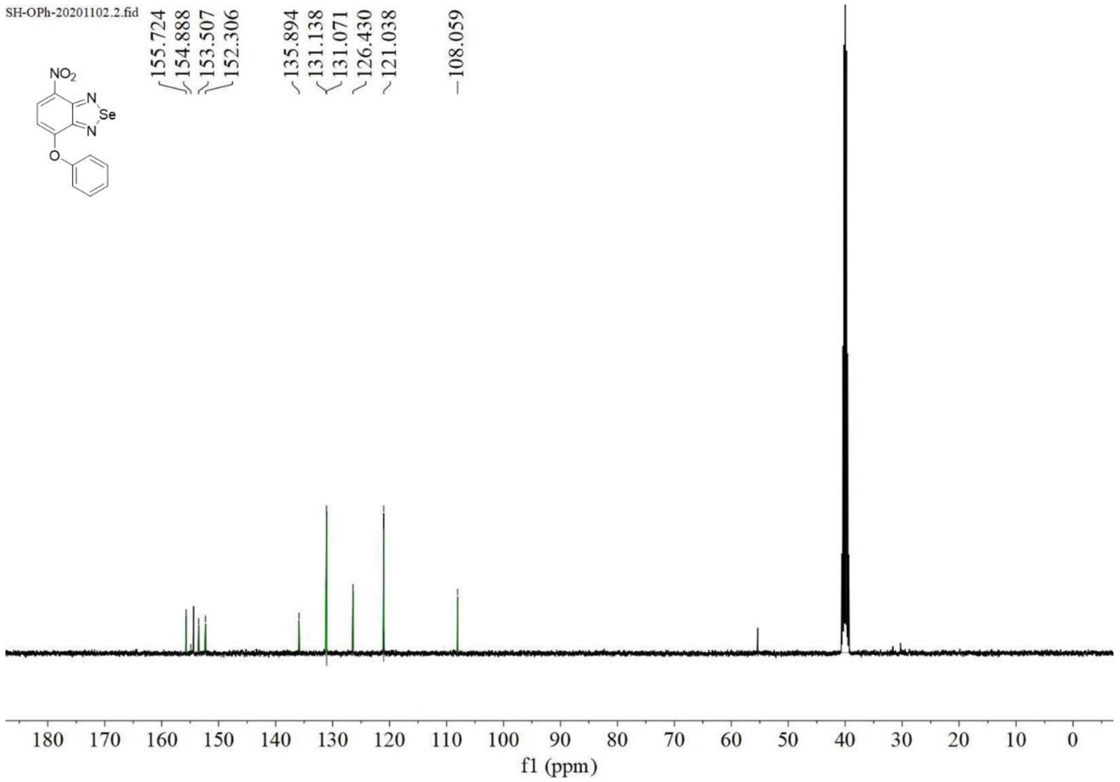

^{13}C NMR spectrum of compound **NBSe-OPh3** in DMSO-*d6*.

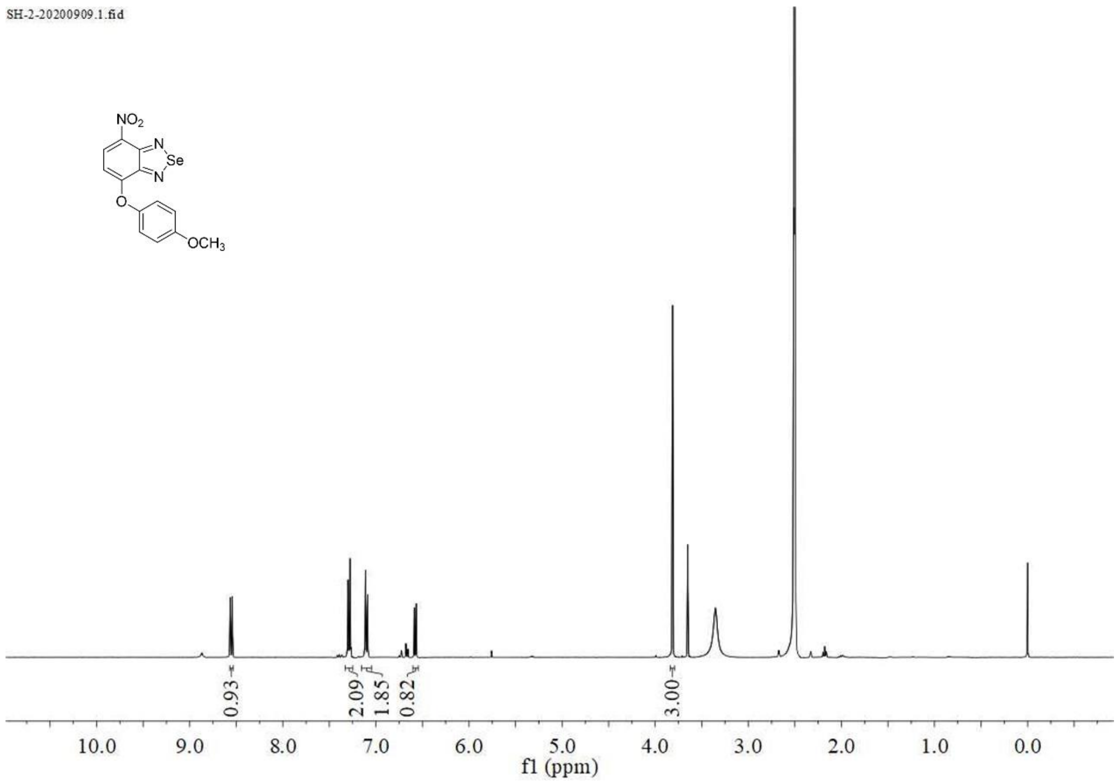

^{1}H NMR spectrum of compound **NBSe-OPh4** in DMSO-*d6*.

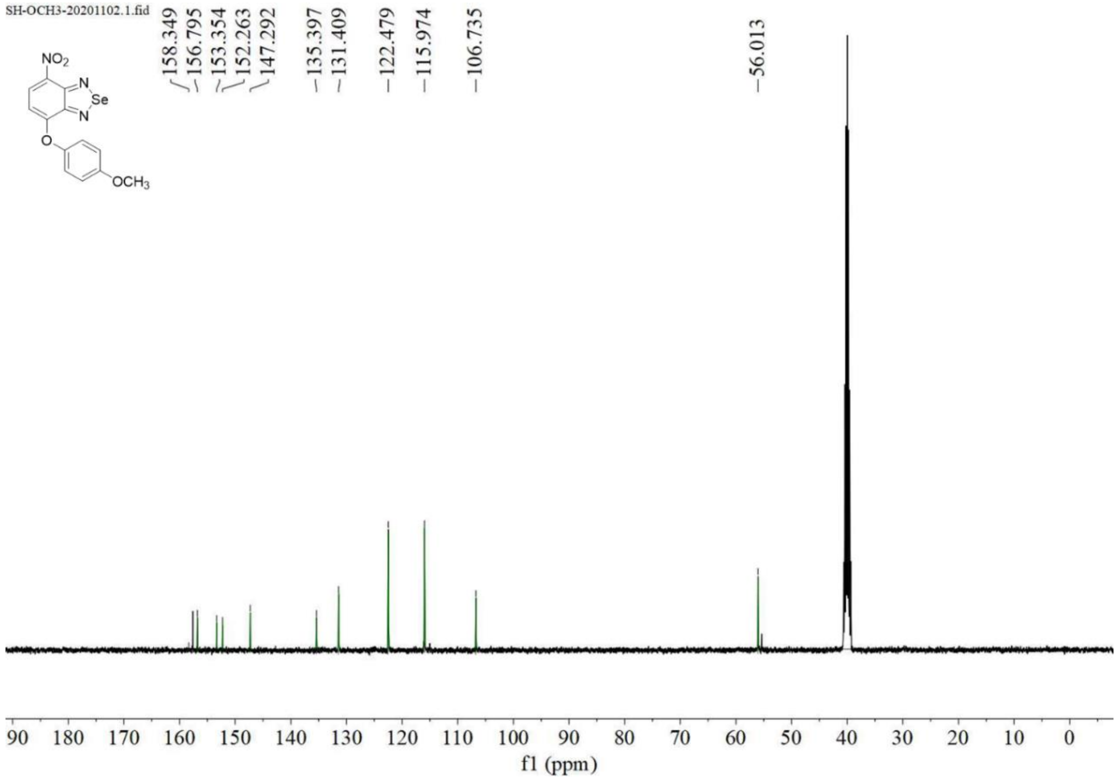

^{13}C NMR spectrum of compound **NBSe-OPh4** in DMSO-*d6*.

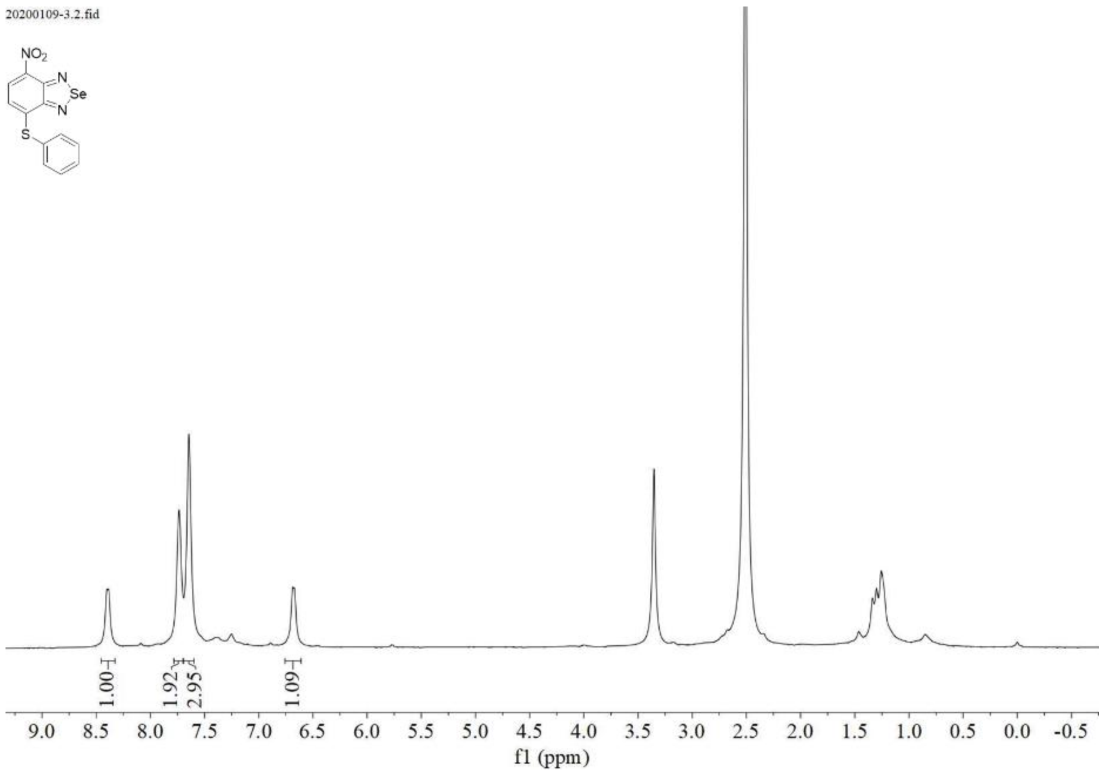

^{1}H NMR spectrum of compound **NBSe-SPh** in DMSO-d_6.

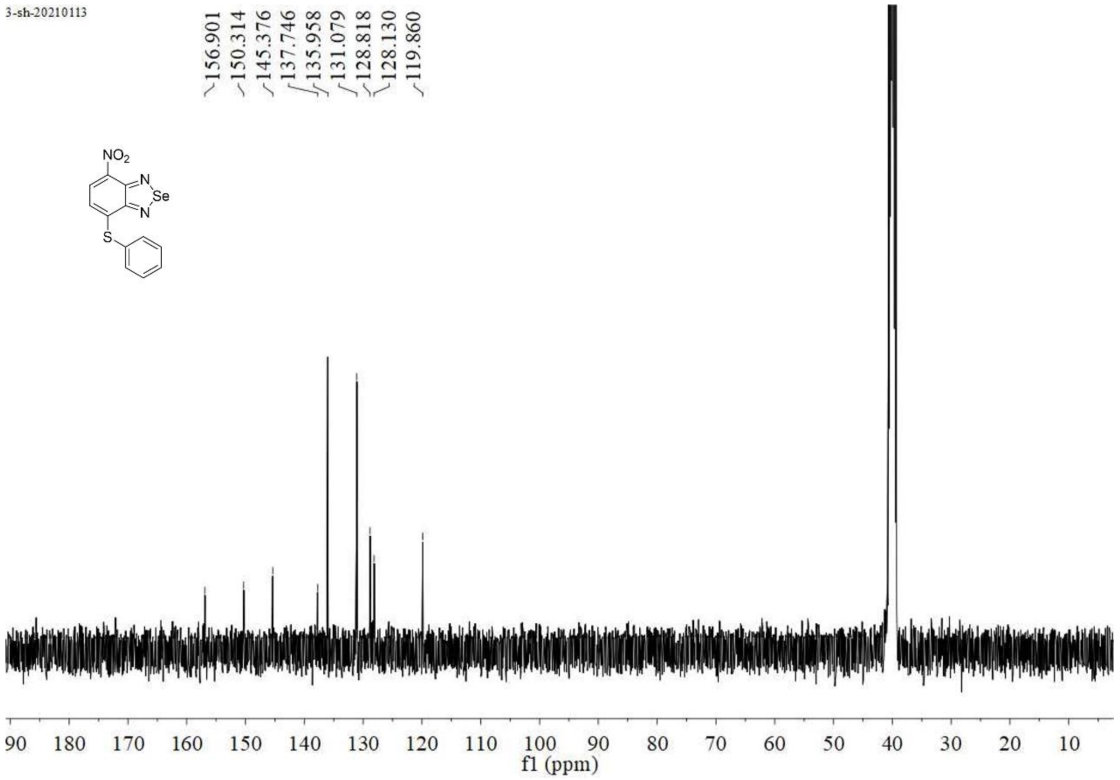

^{13}C NMR spectrum of compound **NBSe-SPh** in DMSO-d_6.

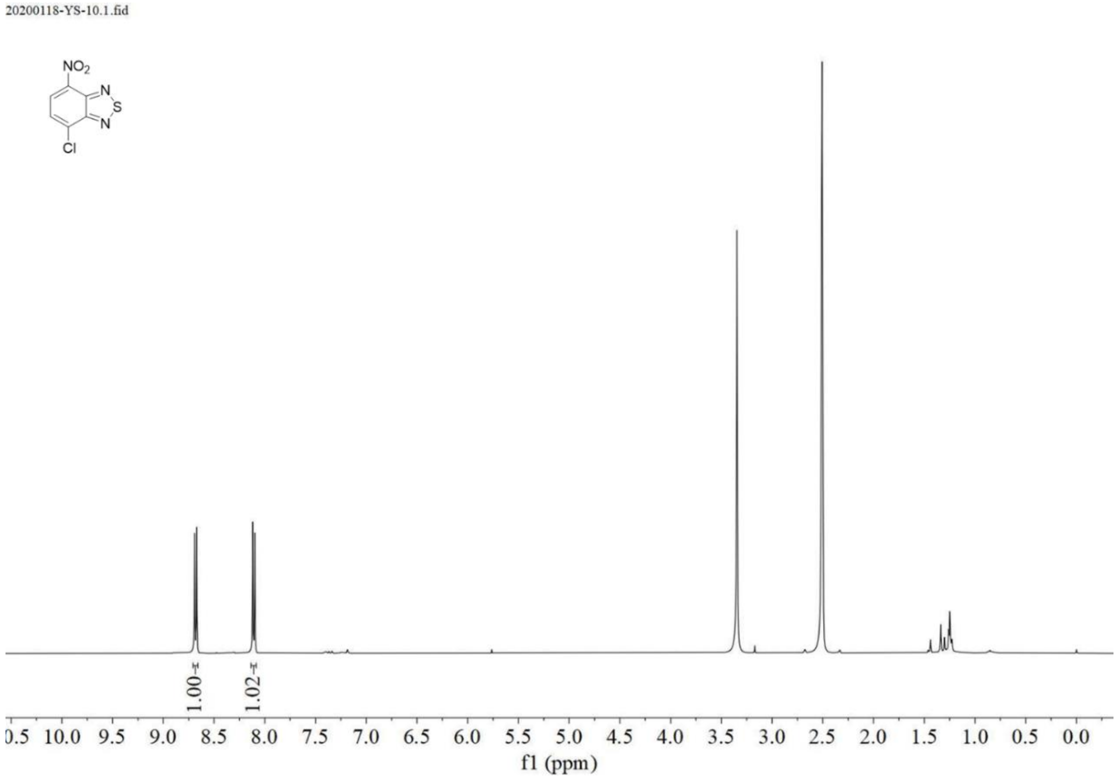

^{1}H NMR spectrum of compound **NBS-Cl** in DMSO-d_6.

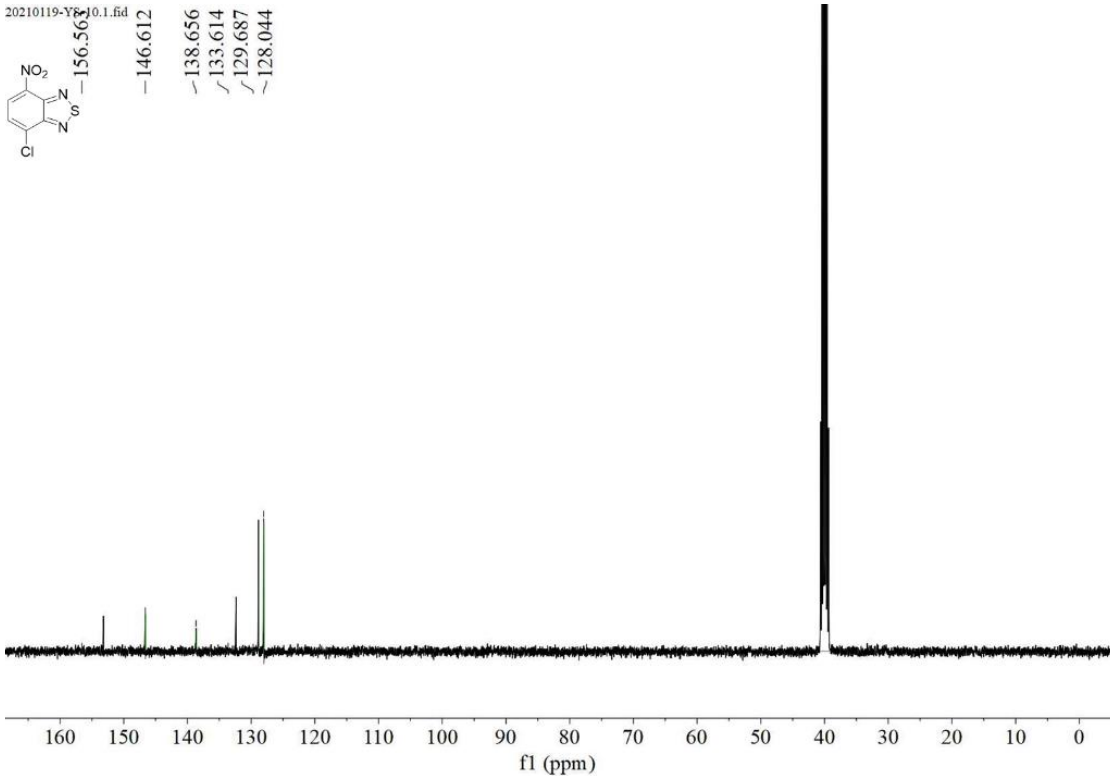

^{13}C NMR spectrum of compound **NBS-Cl** in DMSO-d_6.

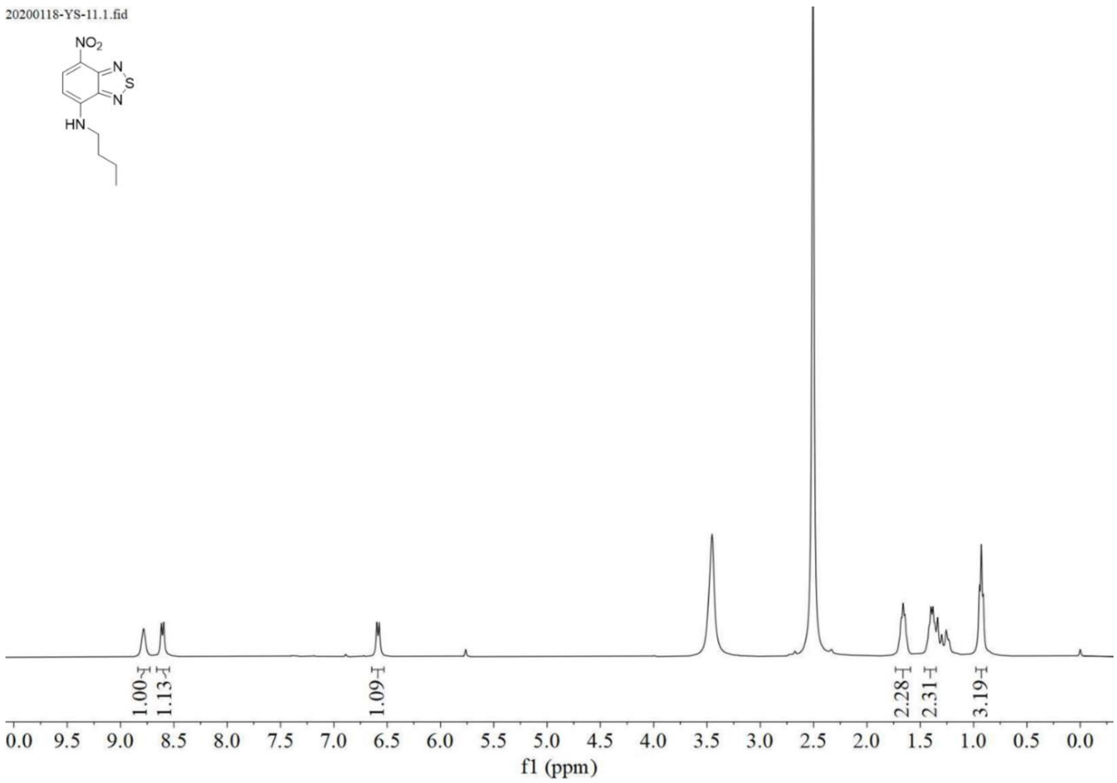

^{1}H NMR spectrum of compound **NBS-N-nBu** in DMSO-d_6.

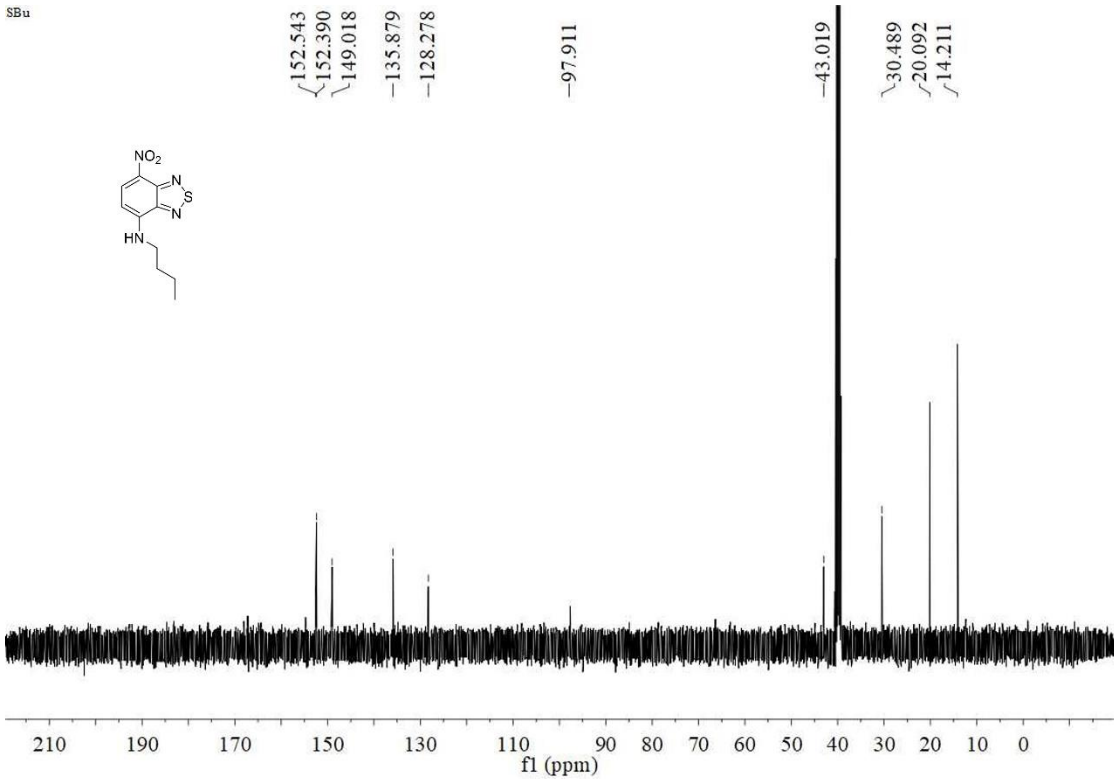

^{13}C NMR spectrum of compound **NBS-N-nBu** in DMSO-d_6.

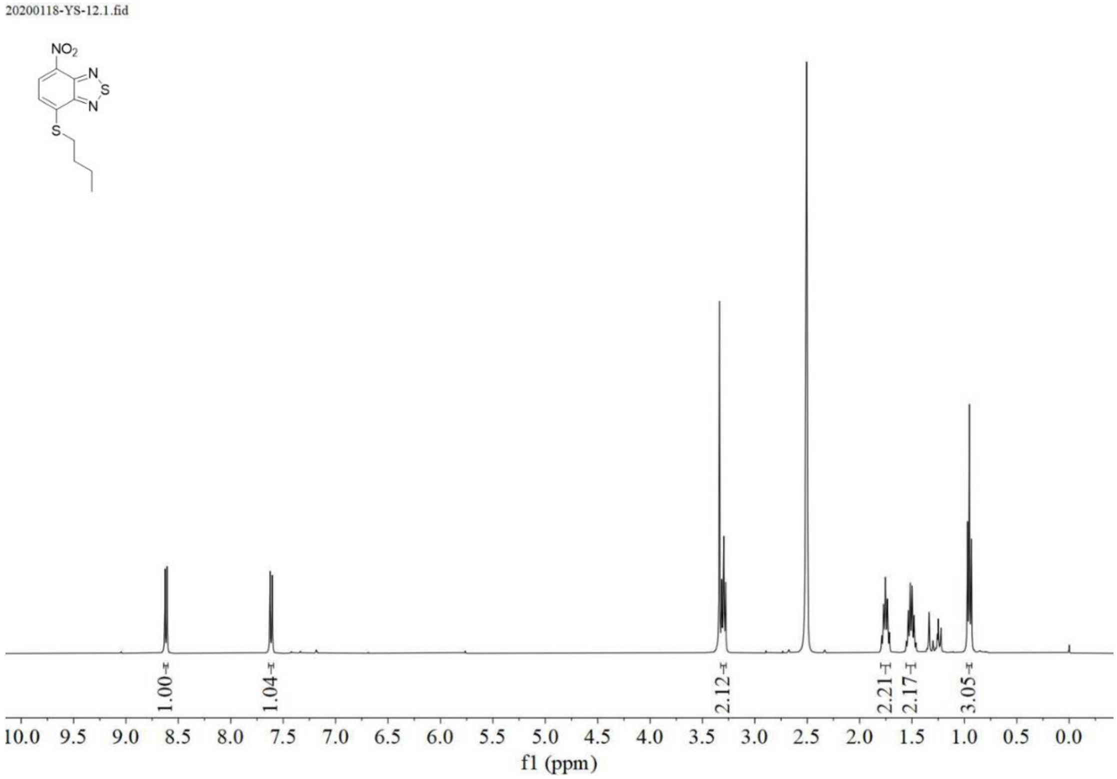

^{1}H NMR spectrum of compound **NBS-S-nBu** in DMSO-d_6.

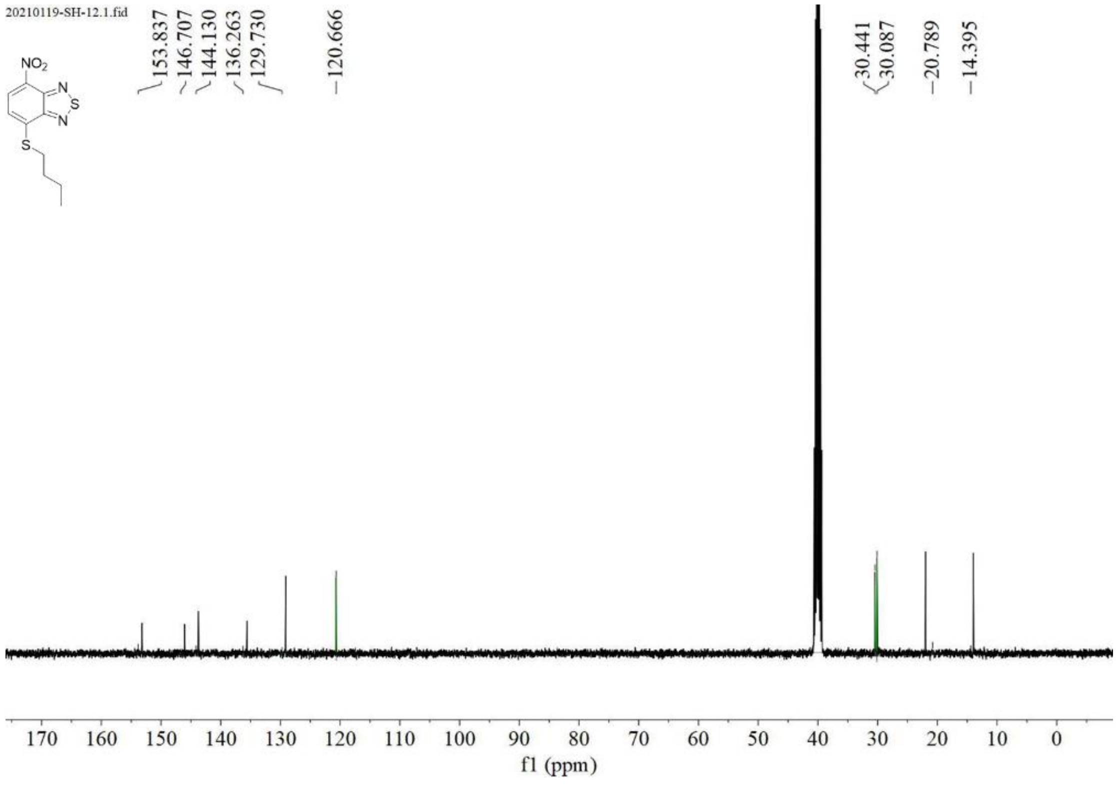

^{13}C NMR spectrum of compound **NBS-S-nBu** in DMSO-d_6.

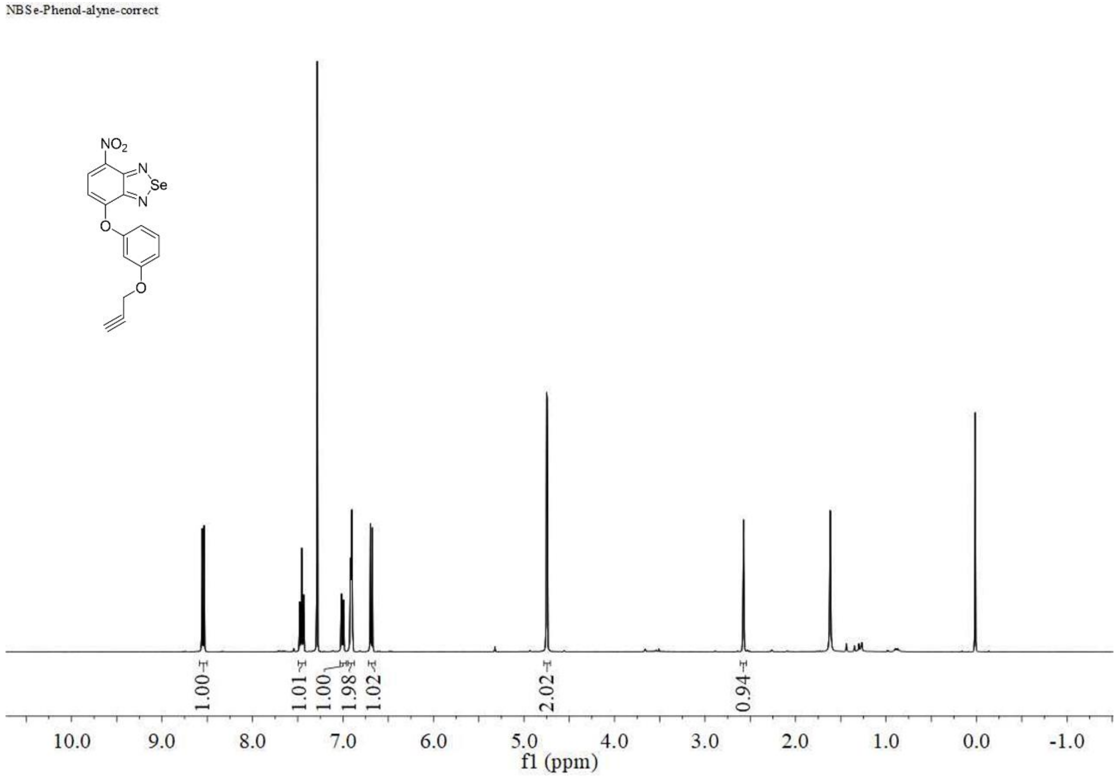

^{1}H NMR spectrum of compound **B2** in $CDCl_3$.

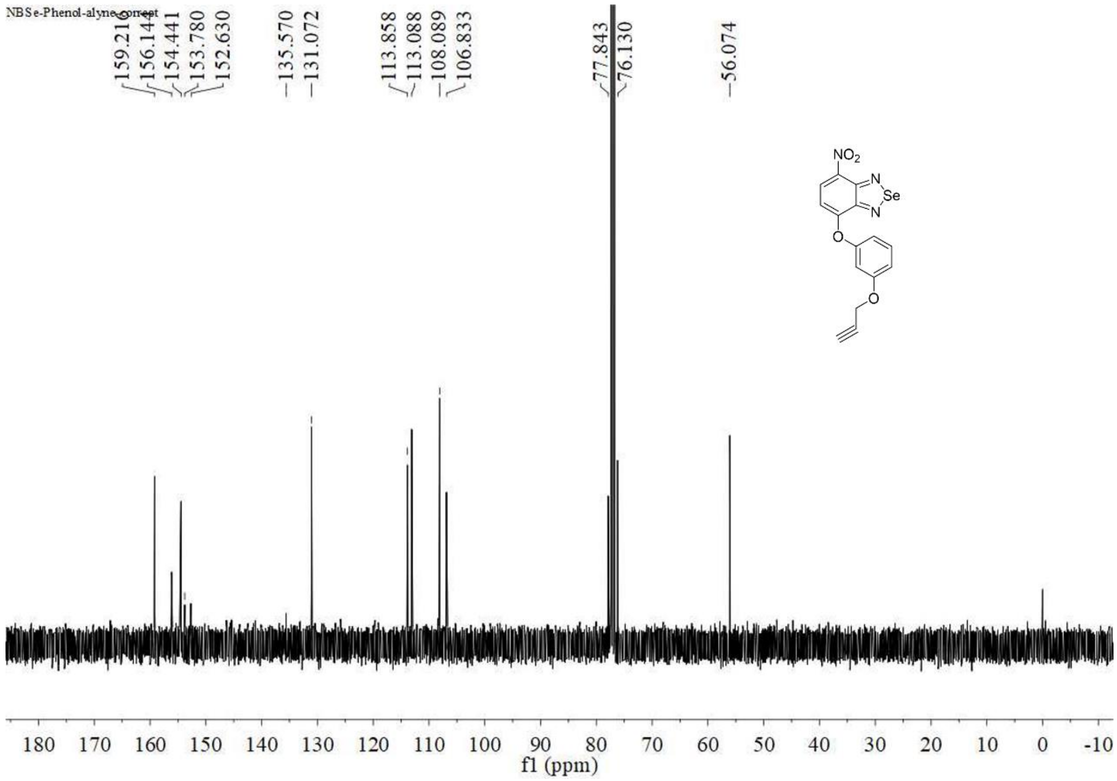

^{13}C NMR spectrum of compound **B2** in $CDCl_3$.

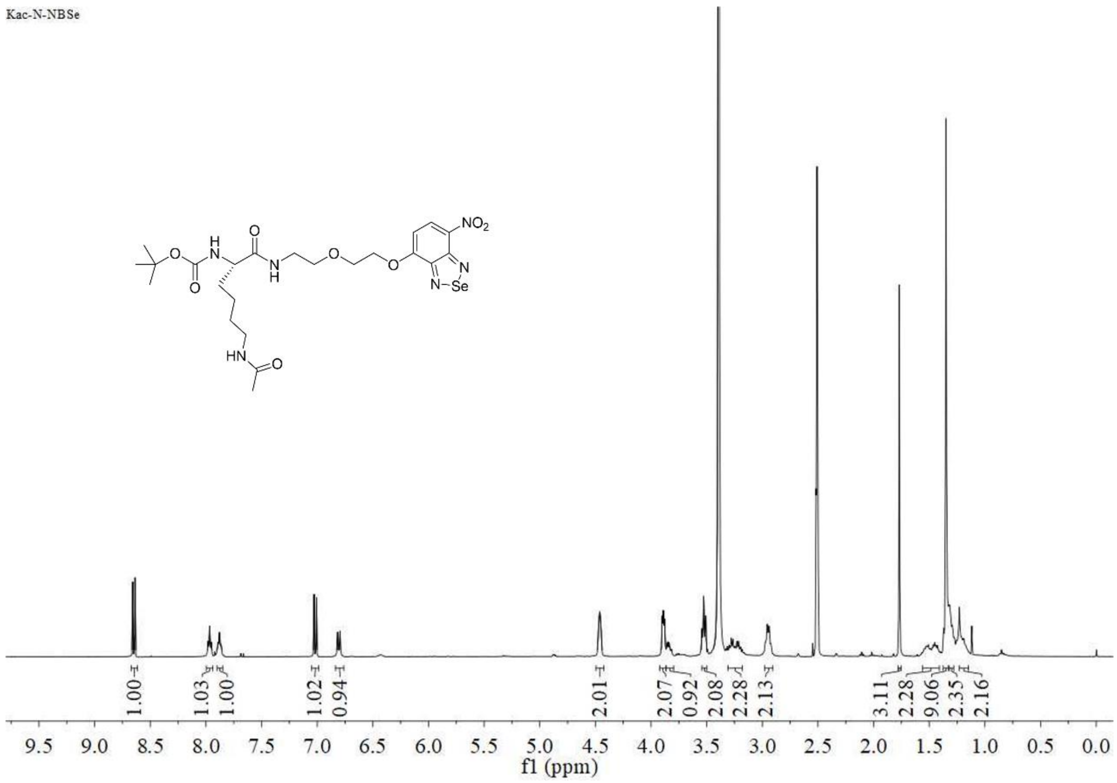

^1H NMR spectrum of compound **NBSe-HDAC** in DMSO-d_6.

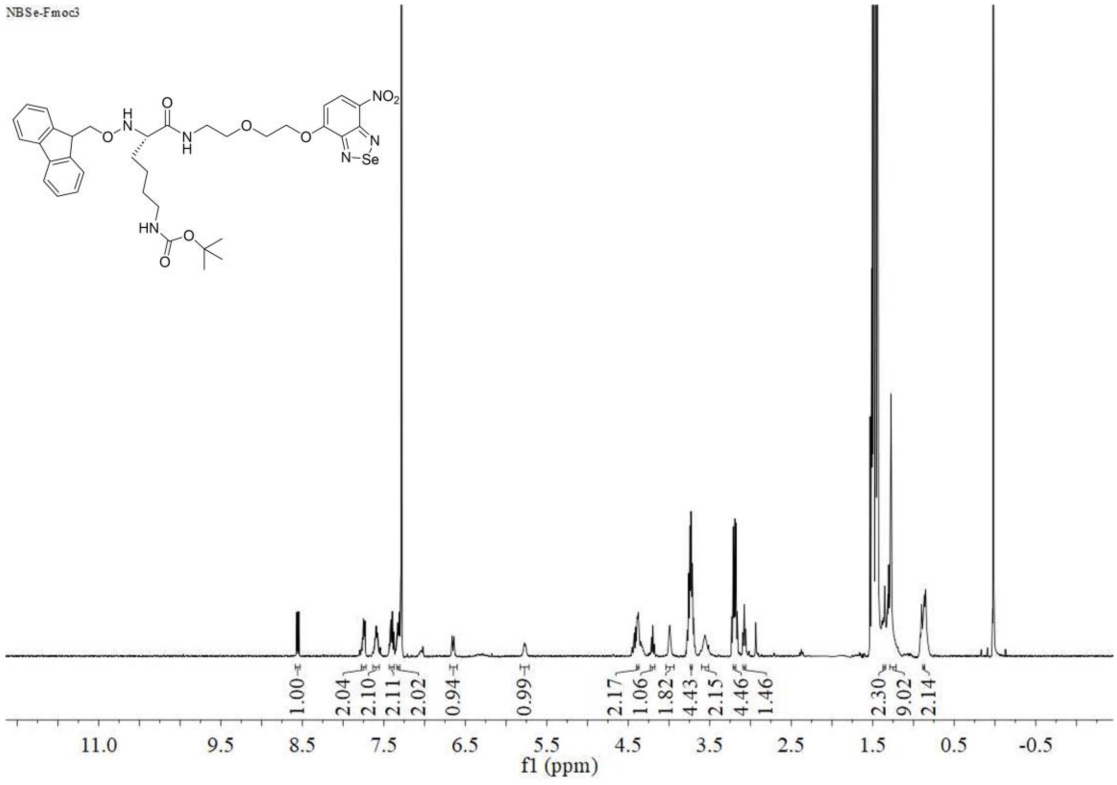

^1H NMR spectrum of compound **NBSe-MD** in $CDCl_3$.

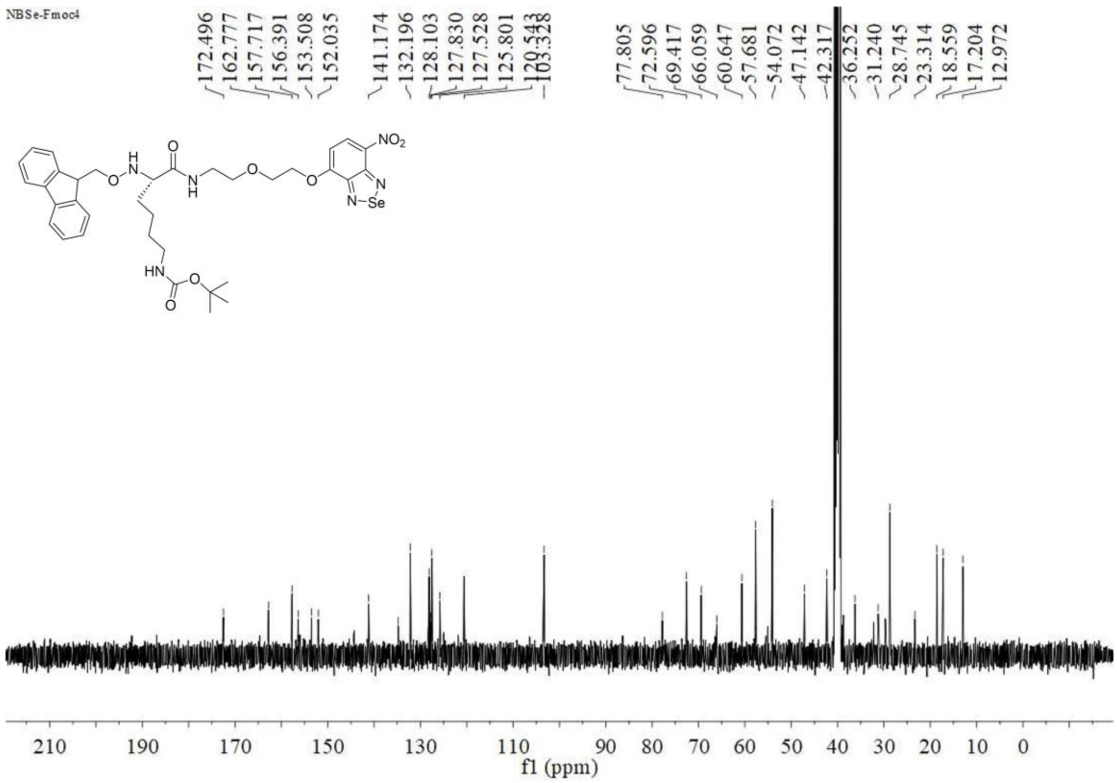

^{13}C NMR spectrum of compound **NBSe-MD** in DMSO-d_6.

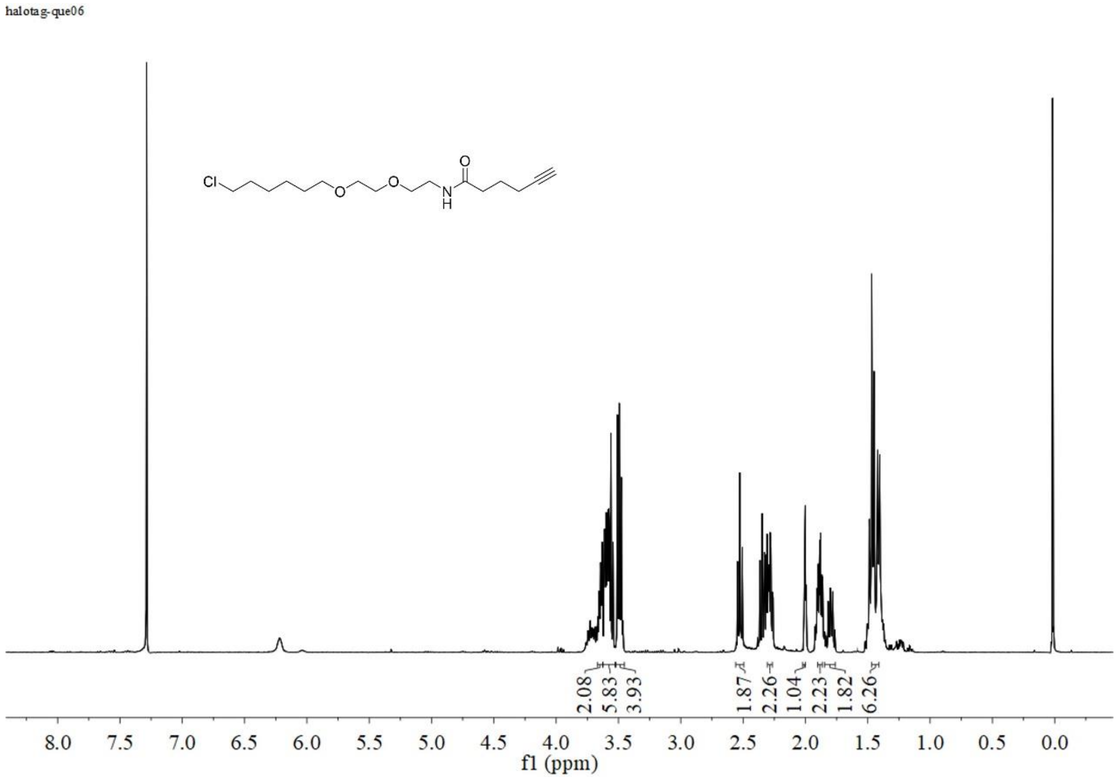

^{1}H NMR spectrum of compound **HTL1** in $CDCl_3$.

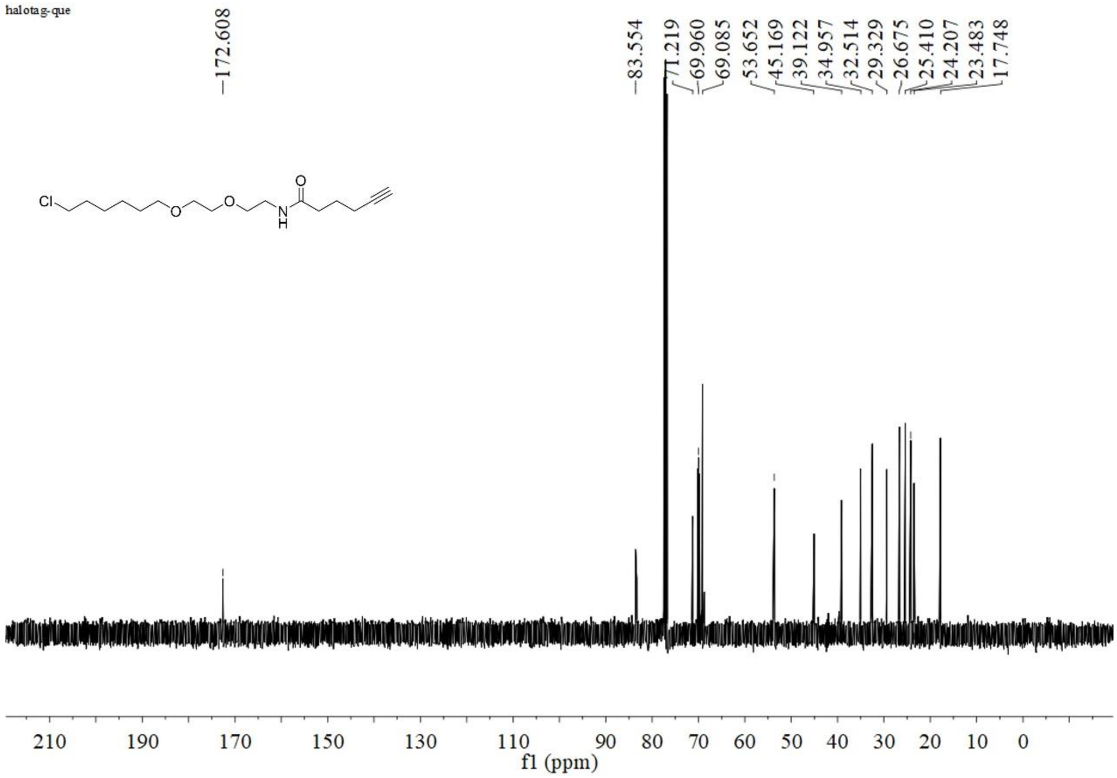

^{13}C NMR spectrum of compound **HTL1** in $CDCl_3$.

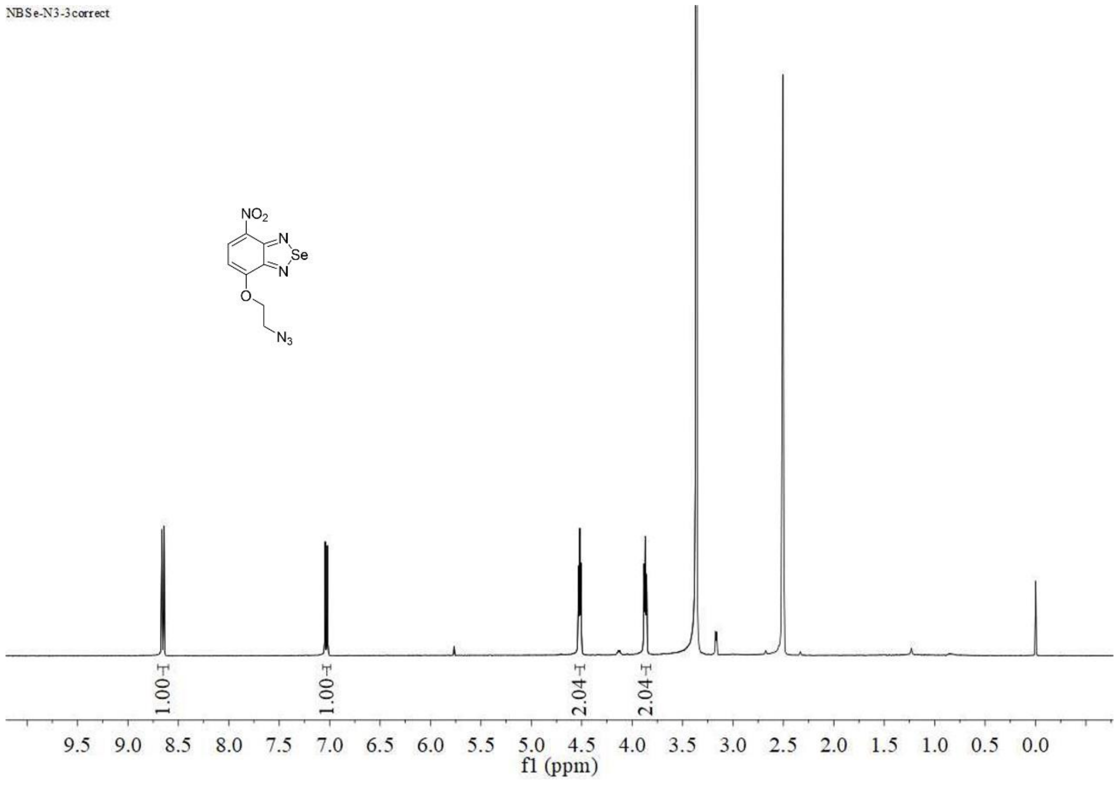

^{1}H NMR spectrum of compound **NBSe-N$_3$** in DMSO-d_6.

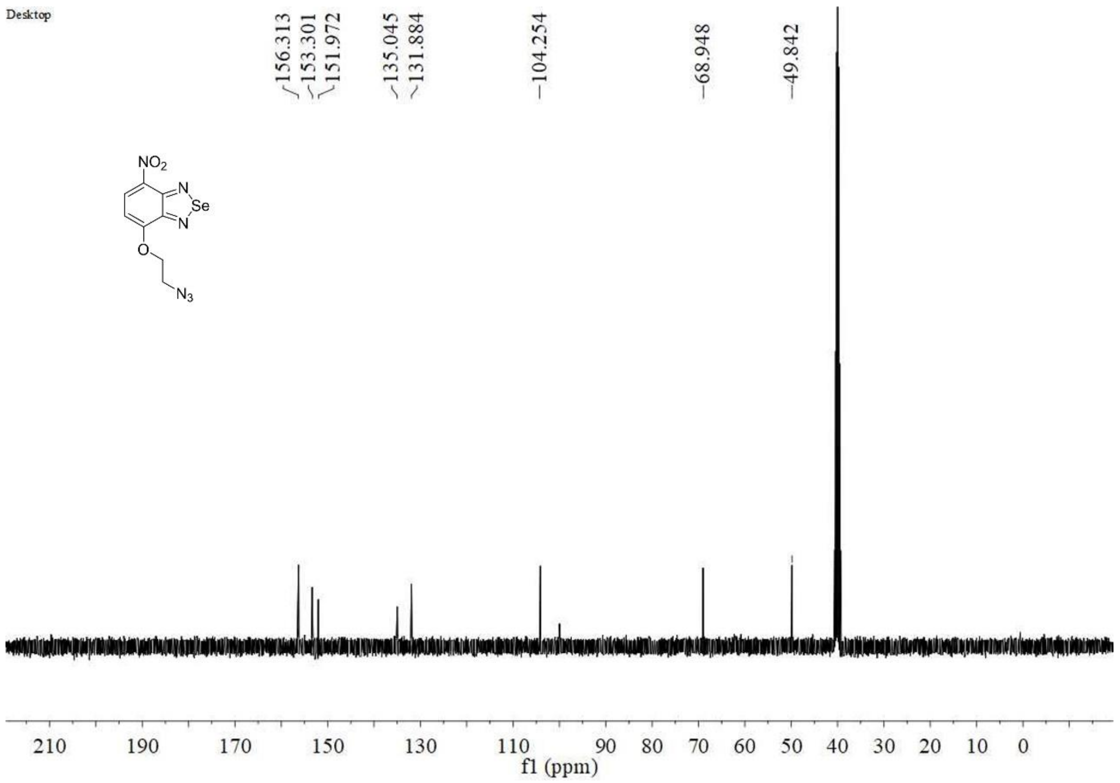

^{13}C NMR spectrum of compound **NBSe-N$_3$** in DMSO-d_6.

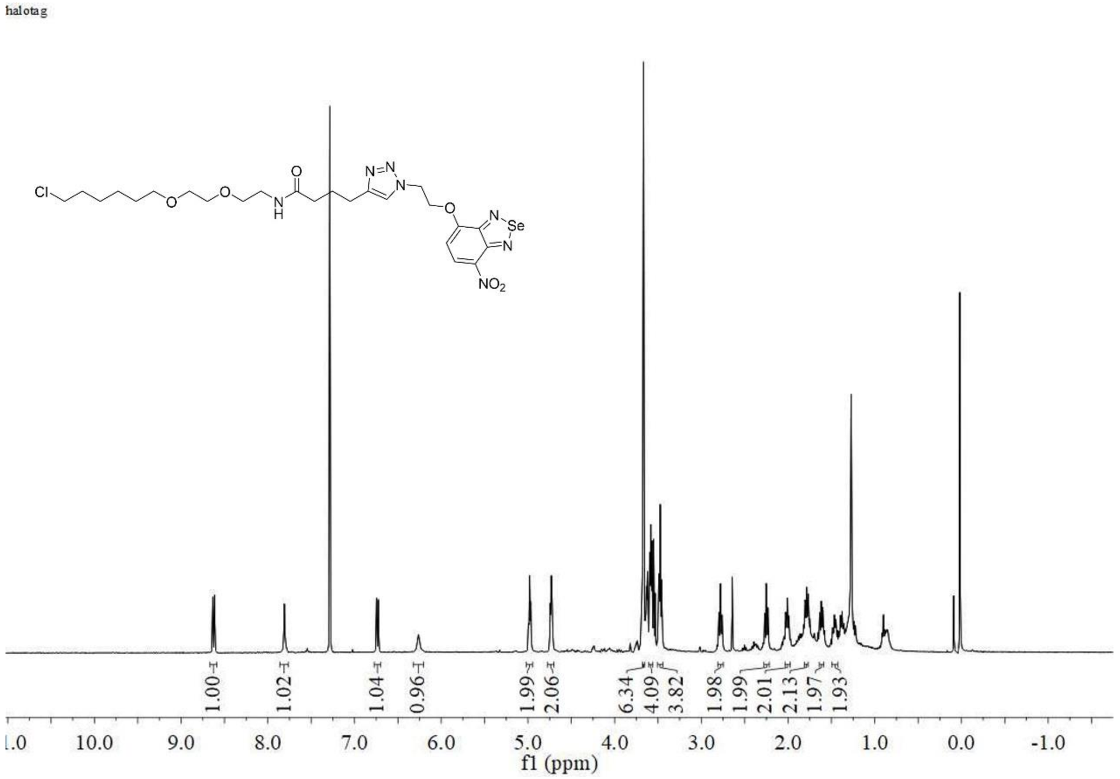

^{1}H NMR spectrum of compound **NBSe-HT** in $CDCl_3$.

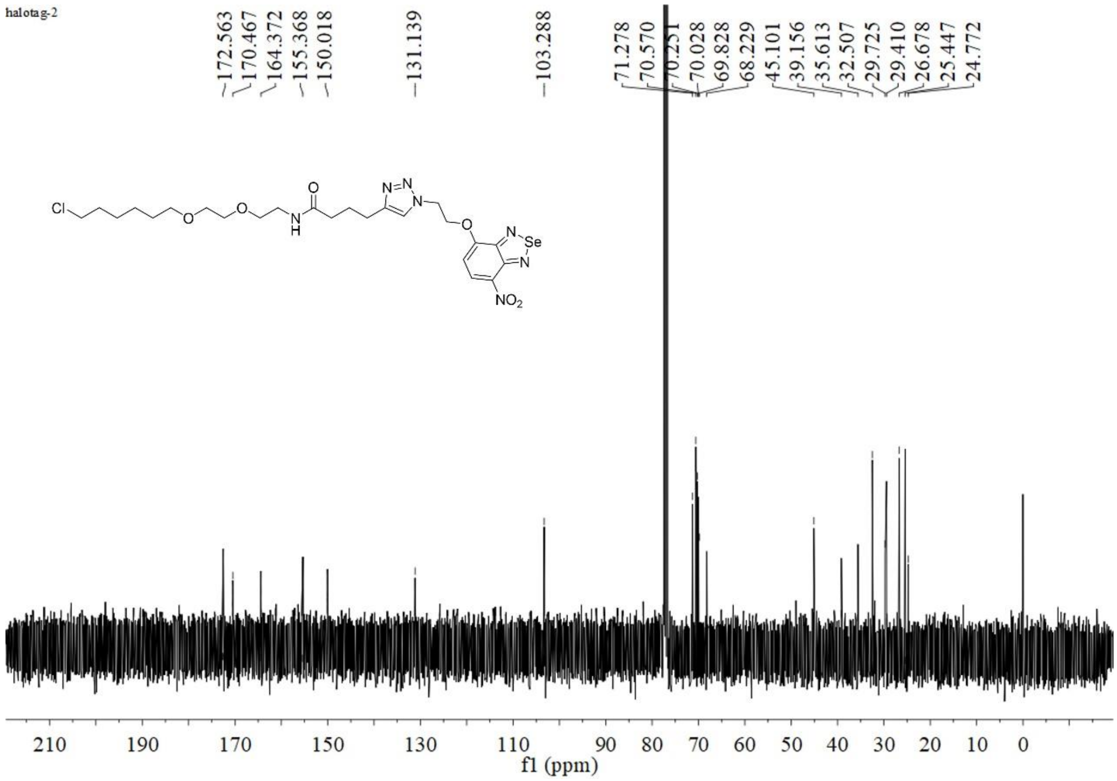

^{13}C NMR spectrum of compound **NBSe-HT** in $CDCl_3$.

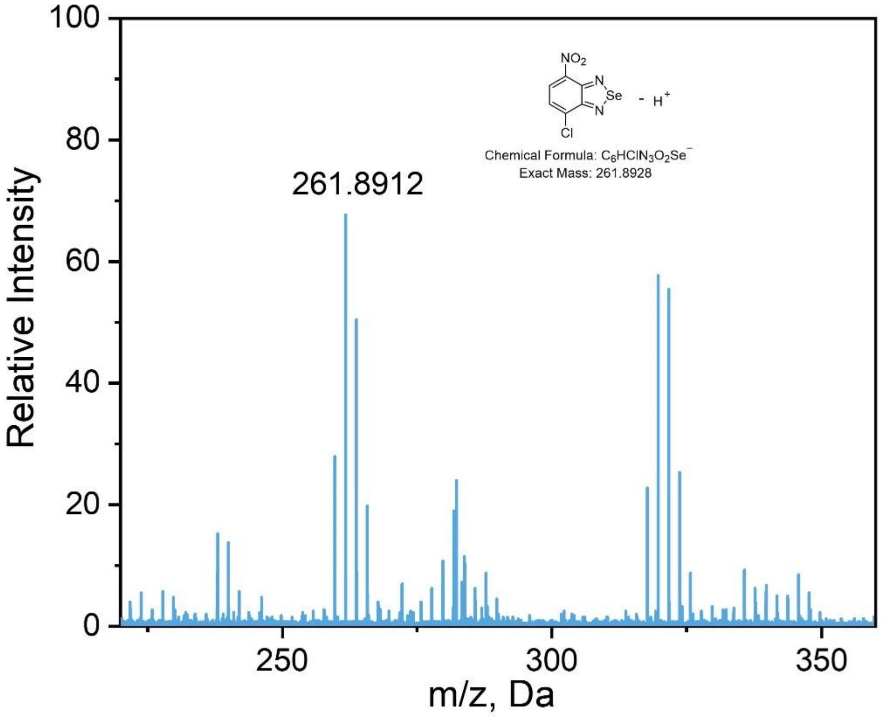

HRMS spectrum of compound **NBSe-Cl**.

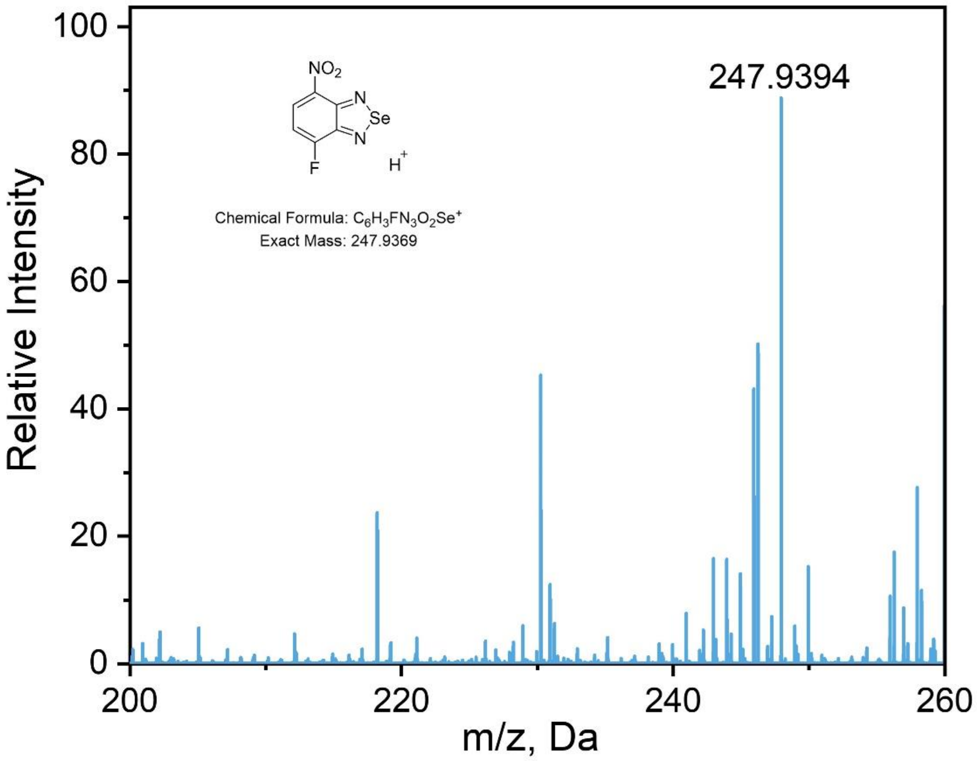

HRMS spectrum of compound **NBSe-F**.

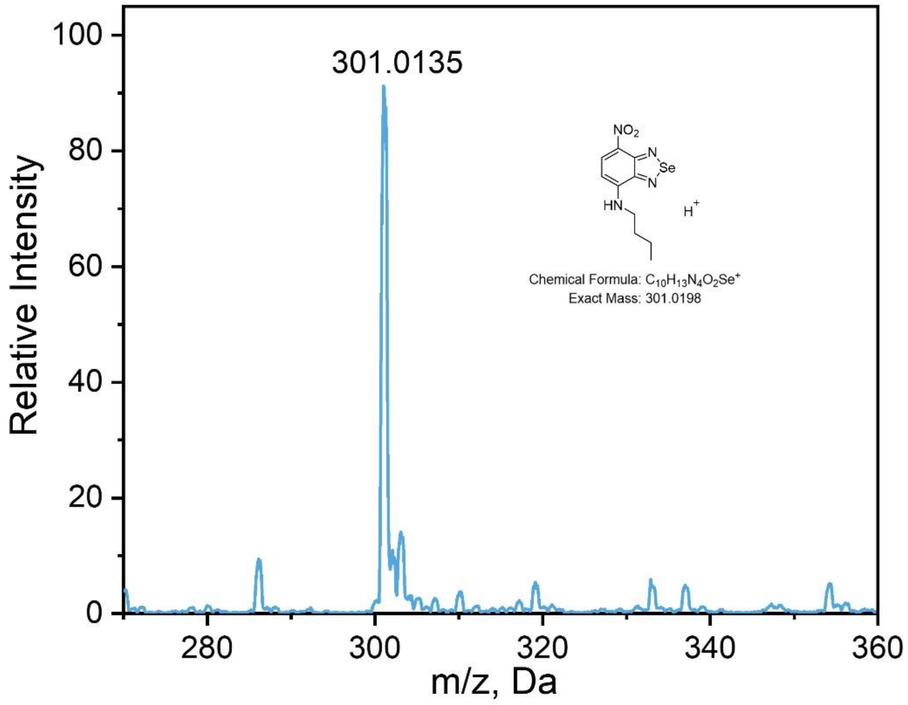

HRMS spectrum of compound **NBSe-N-nBu**.

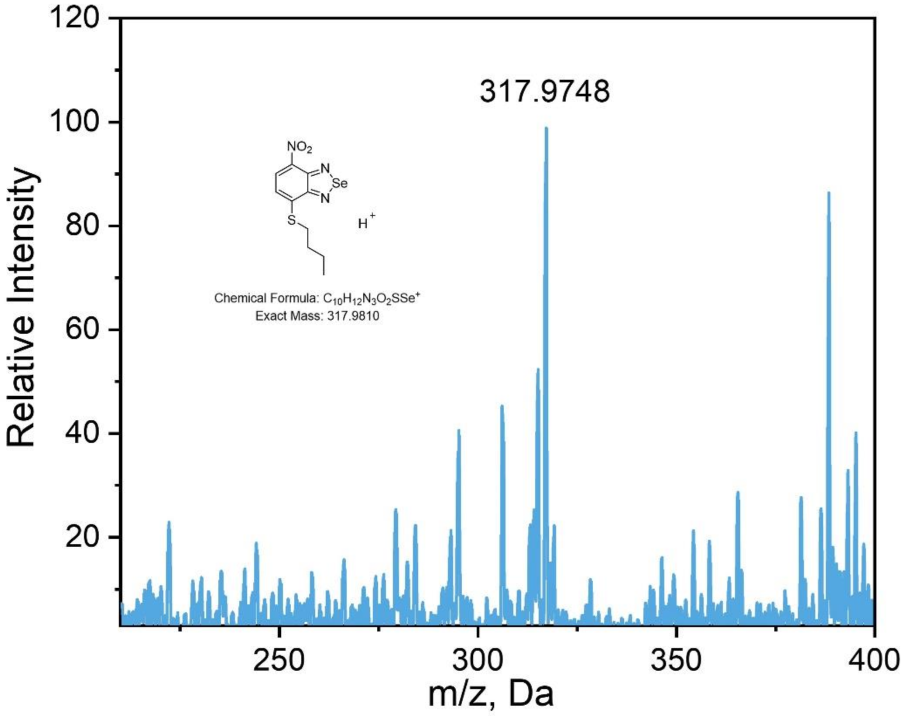

HRMS spectrum of compound **NBSe-S-nBu.**

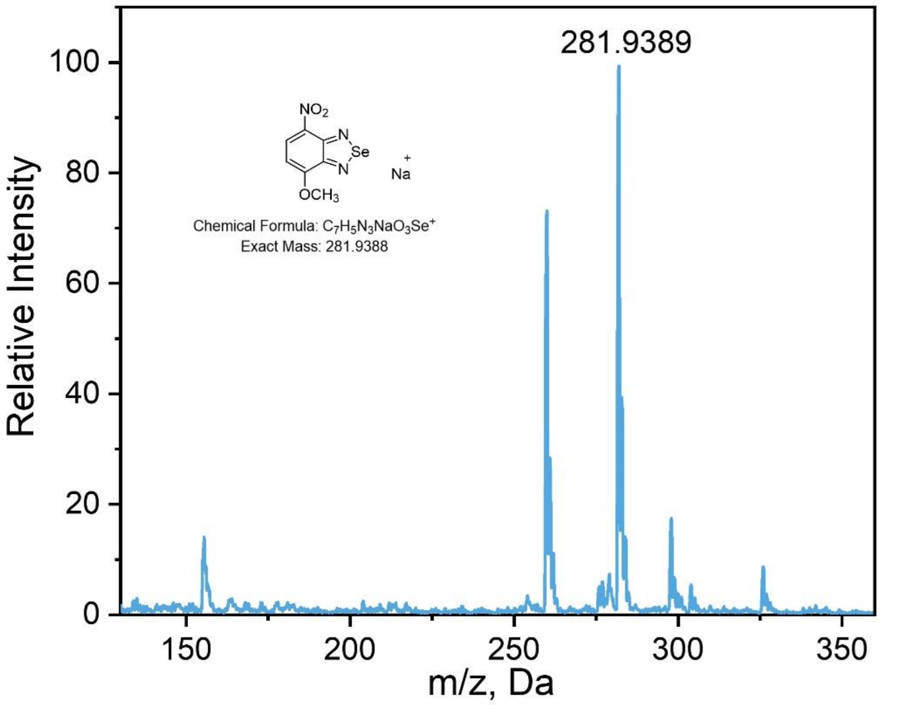

HRMS spectrum of compound **NBSe-OMe.**

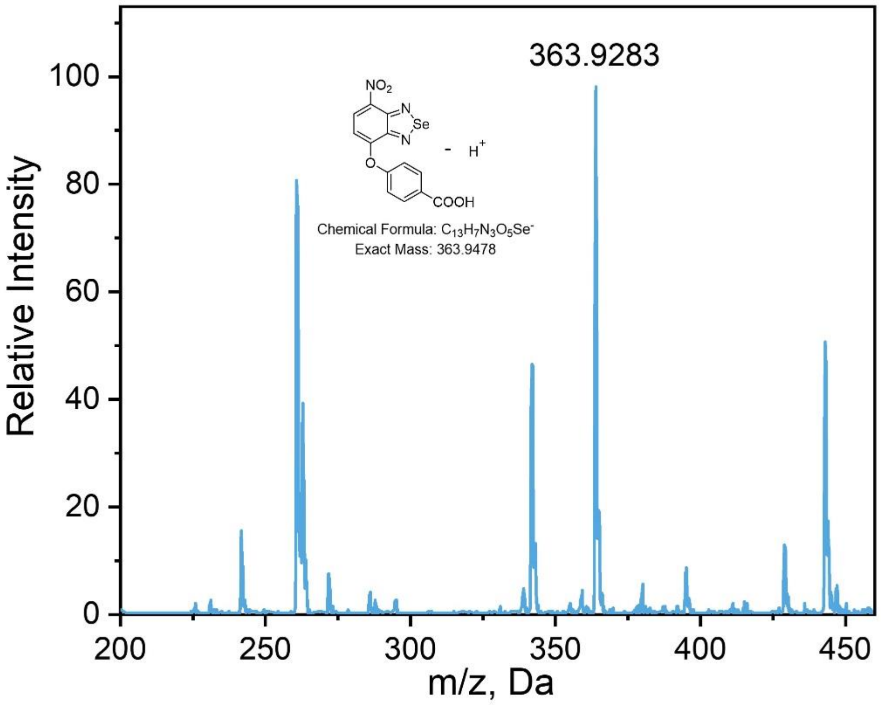

HRMS spectrum of compound **NBSe-OPh1**.

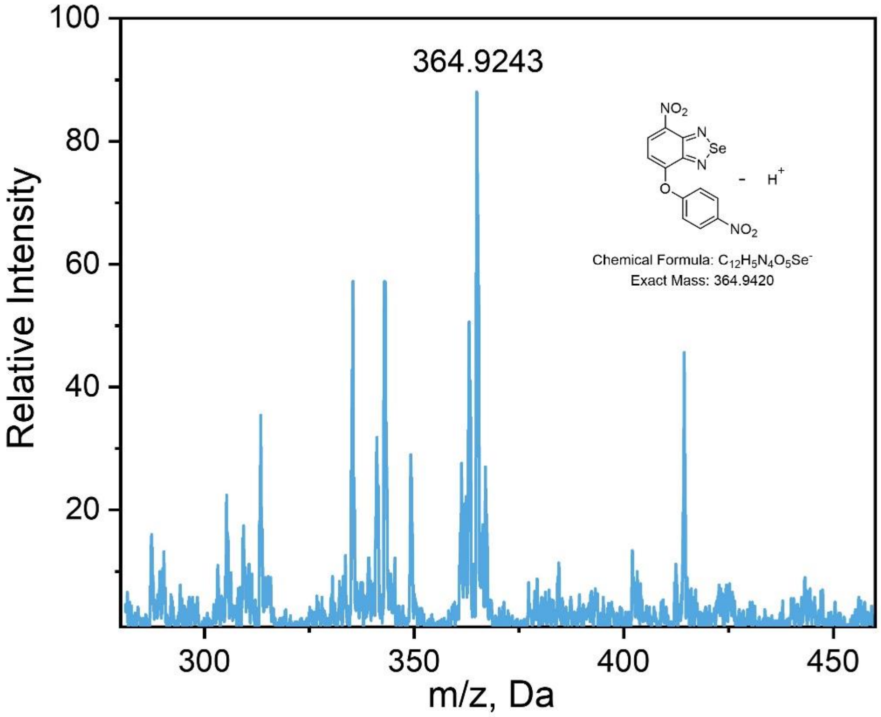

HRMS spectrum of compound **NBSe-OPh2**.

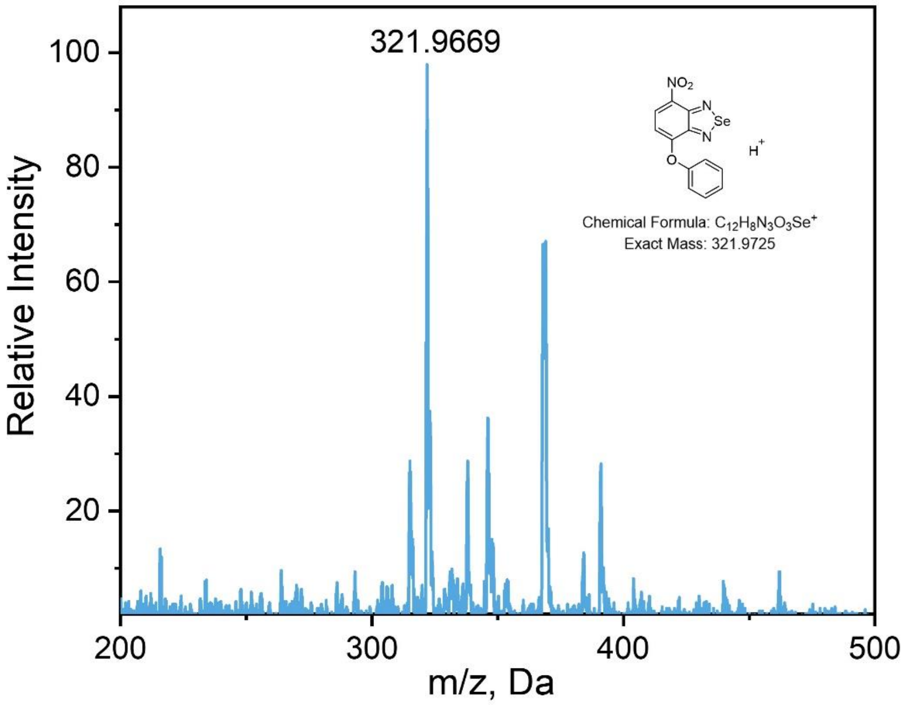

HRMS spectrum of compound **NBSe-OPh3**.

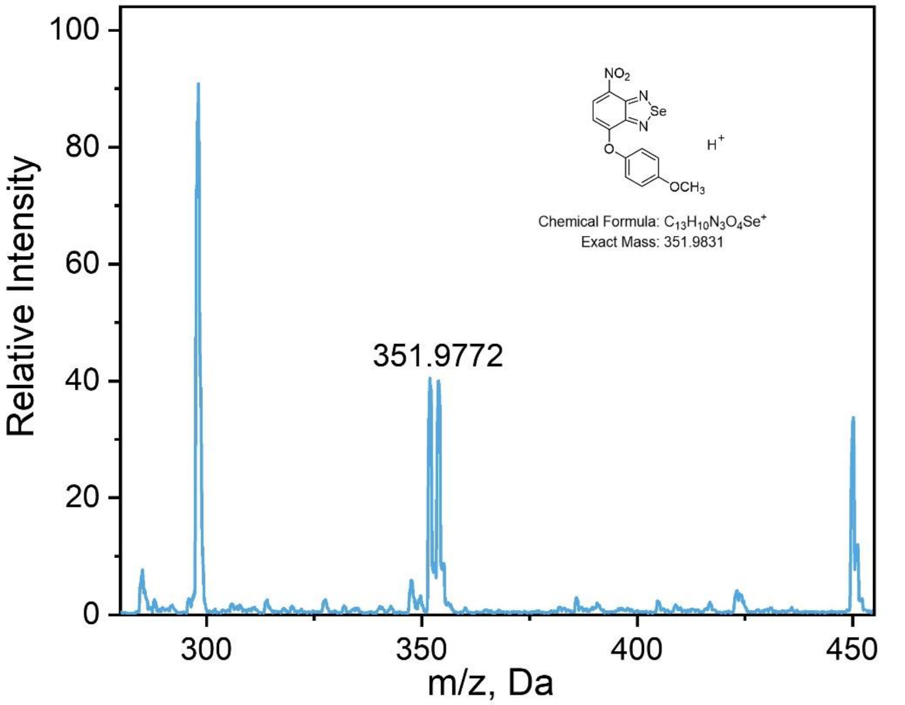

HRMS spectrum of compound **NBSe-OPh4**.

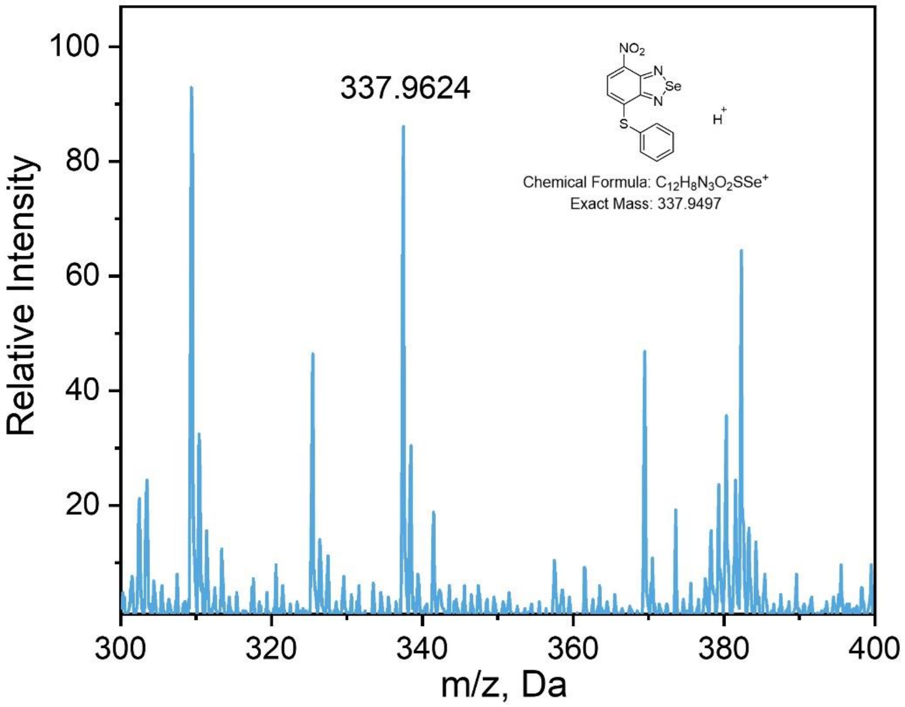

HRMS spectrum of compound **NBSe-SPh**.

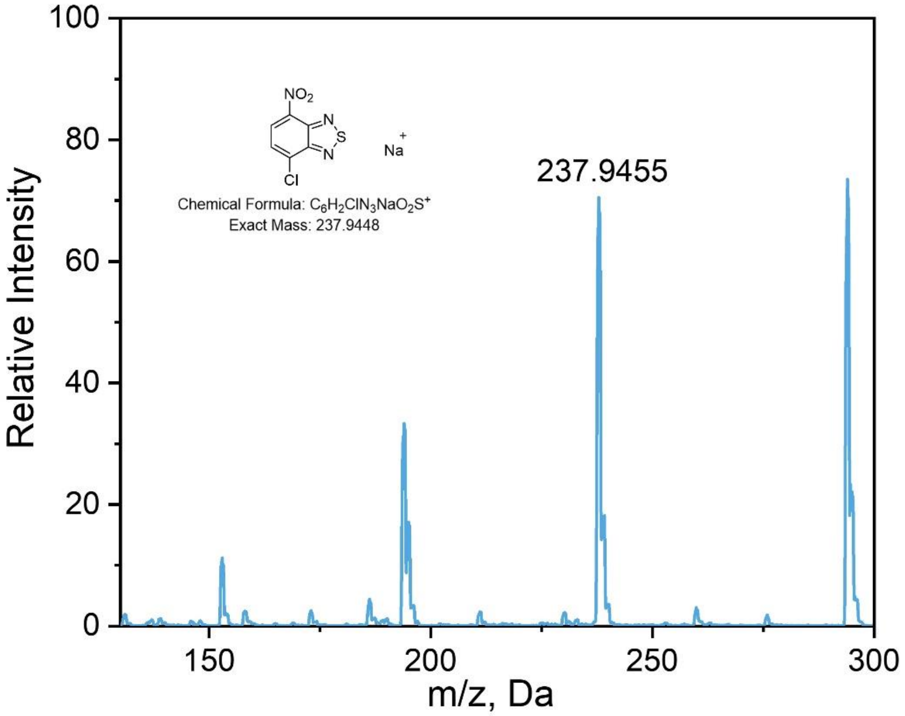

HRMS spectrum of compound **NBS-Cl**.

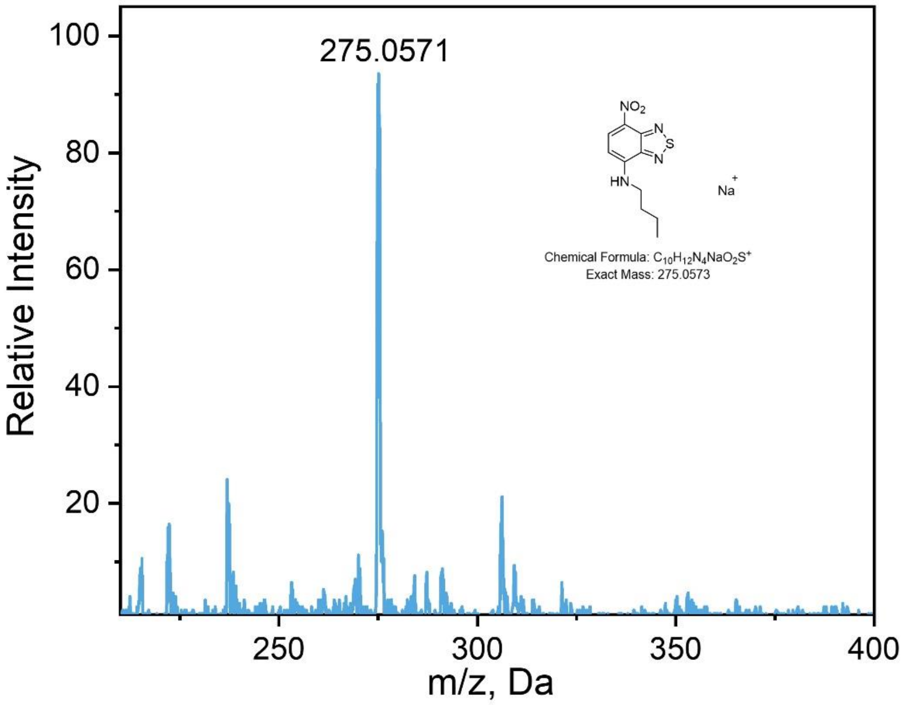

HRMS spectrum of compound **NBS-N-nBu**.

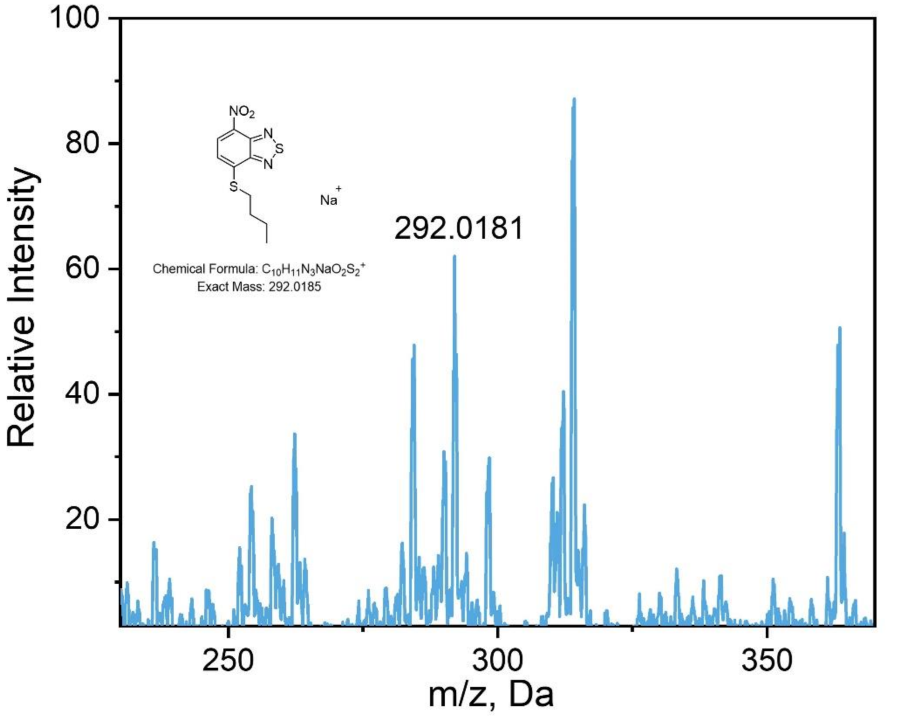

HRMS spectrum of compound **NBS-S-nBu**.

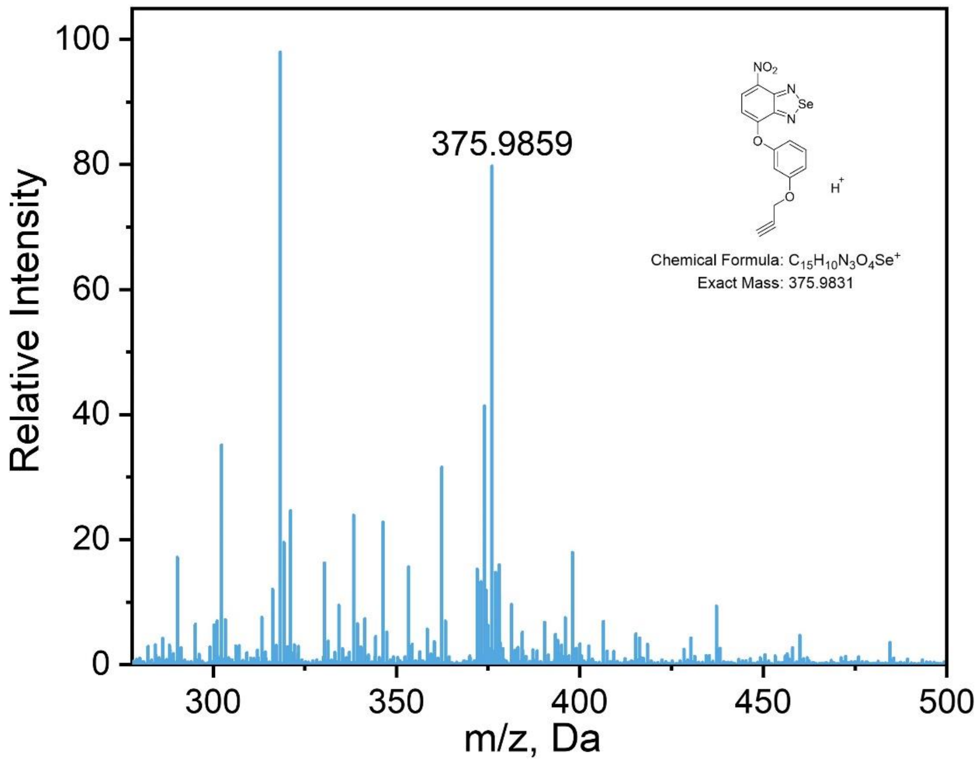

HRMS spectrum of compound **B2**.

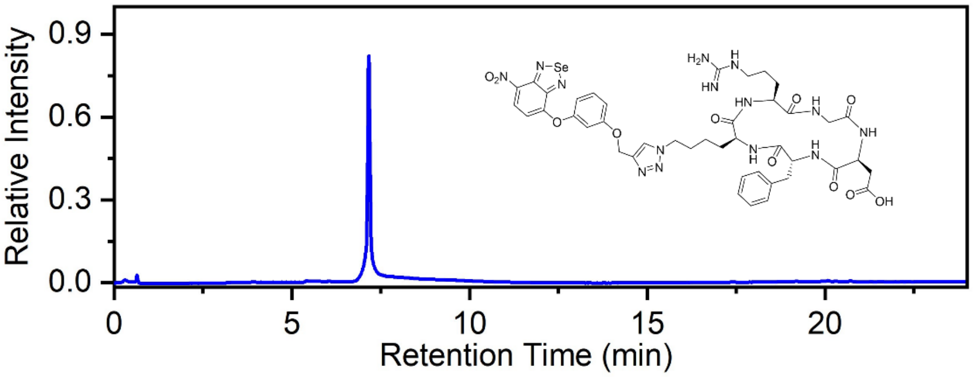

HPLC spectrum of compound **NBSe-cRGD**.

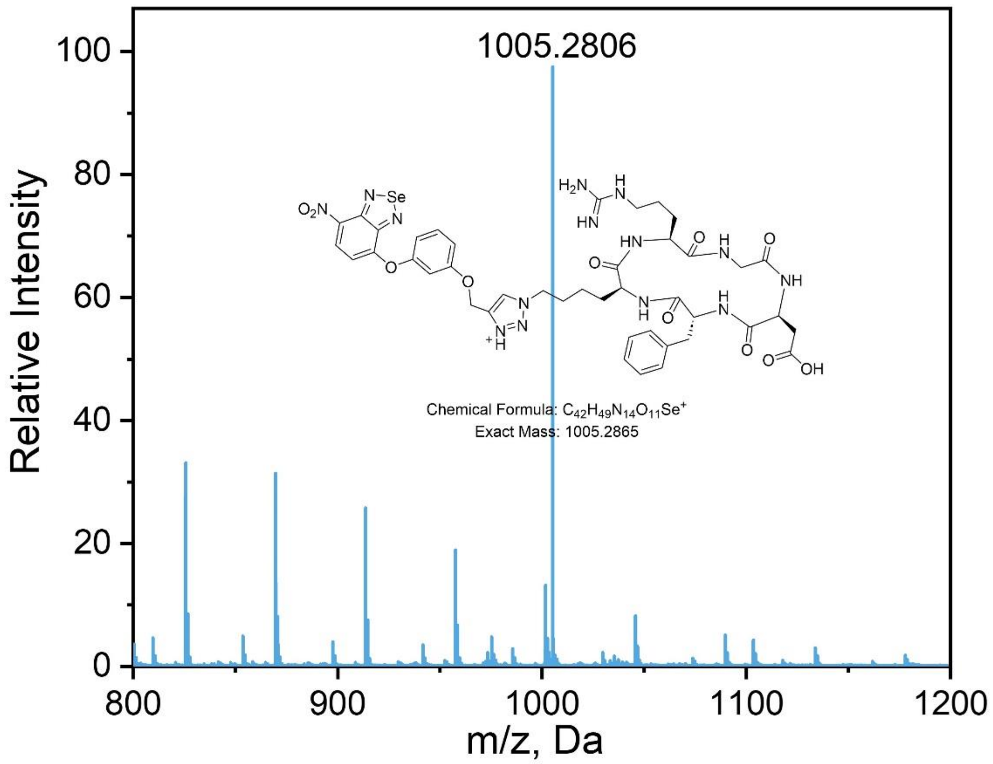

HRMS spectrum of compound **NBSe-cRGD**.

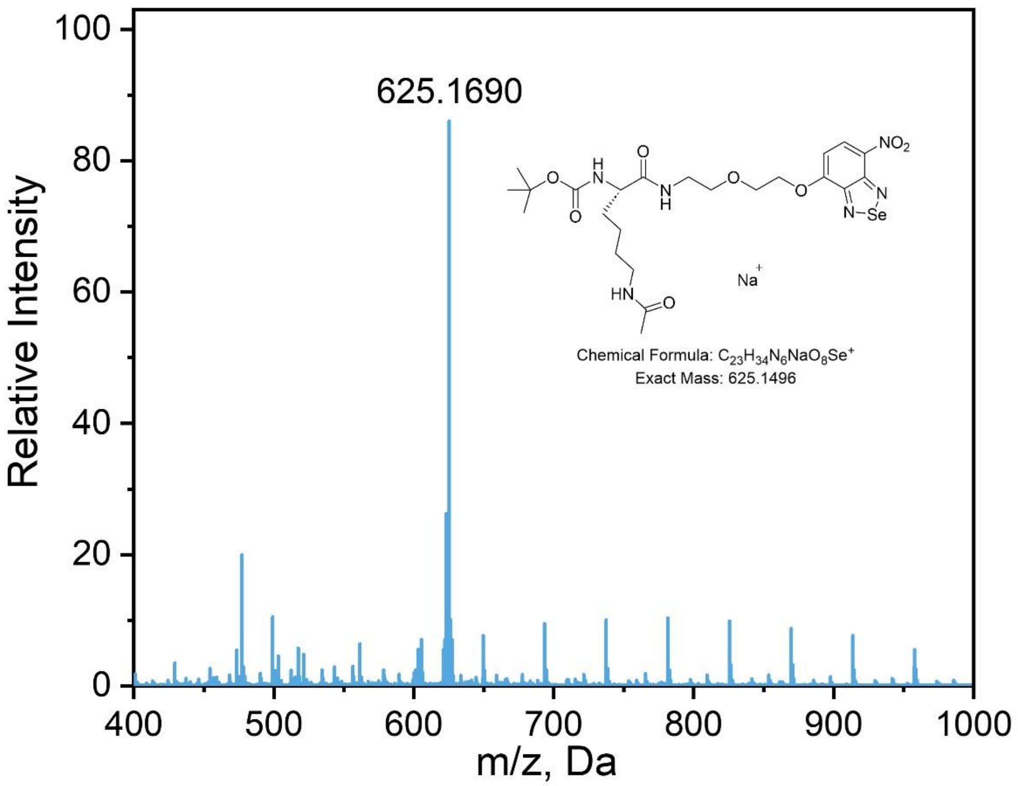

HRMS spectrum of compound **NBSe-HDAC**.

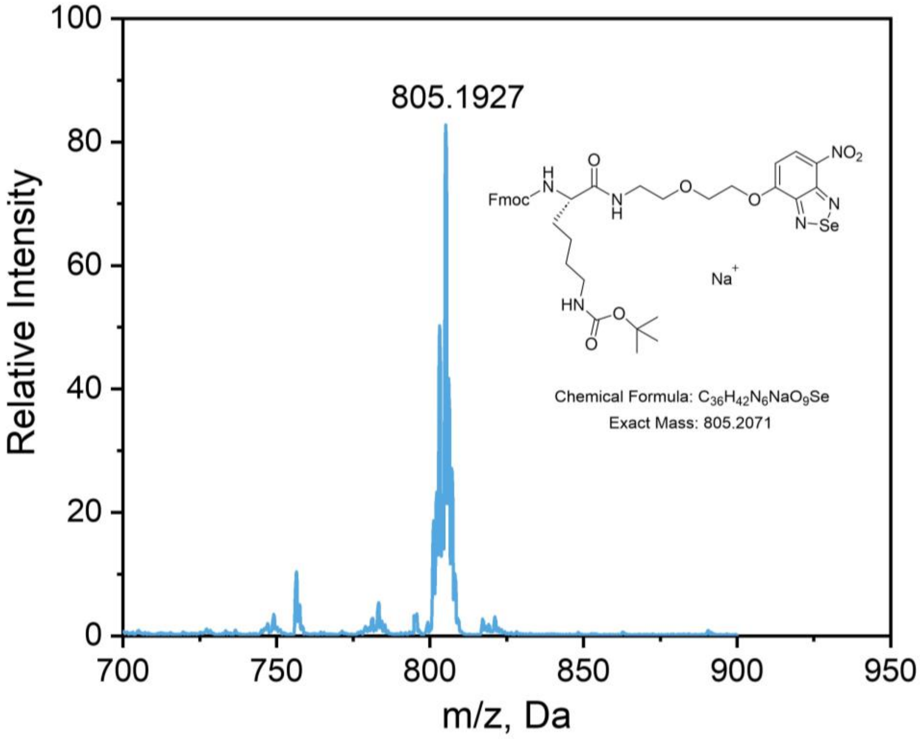

HRMS spectrum of compound **NBSe-MD**.

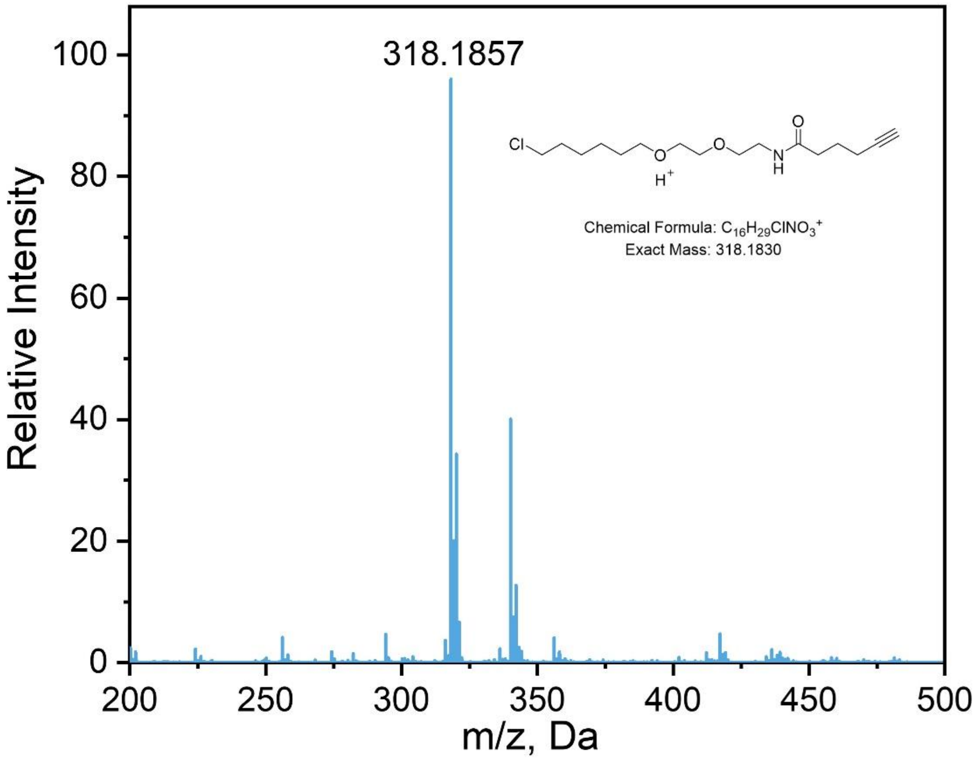

HRMS spectrum of compound **HTL1**.

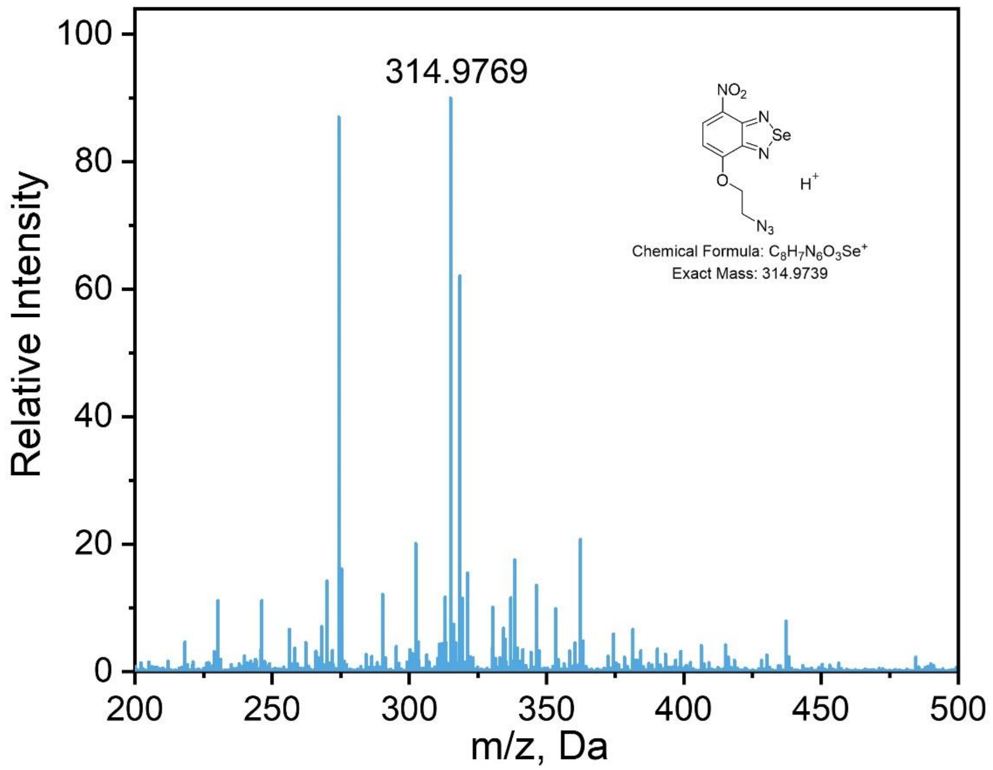

HRMS spectrum of compound **NBSe-N$_3$**.

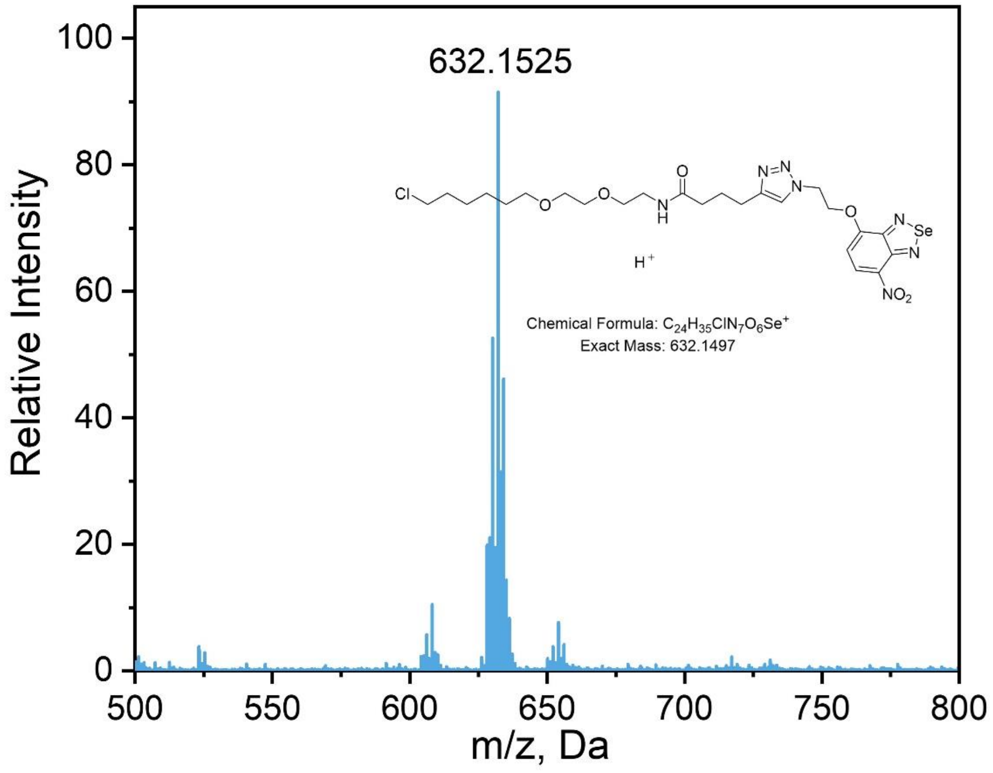

HRMS spectrum of compound **NBSe-HT**.

13. Reference

[1] F. Neese, *Wiley Interdiscip. Rev.: Comput. Mol. Sci.* **2022**, *12*, e1606.

[2] J. Zheng, X. Xu, D. G. Truhlar, *Theor. Chem. Acc.* **2011**, *128*, 295-305.

[3] T. Lu, F. Chen, *J. Comput. Chem.* **2012**, *33*, 580-592.

[4] M. Li, T. Xiong, J. Du, R. Tian, M. Xiao, L. Guo, S. Long, J. Fan, W. Sun, K. Shao, X. Song, J. W. Foley, X. Peng, *J. Am. Chem. Soc.* **2019**, *141*, 2695-2702.

[5] G. Weber, F. Teale, *Trans. Faraday Soc.* **1957**, *53*, 646-655.

[6] a) M. Kamiya, Y. Akahori, *Bull. Chem. Soc. Jpn.* **1970**, *43*, 268-271; b) J. Liu, Y.-Q. Sun, H. Zhang, H. Shi, Y. Shi, W. Guo, *ACS Appl. Mater. Interfaces* **2016**, *8*, 22953-22962; c) E. T. Strom, G.-A. Russell, *J. Am. Chem. Soc.* **1965**, *87*, 3326-3329; d) R. L. Hill, M. Gouterman, A. Ulman, *Inorg. Chem.* **1982**, *21*, 1450-1455; e) J. Angyan, I. Csizmadia, R. Daudel, R. Poirier, *Chem. Phys. Lett.* **1986**, *131*, 247-251.

[7] R. Moudgil, D. Kaur, R. Vashisht, P. V. Bharatam, *J. Chem. Sci.* **2000**, *112*, 623-629.

[8] C. Hansch, A. Leo, R. W. Taft, *Chem. Rev.* **1991**, *91*, 165-195.

[9] P. Atkins, P. W. Atkins, J. de Paula, *Atkins' Physical Chemistry*, Oxford University press, **2014**.

[10] Y. Hori, M. Nishiura, T. Tao, R. Baba, S. D. Bull, K. Kikuchi, *Chem. Sci.* **2021**, *12*, 2498-2503.

[11] a) M. Pan, H. Yuan, M. Brent, E. C. Ding, R. Marmorstein, *J. Biol. Chem.* **2012**, *287*, 2468-2476; b) S. Rymarchyk; W. Kang; Y. Cen, *Biomolecules* **2021**, *11*, 312.

[12] Y. Xie, J. Ge, H. Lei, B. Peng, H. Zhang, D. Wang, S. Pan, G. Chen, L. Chen, Y. Wang, Q. Hao, S. Q. Yao, H. Sun, *J. Am. Chem. Soc.* **2016**, *138*, 15596-15604.

[13] C. A. Foley, F. Potjewyd, K. N. Lamb, L. I. James, S. V. Frye, *ACS Chem. Biol.* **2020**, *15*, 290-295.
